# Bone Marrow CD34+/lin− Cells of Patients with Chronic-Phase Chronic Myeloid Leukemia (CP-CML) After 12 Months of Nilotinib Treatment Exhibit a Different Gene Expression Signature Compared to the Diagnosis and the Corresponding Cells from Healthy Subjects

**DOI:** 10.3390/cancers17061022

**Published:** 2025-03-18

**Authors:** Alessandra Trojani, Ester Pungolino, Barbara Di Camillo, Luca Emanuele Bossi, Cassandra Palumbo, Mariella D’adda, Alessandra Perego, Mauro Turrini, Chiara Elena, Lorenza Maria Borin, Alessandra Iurlo, Simona Malato, Francesco Spina, Maria Luisa Latargia, Pierangelo Spedini, Salvatore Artale, Michela Anghilieri, Maria Cristina Carraro, Cristina Bucelli, Alessandro Beghini, Roberto Cairoli

**Affiliations:** 1 Department of Hematology and Oncology, ASST Grande Ospedale Metropolitano Niguarda, 20162 Milan, Italy; estermaria.pungolino@ospedaleniguarda.it (E.P.); lucaemanuele.bossi@ospedaleniguarda.it (L.E.B.); cassandra.palumbo@ospedaleniguarda.it (C.P.); francesco.spina@ospedaleniguarda.it (F.S.); roberto.cairoli@ospedaleniguarda.it (R.C.); 2 Department of Information Engineering, University of Padova, 35131 Padova, Italy; barbara.dicamillo@unipd.it; 3 Department of Hematology and Oncology, ASST Spedali Civili Brescia, 25123 Brescia, Italy; mariella.dadda@asst-spedalicivili.it; 4 Fondazione IRCCS San Gerardo dei Tintori, 20900 Monza, Italy; alessandra.perego@irccs-sangerardo.it (A.P.); lorenzamaria.borin@irccs-sangerardo.it (L.M.B.); 5 Department of Internal Medicine, Valduce Hospital, 22100 Como, Italy; mturrini@valduce.it; 6 Department of Hematology Oncology, Foundation IRCCS Policlinico San Matteo, 27100 Pavia, Italy; chiara.elena1@gmail.com; 7 Hematology Division, Fondazione IRCCS Ca’ Granda Ospedale Maggiore Policlinico, 20122 Milan, Italy; alessandra.iurlo@policlinico.mi.it (A.I.); cristina.bucelli@policlinico.mi.it (C.B.); 8 Hematology and Bone Marrow Transplantation Unit, San Raffaele Scientific Unit, 20123 Milan, Italy; malato.simona@hsr.it; 9 ASST Valle Olona Ospedale di Circolo, 21052 Busto Arstizio, Italy; marialuisa.latargia@asst-valleolona.it; 10 ASST Cremona, 26100 Cremona, Italy; pierangelo.spedini@asst-cremona.it; 11 ASST Brianza, 20871 Vimercate, Italy; salvatore.artale@asst-brianza.it; 12 ASST Lecco, 23900 Lecco, Italy; m.anghilieri@asst-lecco.it; 13 ASST Fatebenefratelli Sacco, 20157 Milan, Italy; carraro.mcristina@hsacco.it; 14 Department of Health Sciences, University of Milano, 20146 Milan, Italy; alessandro.beghini@unimi.it

**Keywords:** Chronic-Phase Chronic Myeloid Leukemia (C-PCML), bone marrow CD34+/lin− cells, gene expression profiling (GEP), differentially expressed genes (DEGs), KEGG pathways

## Abstract

Background: Chronic-Phase Chronic Myeloid Leukemia (C-PCML) is defined by the presence of the *BCR-ABL1* fusion gene, which encodes a tyrosine kinase protein that drives the uncontrolled proliferation and survival of leukemic stem cells (LSCs). Nilotinib, a tyrosine kinase inhibitor, targets the activity of BCR-ABL1 by reducing aberrant signaling pathways, which drive the regeneration of LSCs. Despite nilotinib’s action, a population of resilient LSCs persist in the bone marrow (BM) and can indeed drive relapse and progression in CML patients. Methods: Our study investigated the gene expression profiling (GEP) of BM CD34+/lin− cells from 79 CP-CML patients at diagnosis, compared to the BM CD34+/lin− cells from the same patients after 12 months of nilotinib treatment and to the normal counterpart cells from 10 donors (CTRLs). Results: GEP analyses identified 3012 significantly differentially expressed genes across these comparisons. Among these, we focused on certain key genes associated with eight crucial KEGG pathways: CML, cell cycle, JAK-STAT, PI3K-Akt, MAPK, Ras, NF-kB, and ABC transporters. Within these pathways, we observed the up-regulation of several genes at diagnosis compared to both 12 months of nilotinib treatment and the CTRLs. Conclusions: We observed that certain transcriptome features present at diagnosis persisted after 12 months of nilotinib treatment, compared to CTRLs. This suggests that nilotinib may exert selective pressure, potentially supporting the survival and self-renewal of LSCs. Future insights into these pathways could help identify therapeutic targets to improve outcomes in CML.

## Simple Summary

Nilotinib and other tyrosine kinase inhibitors (TKIs) target the BCR-ABL1 oncoprotein in Chronic Myeloid Leukemia (CML), reducing the growth of leukemic stem cells (LSCs) and promoting their death. However, some LSCs persist in the bone marrow (BM), which may lead to relapse and disease progression. We analyzed the gene expression profiling of BM CD34+/lin− cells from 79 Chronic-Phase CML patients at diagnosis and after 12 months of nilotinib treatment, comparing them to the healthy cells from 10 donors (CTRLs). Our study identified key genes involved in eight crucial biological pathways: CML, cell cycle, JAK-STAT, PI3K-Akt, MAPK, Ras, NF-kB, and ABC transporters. We observed a progressive down-regulation of the expression levels of several genes across these pathways from diagnosis to 12 months of nilotinib treatment and to CTRLs. This could suggest that nilotinib effects create selective pressure, potentially supporting the survival and self-renewal of LSCs.

## 1. Introduction

Chronic-Phase Chronic Myeloid Leukemia (C-PCML) is a malignant clonal hematopoietic stem cell disease characterized by the presence of the *BCR-ABL1* fusion gene. This fusion gene results from a reciprocal translocation between chromosomes 9 and 22, which leads to a small chromosome 22 called the Philadelphia chromosome. The *BCR-ABL1* oncogene encodes a constitutively active tyrosine kinase, which activates multiple signaling pathways. These signaling mechanisms confer properties to the leukemic stem cells (LSCs) to proliferate, survive, and develop resistance to apoptosis [1].

Currently, treatment with tyrosine kinase inhibitors (TKIs) is the best choice for managing CML [2,3]. Nilotinib and other TKIs target the BCR-ABL1 oncoprotein, reducing the proliferation of LSCs and promoting their apoptosis. Inizio modulo TKIs have significantly improved outcomes, achieving overall survival rates of 82–95%. However, despite these advancements, 20–30% of CML patients develop resistance to TKIs over time, posing a challenge in long-term disease management [3,4].

Indeed, LSCs can develop resistance through a series of mechanisms, including mutations and overexpression of the *BCR-ABL1* fusion gene, deregulated expression of drug transporter genes, quiescence of LSCs, interaction with microenvironment, and activation of alternative signaling pathways that bypass the effects of targeted therapies, enabling LSCs to survive and proliferate [5]. Over one hundred *BCR-ABL1* point mutations can arise across various domains, including the phosphate-binding loop (P-loop), activation loop (A-loop), catalytic domains (C-loop), drug contact site (directly affecting TKIs binding), and the myristate pocket. These mutations can impact the efficacy of TKIs and contribute to resistance in CML treatment [3,6,7].

TKIs target the kinase activity domain of BCR-ABL1 but fail to eradicate LSCs, which can survive through various oncogenic intracellular signaling pathways, including NF-kB, JAK-STAT, Ras/MAPK, Src, TP53, PI3K-Akt, and the cell cycle [3,6,8]. Further investigations into these mechanisms are crucial for developing more effective strategies to overcome resistance and enhance outcomes for CML patients. Ongoing investigations aim to identify new therapeutic targets to eradicate the survival and persistence of LSCs in CML.

Previously, we conducted a multicenter study involving newly diagnosed C-PCML patients treated with nilotinib 300 mg BID. This study included 80 patients with C-PCML at diagnosis and after 12 months of nilotinib treatment. Our findings revealed that nilotinib led to the early clearance of the bone marrow (BM) CD34+/lin−Ph+ cells in newly diagnosed C-PCML patients [9,10]. Moreover, we analyzed the gene expression profiling (GEP) of BM CD34+/lin− cells from 80 C-PCML patients at diagnosis compared to those after 12 months of nilotinib treatment. GEP results showed that nilotinib interfered with the expression profile of certain genes associated with cell cycle, ATP-binding cassette (ABC) transporters, and JAK-STAT signaling pathway [11].

This study aimed to investigate the differentially expressed genes (DEGs) of BM CD34+/lin− cells from 79 C-PCML patients at diagnosis compared to those after 12 months of nilotinib treatment, as well as to the same cell counterparts from 10 healthy donors (CTRLs). Our research goal was to determine whether BM CD34+/lin− cells from 79 C-PCML patients after 12 months of nilotinib treatment exhibited a transcriptome signature similar to that of BM CD34+/lin− cells from CTRLs or if they displayed a distinct transcriptome profile. Our GEP analyses highlighted the up-regulation of several genes associated with eight Kyoto Encyclopedia of Genes and Genomes (KEGG) pathways at diagnosis compared to both 12 months of nilotinib treatment and CTRLs. These pathways play a key role in the proliferation, survival, and differentiation of CML LSCs.

## 2. Materials and Methods

### 2.1. Patients

In this study, we selected BM CD34+/lin− cells from 79 C-PCML patients at diagnosis, from the same patients after 12 months of nilotinib treatment, and from 10 CTRLs. These 79 CP-CML patients were part of a previous study, the PhilosoPhi34 study, which included 87 CP-CML patients across 15 centers in Italy [9]. Samples were collected from consenting patients on behalf of the Rete Ematologica Lombarda (REL) [9]. The participants provided their written consent to participate in this study, which was approved by the Ethics Committee ASST Grande Ospedale Metropolitano Niguarda (Milan, Italy) (EudraCT: 2012-005062-34 on 14 December 2012). These patients were treated with first-line therapy consisting of nilotinib 300 mg twice daily. Eighty out of eighty-seven patients could be investigated. Among these 80 patients, only one experienced relapse at 12 months of treatment [9,10]. At diagnosis, FISH analysis detected BM CD34+/lin−Ph+ cells in all 80 C-PCML patients. At 12 months, we could analyze 80/87 patients. Seventy-nine out of eighty patients could be evaluated because they achieved at least a complete cytogenetic response, whereas 1/80 patients relapsed at 12 months. No Ph+ nuclei were detected in 79/79 patients [9,10]. We performed qPCR in all 79 C-PCML patients at diagnosis and at 12 months of nilotinib treatment as per clinical practice (data not shown in accordance with company policy).

### 2.2. Isolation of BM CD34+/lin− Cells Using Immunomagnetic Beads

Mononuclear cells (MNCs) were isolated from BM blood samples (approximately 6 mL each) obtained from 10 CTRLs using Ficoll density gradient centrifugation at 800 rpm for 20 min at 4 °C. We selected BM CD34+/lin− cells using MACS Diamond CD34 Isolation kit human (130-094-531) and autoMACs Pro Separator (Miltenyi Biotec, Bologna, Italy) according to the manufacturer’s instructions (Miltenyi Biotec). Briefly, we labeled BM MNCs with a mix of biotin-conjugated antibodies against lineage-specific antigens. Immediately afterward, these cells were labeled with Anti-Biotin Microbeads. BM CD34+/lin− cells were obtained from the lineage-negative stem and progenitor cells using CD34 Microbeads (Miltenyi Biotec). The purity of isolated BM CD34+/lin− cells was detected by flow cytometry. For more detailed methods, please refer to http://dx.doi.org/10.17504/protocols.io.yncfvaw, accessed on 19 July 2019 and our previous study [11].

### 2.3. Cell Cryopreservation and RNA Extraction

The BM CD34+/lin− cells of each subject were preserved in 50 μL of RNAlater (Thermo Fisher Scientific, Milano, Italy) and stored at −20 °C until RNA extraction, following the procedure previously described [12]. Total RNA extraction from the BM CD34+/lin− cells stored in RNAlater was carried out using the MagMAX^TM^-96 Total RNA Isolation Kit (Thermo Fisher Scientific), following the manufacturer’s instructions. The quality and yield of the extracted RNA were assessed using a NanoDrop^TM^ 2000/2000c spectrophotometer (Thermo Fisher Scientific). For further details, please refer to http://dx.doi.org/10.17504/protocols.io.yncfvaw, accessed on 19 July 2019 [11].

### 2.4. GEP Experiments

Microarray experiments were conducted on the BM CD34+/lin− cells obtained from 10 CTRLs. Initially, cDNA was synthesized from the previously extracted RNA (50 ng) using the Ovation Pico WTA System V2 kit (NuGEN) and the Encore Biotin Module Kit (NuGEN), following the manufacturer’s protocols.

The prepared cDNA was then hybridized to the Affymetrix HTA 2.0 array using the Gene Chip platform (Affymetrix, Santa Clara, CA, USA). Subsequently, the signals were scanned using the Affymetrix Gene Chip Scanner 3000, following the manufacturer’s instructions. For detailed procedures, please refer to http://dx.doi.org/10.17504/protocols.io.yncfvaw, accessed on 19 July 2019 and our previous manuscript [12].

### 2.5. Bioinformatic Analyses of GEP Data

The processing of the microarray raw data was performed using the R statistical computing software (http://www.r-project.org, accessed on 11 March 2025). The data were normalized using the “RMA” method from the “oligo” package (version 1.42.0) from the Bioconductor repository (version 3.4) [13,14]. In this step, the measured signal intensities of the 6 million probes were summarized into probe sets specific for a given gene locus, realizing a one-to-one assignment of probe sets and genes. Affymetrix microarray data are one-color arrays that represent absolute signal intensities from gene probes. Data were first pre-processed using ComBat to adjust for batch effects and quantile normalization [15]. We investigated differential expressions to compare the three groups of samples using the SAM test [16]. The first group was composed of the BM CD34+/lin− cells of 79 C-PCML patients at diagnosis, and the second one consisted of the BM CD34+/lin−cells of the same patients after 12 months of nilotinib treatment [11]. The third group comprised the BM CD34+/lin− cells of 10 CTRLs. False Discovery Rate (FDR) adjusted p-values below 5% were considered significant [17]. Selection of DEGs was performed using SAM on the three groups and a Benjamini–Hochberg false discovery rate threshold of 5%, followed, for significance comparisons, by a pair-wise SAM test. Only annotated probe sets with at least a one-fold change greater than 1.5 were considered for the following analysis steps. Probe sets were grouped considering four main patterns of expression:

- Pattern 1 with expression at diagnosis greater than expression at 12 months greater than expression in CTRLs;
- Pattern 2 with expression at diagnosis lower than expression at 12 months lower than expression in CTRLs;
- Pattern 3 with expression in CTRLs greater than expression at diagnosis greater than expression at 12 months;
- Pattern 4 with expression in CTRLs lower than expression at diagnosis lower than expression at 12 months;
- Other patterns not previously listed.

We then applied DAVID functional clustering to genes belonging to each pattern to classify them into functional groups based on their annotation term co-occurrence, limiting the results to those groups that were enriched according to a nominal p-value lower than 5% [18,19].

## 3. Results

The purity of BM CD34+/lin− cells was >97%, as determined by flow cytometry [11]. The purity of the extracted RNA was in the range of 1.8–2.1, determined by absorbance ratios of A(260)/A(280).

After microarray processing, we performed principal component analysis (PCA) and examined MvA plots, which represent gene expression differences between arrays (M) against their average (A) on a log scale. No batch effects or residual systematic differences among arrays were observed in the data. PCA and MvA plots are available as Supplementary Materials (Graphical plots S1).

GEP analyses were performed among three groups of subjects: 79 patients at diagnosis (group 1), the same patients after 12 months of nilotinib treatment (group 2), and 10 healthy CTRLs (group 3). We identified 3463 probe sets, corresponding to 3012 different genes, as differentially expressed. Among these, 3023 (2634 unique genes) belonged to Pattern 1, 280 (244 unique genes) to Pattern 2, 54 (51 unique genes) to Pattern 3, and 94 (83 unique genes) to Pattern 4 (see in Section 2.5). The remaining 12 probe sets belonged to other patterns not previously listed.

Functional enrichment clustering revealed several interesting functional groups of genes. Among others, in pattern 1, we focused on the cell cycle, mitotic cell cycle, cell cycle phase, cell division, mitosis, regulation of cell cycle, nucleotide-binding, ATP-binding, and chromatin. These molecular mechanisms included genes that were up-regulated and regulated at diagnosis compared to both 12 months of nilotinib treatment and the CTRLs.

In Pattern 2, we observed genes related to disulfide bonds, including *AREG*, *TNXB*, and *TNF*, which exhibited lower expression levels at diagnosis compared to both 12 months of nilotinib treatment and CTRLs.

### 3.1. KEGG Chronic Myeloid Leukemia (hsa05220)

Fourteen genes belonging to Chronic Myeloid Leukemia were differentially expressed among the three groups of subjects. At diagnosis, *BRAF*, *CDK6*, *CHUK*, *GADD45A*, *HDAC2*, *MDM2*, *NRAS*, *PIK3CA*, *PIK3CB*, *POLK*, *PTPN11*, *RB1*, *SOS1*, and *SOS2* were significantly overexpressed compared to both 12 months of nilotinib treatment and the CTRLs (Table S1). The up-regulation of these genes could be associated with uncontrolled proliferation and increased survival of LSCs. Among these genes, *CDK6* exhibited a high fold change (FC) of 2.8 when comparing 12 months of nilotinib treatment to the CTRLs and 3.9 when comparing diagnosis to CTRLs. Similarly, *HDAC2* showed a high FC of 2.6 and 3.8 under the same conditions, respectively. *HDAC2* is known for its involvement in chromatin remodeling and gene expression regulation in the context of the cell cycle. This data suggests the potential role of *HDAC2* as a therapeutic target, particularly given the efficacy of HDAC inhibitors in targeting LSCs in C-PCML patients receiving TKIs. Interestingly, we observed that the proto-oncogene *MDM2* was up-regulated at diagnosis and exhibited a progressive down-regulation at 12 months of nilotinib treatment and in CTRLs.

**Figure 1:**
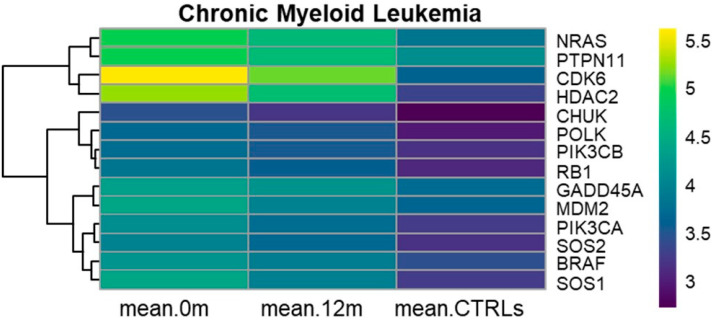
Heatmaps of Chronic Myeloid Leukemia. Heatmaps depicting the RMA-normalized expression levels (log2 scale) of DEGs in Chronic Myeloid Leukemia at month 0, after 12 months of nilotinib, and in CTRL subjects. Hierarchical clustering using average linkage was applied to the genes to enhance visualization.

This demonstrates its role in promoting tumorigenesis, particularly through its interaction with the p53 pathway at diagnosis. The down-regulation of *MDM2* may indicate the therapeutic effect of nilotinib on CML cells, although *MDM2* was still significantly overexpressed after 12 months of nilotinib treatment compared to CTRLs. Figure 1 illustrates the progressive underexpression of the 14 genes from diagnosis to 12 months of nilotinib treatment and to CTRLs.

### 3.2. KEGG Cell Cycle (hsa04110)

We observed that 54 genes related to the cell cycle were overexpressed at diagnosis compared to 12 months of nilotinib treatment and to CTRLs. Among these genes, *CDK6*, *HDAC2*, *SMC3*, *STAG2*, *YWHAE*, and *YWHAZ* exhibited high FC ranging from 2.2 to 3.9 in the comparison between 12 months vs. CTRLs and diagnosis vs. CTRLs, respectively (Table S1). These genes are figured out in all phases of the cell cycle (G1, S, G2, and M) (Figure S1).

### 3.3. KEGG PI3K-Akt Signaling Pathway (hsa04151)

Our analysis revealed the dysregulation of several genes (n = 41) associated with the PI3K-Akt signaling pathway in the comparison among the three groups of subjects (Figure S2).

Specifically, 36 out of 41 genes were found to be overexpressed at diagnosis compared to 12 months of nilotinib treatment and to CTRLs (Table S1). Among these, *HSP90AA1*, *HSP90AB1*, *HSP90B1*, *CDK6*, and *MYB* were significantly overexpressed at diagnosis compared to both 12 months of nilotinib treatment and CTRLs, with very high FC ranging from 2.6 to 5.5. Interestingly, *KIT* was similarly overexpressed both at diagnosis and after 12 months of nilotinib treatment compared to CTRLs (Table S1). Conversely, *AREG* and *TNXB* were down-regulated at diagnosis and after 12 months of nilotinib treatment compared to CTRLs (Table S1). Notably, *FLT3* and *MCL1* were overexpressed after 12 months of nilotinib treatment compared to both diagnosis and CTRLs (Table S1).

### 3.4. KEGG MAPK Signaling Pathway (hsa04010)

We observed the up-regulation of several genes (n = 41) belonging to the MAPK signaling pathway (Figure S3, Table S1). Specifically, *PRKACB*, *RAP1B*, and *STMN1* exhibited high FC ranging from 2 to 4.2 in the comparisons between CTRLs vs. diagnosis and CTRLs vs. 12 months of nilotinib treatment, respectively. These three genes are involved in various cellular processes regulated by the MAPK pathway, including cell proliferation, survival, and cytoskeletal dynamics. Notably, our analysis revealed that 7 out of 41 genes were similarly overexpressed both at diagnosis and after 12 months of nilotinib treatment compared to CTRLs.

### 3.5. KEGG JAK-STAT Signaling Pathway (hsa04630)

**Figure 2:**
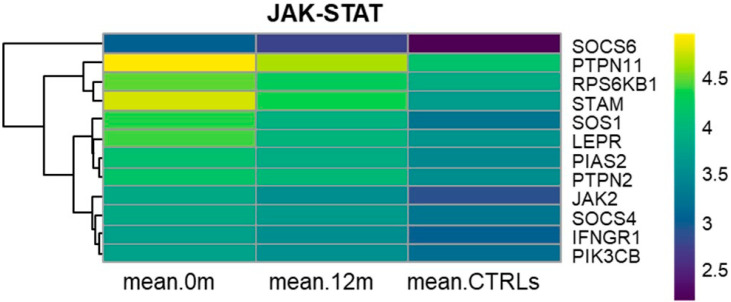
Heatmaps of JAK-STAT signaling pathway. Heatmaps depicting the RMA-normalized expression levels (log2 scale) of DEGs in the JAK-STAT signaling pathway at month 0, after 12 months of nilotinib, and in CTRL subjects. Hierarchical clustering using average linkage was applied to the genes to enhance visualization.

GEP results highlighted the deregulation of the JAK-STAT signaling pathway, which plays a key role in regulating various cellular processes, including cell proliferation, survival, and differentiation, ultimately leading to aberrant signaling cascades in CML. *JAK2*, *SOS1*, *PIK3CB*, *STAM*, *PTPN11*, *PTPN2*, *SOCS6*, *SOCS4*, *LEPR*, *IFNGR1*, *PIAS*, and *RPS6KB1* were all significantly overexpressed at diagnosis and showed progressively down-regulation after 12 months of nilotinib treatment, as well as in CTRLs (Figure 2, Table S1).

### 3.6. KEGG NF-kB Signaling Pathway (hsa04064)

**Figure 3:**
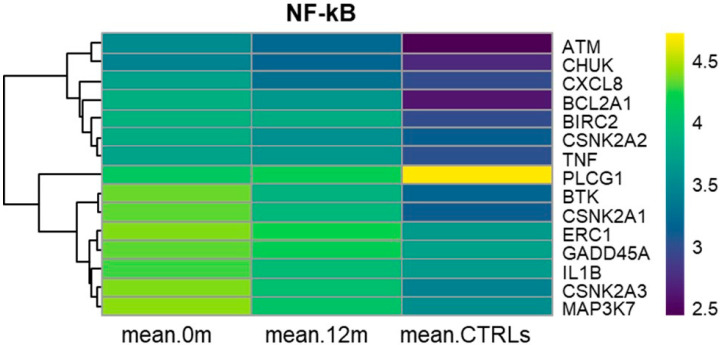
Heatmaps of NF-kB signaling pathway. Heatmaps depicting the RMA-normalized expression levels (log2 scale) of DEGs in the NF-kB signaling pathway at month 0, after 12 months of nilotinib, and in CTRL subjects. Hierarchical clustering using average linkage was applied to the genes to enhance visualization.

Fifteen genes belonging to the canonical (*ATM, IL1B*, *TNF*, *BTK*, *BIRC2*, *MAP3K7*, *CHUK*, *ERC1*, *PLCG1*, *GADD45A,* and *CXCL8*), noncanonical (*CHUK* and *BIRC2*), and atypical (*BIRC2*, *CSNK2A1*, *CSNK2A2*, *CSNK2A3*, and *BCL2A1*) NF-kB signaling pathway, were significantly and progressively down-regulated from diagnosis to 12 months of nilotinib treatment and to CTRLs (Table S1). In particular, *BCL2A1*, *TNF*, and *BIRC2* were similarly overexpressed both at diagnosis and after 12 months of nilotinib treatment compared to CTRLs. On the other hand, *PLCG1* expression remained at lower levels in CML patients at diagnosis and after 12 months of nilotinib treatment compared to CTRLs. *PLCG1* plays a role in cell signaling and may be involved in maintaining normal cellular functions. Its decreased expression in CML patients may suggest a potential dysregulation of certain signaling pathways associated with disease progression. Figure 3 clearly illustrates the progressive down-regulation of all these genes (except for *PLCG1*) from diagnosis to 12 months of nilotinib treatment and to CTRLs.

### 3.7. KEGG Ras Signaling Pathway (hsa04014)

Our analysis showed that 25 out of 26 genes exhibited a progressive decrease in expression levels from diagnosis to 12 months of nilotinib treatment and to CTRLs (Figure S4, Table S1). In particular, *ARF6*, *CALM2*, and *PRKACB* demonstrated high FC ranging from 2.5 to 3 in the comparisons between 12 months of nilotinib treatment and CTRLs and between diagnosis and CTRLs.

### 3.8. KEGG ABC Transporters (hsa02010)

**Figure 4:**
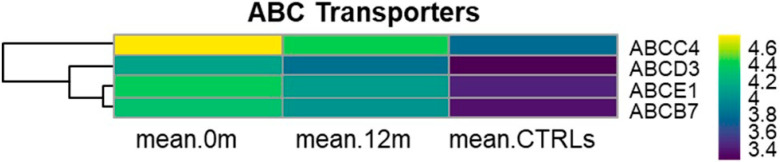
Heatmaps of ABC transporters. Heatmaps depicting the RMA-normalized expression levels (log2 scale) of DEGs in the ABC transporters at month 0, after 12 months of nilotinib, and in CTRL subjects. Hierarchical clustering using average linkage was applied to the genes to enhance visualization.

Our study revealed the progressive down-regulation of *ABCC4*, *ABCD3*, *ABCB7*, and *ABCE1* from diagnosis to 12 months of nilotinib treatment and to CTRLs (Figure 4, Table S1). These genes encode members of the ABC transporter superfamily, which are actively involved in transporting various molecules across cellular membranes. Notably, *ABCC4* and *ABCD7* belong to the multi-drug resistance-associated proteins (MRPs) subfamily of the ABC transporters. In particular, the overexpression of *ABCC4* in cancer cells can lead to reduced intracellular drug accumulation, contributing to multi-drug resistance in cancer chemotherapy [20].

## 4. Discussion

This study aimed to compare the transcriptome profiling of LSCs in a cohort of 79 C-PCML patients at diagnosis vs. 12 months of nilotinib treatment and vs. the same cell counterpart from 10 healthy subjects.

Transcriptome analyses highlighted that several genes associated with eight KEGG pathways demonstrated a trend of decreasing expression levels from diagnosis to 12 months of nilotinib treatment and to CTRLs. Notably, at 12 months of nilotinib treatment, most of the DEGs were significantly overexpressed compared to the CTRLs. Importantly, all of these pathways are related to cell survival, proliferation, and apoptosis in CML [21].

We observed that 14 genes within the Chronic Myeloid Leukemia were up-regulated at diagnosis compared to both 12 months of nilotinib treatment and CTRLs (Figure 1, Table S1). Specifically, we noticed the overexpression of *HDAC2* (hsa05220 and hsa04110) at diagnosis compared to both 12 months of nilotinib treatment and CTRLs with high FC, as shown in the results (Table S1). The dysregulation of *HDAC2* expression may affect therapeutic response, suggesting the potential use of HDAC inhibitors in CML patients [22,23,24,25]. Within the Chronic Myeloid Leukemia, *CDK6* (hsa05220 and hsa04110) was progressively down-regulated from diagnosis to 12 months of nilotinib treatment and further underexpressed in CTRLs, exhibiting a high fold change, as previously described (Table S1).

Schneeweiss-Gleixner et al. demonstrated that CDK4/6 inhibitors reduced *CD4*/*CDK6* expression and blocked the proliferation and survival of BCR/ABL1 positive CML cells, highlighting the potential benefit of this treatment for CML patients [26]. A recent in vitro study demonstrated that CDK6 competes with NRF-1 for promoter interaction, affecting mitochondrial respiration. Inhibition of CDK6 kinase activity alters mitochondrial morphology and the electron transport chain, leading to a metabolic shift toward glycolysis. Combined treatment with palbociclib and 2-deoxyglucose enhances apoptosis and blocks the proliferation of leukemic cells, including those resistant to imatinib. These findings suggest a potential therapeutic approach targeting metabolic vulnerabilities associated with high *CDK6* expression in CML [27].

In CML, the BCR-ABL1 protein alters cell cycle regulation by accelerating the passage of cells through cycle phases, thereby promoting uncontrolled cell proliferation. Toofan et al. showed that targeting CML CD34+ cells with Bone Morphogenetic Proteins (BMPs) receptor inhibitors in combination with TKIs interfered with cell cycle regulation, enhanced apoptosis, and reduced the number of CML CD34+ cells [28].

We highlighted a significant up-regulation of 54 genes associated with the cell cycle at diagnosis, compared to 12 months of nilotinib treatment and CTRLs (Figure S1, Table S1). This suggests that nilotinib may help down-regulate the cell cycle after 12 months of nilotinib treatment in our cohort of C-PCML patients. However, LSCs after 12 months of nilotinib treatment still exhibited significantly overexpressed genes compared to healthy cells, indicating the complexities of treatment and underscoring the need for further investigations.

The BCR-ABL1 fusion protein activates the PI3K-Akt signaling pathway in CML, which is crucial for promoting leukemic cell proliferation, survival, and metabolism, thereby contributing to the progression of CML [29].

Our study identified 36 genes in the PI3K-Akt signaling pathway, which were significantly overexpressed at diagnosis compared to 12 months of nilotinib treatment and CTRLs (Figure S2, Table S1). The up-regulation of *PIK3CA* and *MDM2* is particularly notable, as they are key players in leukemic cell survival by degrading p53.

Interestingly, previous research in clear cell ovarian carcinoma (CCOC) highlighted the association of both *MDM2* and *PIK3CA* overexpression with poor prognosis. This association suggests that targeting both the PI3K pathway and *MDM2* may offer a promising therapeutic strategy for CCOC [29,30,31].

Notably, Scott et al. showed that increasing p53 levels by inhibiting *MDM2*, particularly in combination with TKI therapy, significantly affected the engraftment potential of LSCs. This approach resulted in the loss of their ability to generate multiple cell lineages, leading to LSC exhaustion in CML cells over time [31].

Additionally, the BCR-ABL1 oncoprotein activates the c-KIT pathway, which is critical for the response of leukemic cells to TKIs in CML. This activation occurs through the phosphorylation of intracellular signaling molecules, including the Ras/MAPK and JAK-STAT signaling pathways, among others [21]. In an in vitro study, Airiau et al. showed that nilotinib-induced apoptosis was reduced by the addition of stem cell factor (SCF), which binds to its receptor c-KIT [32]. This indicated that SCF-mediated c-KIT activation diminished nilotinib’s effectiveness. However, the use of PI3K and mTOR pathway inhibitors restored the sensitivity of CML stem cells to nilotinib.

We found that *KIT* was overexpressed at diagnosis and after 12 months of nilotinib treatment compared to CTRLs (Table S1). This suggests that the transcription levels of *KIT* are similar in the BM CD34+/lin− cells at diagnosis and after 12 months of nilotinib. Future investigations will help to understand the use of TKIs in combination with PI3K/mTOR pathway inhibitors.

Moreover, this study underlined the up-regulation of 41 genes belonging to the MAPK signaling pathway at diagnosis compared to both 12 months of nilotinib and CTRLs (Figure S3, Table S1). Within the Ras signaling pathway, we observed that 25 out of 26 genes were up-regulated at diagnosis compared to 12 months of nilotinib treatment and CTRLs, indicating a strong activation of this pathway in leukemic cells (Figure S4, Table S1).

In the JAK-STAT signaling pathway, we observed that twelve key genes distributed along the pathway were overexpressed at diagnosis and progressively underexpressed after 12 months of nilotinib treatment and in CTRLs (Figure 2, Table S1).

Key receptors such as LEPR and IFNGR1 facilitate cytokine binding and the subsequent activation of STATs by JAK2. Thus, JAK2 activates the MAPK and PI3K-Akt signaling pathways by the phosphorylation of PTPN11 and PIK3CB, respectively.

The dysregulation of the JAK-STAT signaling pathway has been previously noted in various hematological disorders. This suggests that targeting components like JAK2 or STAT proteins may help inhibit aberrant signaling and leukemic cell growth, thereby improving treatment outcomes for CML patients [33]. Indeed, the use of JAK inhibitors in combination with nilotinib in CML has been shown to increase cell death and enhance the elimination of minimal residual disease in the BM [33,34,35].

The role of the NF-kB signaling pathway in CML is significant, as it contributes to B cell development, survival, resistance to chemotherapy, and invasion of the microenvironment [36]. In our study, among the DEGs associated with the NF-kB signaling pathway, *BCL2A1* and *BIRC2* are anti-apoptotic genes, while *TNF* is involved in inflammatory responses and can promote cell survival. The sustained up-regulation of these three genes at diagnosis and after 12 months of nilotinib treatment suggests they may contribute to the enhanced survival and proliferation of leukemic cells in CML.

TKIs’ response in CML could be influenced by polymorphic variants and/or changes in the expression levels of certain drug transporters genes that mediate the influx and efflux through the cells, thus regulating the pharmacokinetics and, consequently, the efficacy of these drugs. Berger et al. investigated the accumulation of nilotinib in CML CD34+ cells and polymorphonuclear cells and found that nilotinib uptake in primary C-PCML cells was dependent on the heterogeneous expression of membrane transporters among different cell types within the CML population [37].

In particular, they observed that the intracellular presence of nilotinib in immature CD34+ cells varied significantly, with nilotinib being undetectable in about 40% of the samples. This phenomenon could be attributed to resistance to nilotinib and its potential rejection through unknown mechanisms or possibly the action of ATP-binding cassette transporters, although this mechanism remains challenging to investigate.

Other studies indicated that CML CD34+ cells, as well as CML LSCs, displayed resistance to nilotinib and other TKIs [38,39]. Moreover, it seemed that the quiescent CD34+ cells were able to intake nilotinib as well [37].

These observations underscored that the accumulation of nilotinib was influenced by the intrinsic properties of CD34+ cells in each CML patient, as well as by cellular intra-clonal heterogeneity. Nevertheless, the action of nilotinib alone was not sufficient to abolish BCR-ABL activity.

We showed the up-regulation of *ABCC4*, *ABCD3*, *ABCB7*, and *ABCE1* in C-PCML patients at diagnosis, followed by a progressive underexpression after 12 months of nilotinib and in CTRLs as demonstrated in previous studies (Figure 4, Table S1) [11,20,37,40,41]. This suggests that nilotinib treatment can significantly impact the transcriptome, highlighting that the cellular response to nilotinib may not be uniform across patients.

Further investigations are warranted to explore the functional roles of these transporters and their potential contributions to drug resistance in CML.

To our knowledge, this study is the first to provide a comprehensive analysis of transcriptome profiling of BM CD34+/lin− cells from C-PCML patients at diagnosis, after 12 months of nilotinib treatment, and in comparison with healthy subjects. The distinct profiling highlights significant differences not only between the treated patients and their baseline (diagnosis) profiles but also in comparison to BM CD34+/lin− cells from CTRLs.

This underscores the complexity of the molecular landscape in C-PCML and the ongoing alterations that persist even after targeted therapy.

We could assume that nilotinib does not restore the BM CD34+/lin− cell population to a healthy transcriptomic state. More likely, however, nilotinib exerts selective pressure, shaping the leukemic cell population over time and potentially promoting its survival and self-renewal.

This aligns with the necessity for CML patients to receive continuous TKI treatment, primarily to prevent relapse, maintain disease control, suppress resistant clones, and achieve long-term remission.

A potential future direction would be to investigate whether the molecular signature of BM CD34+/lin− cells in CML patients treated for several years with TKIs other than nilotinib is similar or distinct.

Further analysis of these pathways could play a crucial role in refining treatment strategies and identifying potential therapeutic targets, ultimately improving patient outcomes in CML.

## Data Availability

The data presented in this study are openly available in the Gene Expression Omnibus repository at https://www.ncbi.nlm.nih.gov/geo/ with the ID GSE279135.

## Supplementary Materials

The following supporting information can be downloaded at www.mdpi.com/article/10.3390/cancers17061022/s1. Graphical plots S1: PCA and MvA plots; Figure S1: heatmaps of cell cycle; Figure S2: heatmaps of PI3K-Akt signaling pathway; Figure S3: heatmaps of MAPK signaling pathway; Figure S4: heatmaps of Ras signaling pathway; Table S1: DEGS among the three groups of subjects.
