# Supplementary material for: Bone Marrow CD34+/lin− Cells of Patients with Chronic-Phase Chronic Myeloid Leukemia (CP-CML) After 12 Months of Nilotinib Treatment Exhibit a Different Gene Expression Signature Compared to the Diagnosis and the Corresponding Cells from Healthy Subjects
Source: Cancers (Basel). 2025 Mar 18;17(6):1022. doi: 10.3390/cancers17061022 (PMC11940473; doi:10.3390/cancers17061022)
Supplement: Supplementary file 1 [file cancers-17-01022-s001.zip › Figure S1, S2, S3, S4 Heatmaps.pdf]

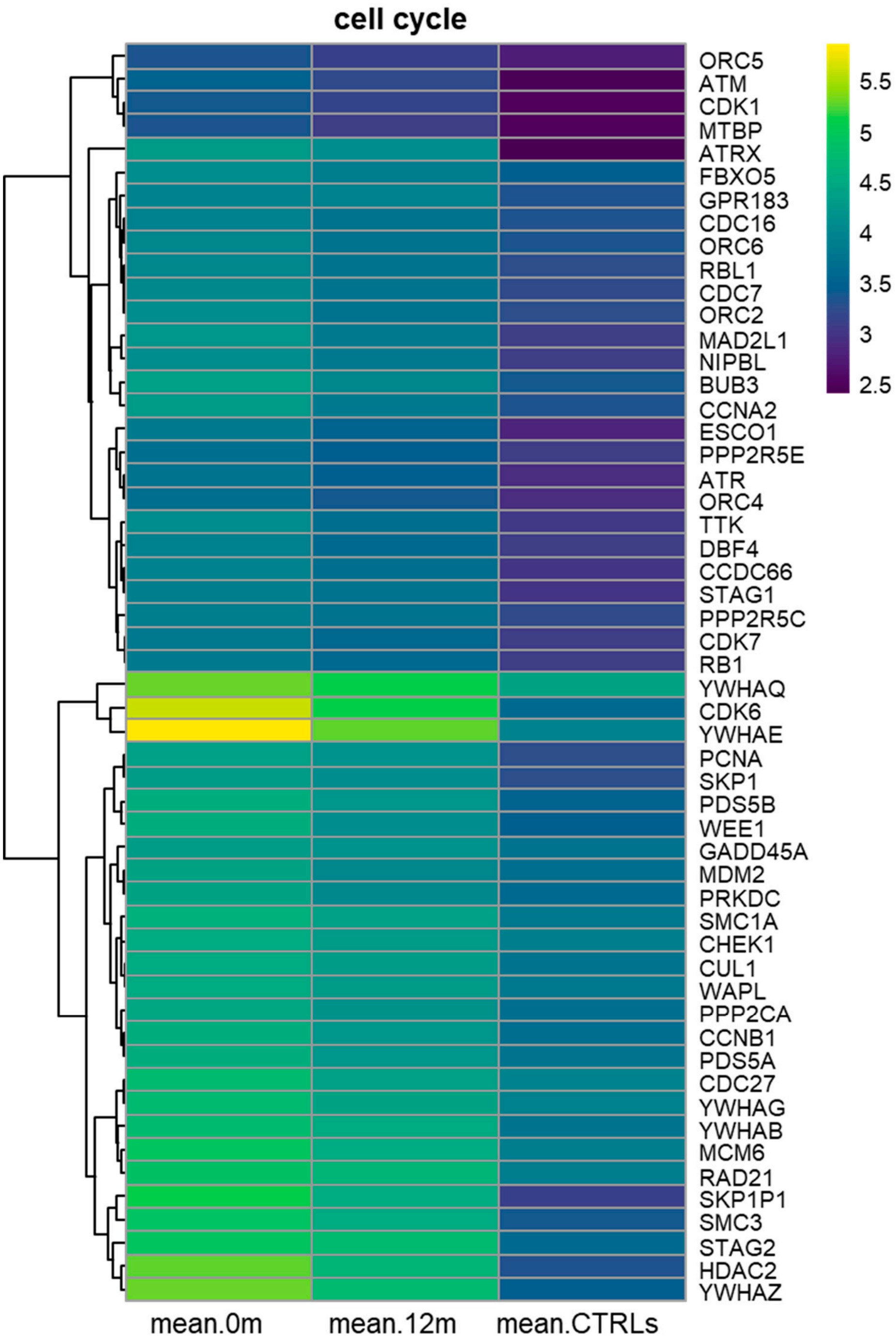

Figure S1. Heatmaps of Cell cycle. Heatmaps depicting the RMA-normalized expression levels (log2 scale) of DEGs in the cell cycle pathway at month 0, after 12 months of nilotinib, and in CTRL subjects. Hierarchical clustering using average linkage was applied to the genes to enhance visualization.

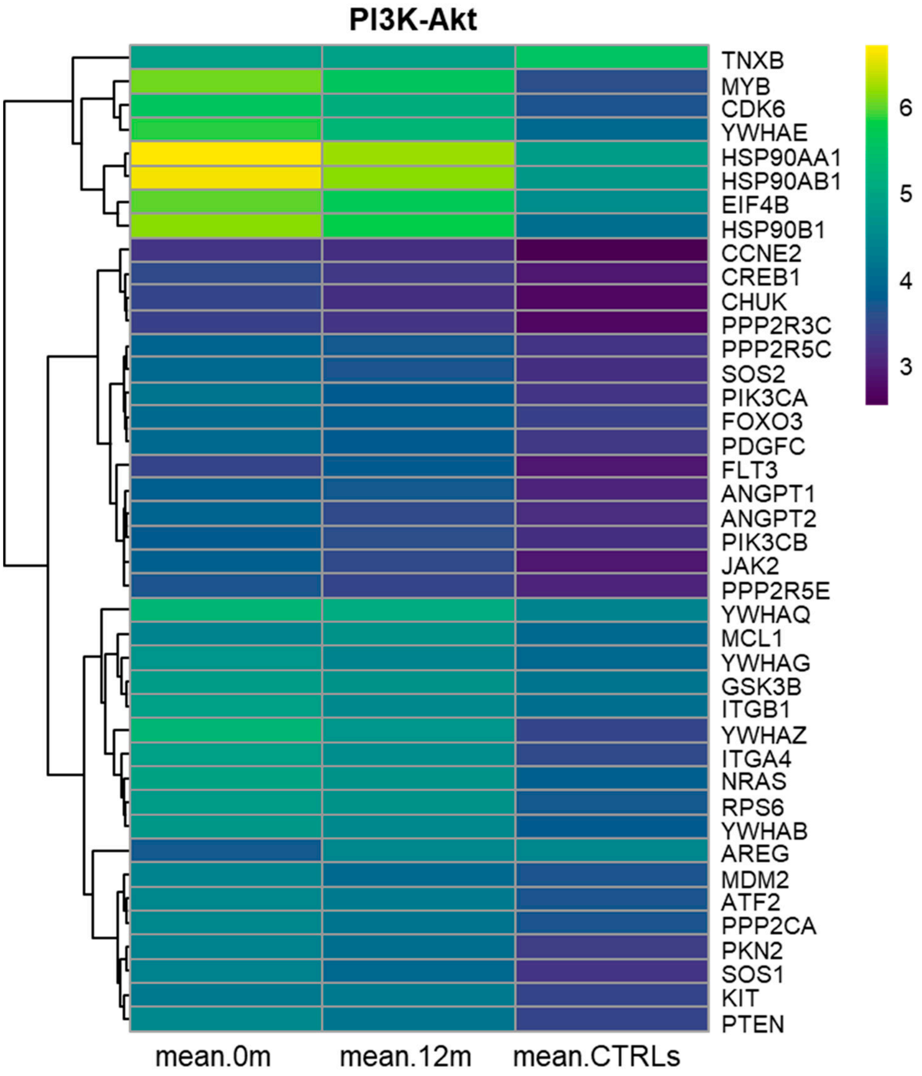

Figure S2. Heatmaps of PI3k-Akt Signaling Pathway. Heatmaps depicting the RMA-normalized expression levels (log2 scale) of DEGs in the PI3K-Akt pathway at month 0, after 12 months of nilotinib, and in CTRL subjects. Hierarchical clustering using average linkage was applied to the genes to enhance visualization.

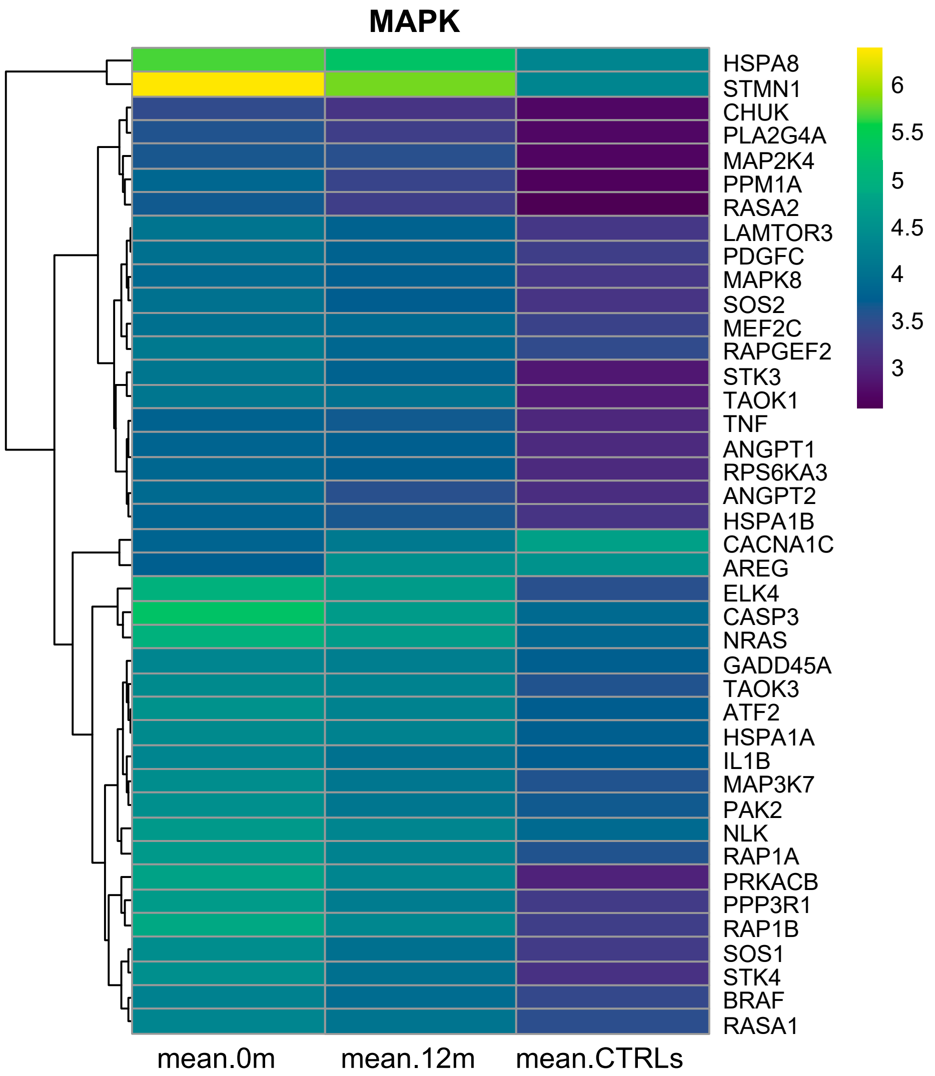

Figure S3. Heatmaps of MAPK Signaling Pathway. Heatmaps depicting the RMA-normalized expression levels (log2 scale) of DEGs in the MAPK pathway at month 0, after 12 months of nilotinib, and in CTRL subjects. Hierarchical clustering using average linkage was applied to the genes to enhance visualization

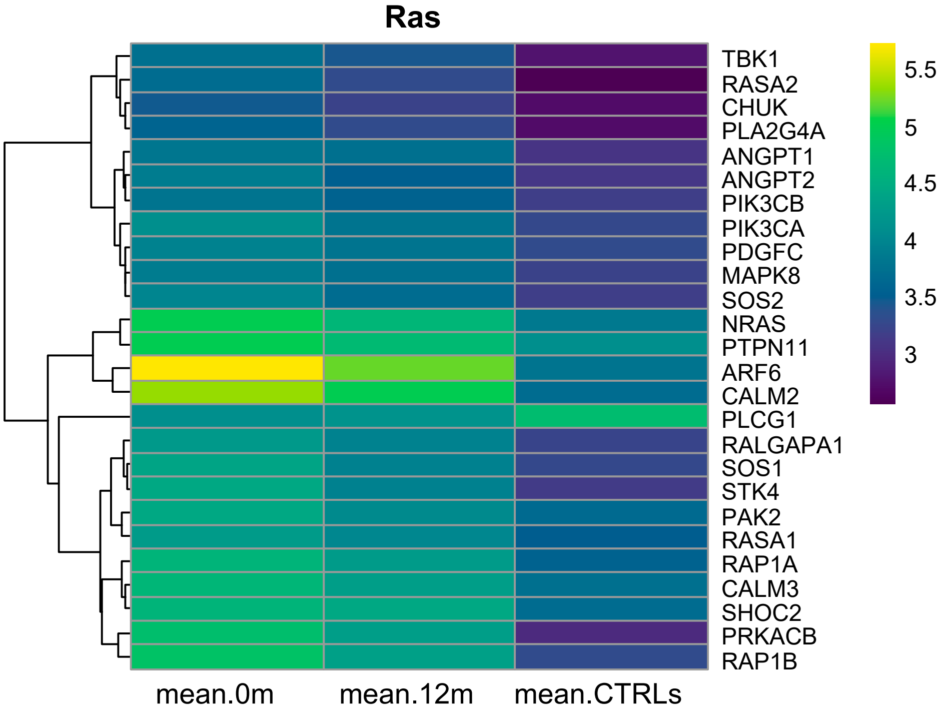

Figure S4. Heatmaps of Ras Signaling Pathway. Heatmaps depicting the RMA-normalized expression levels (log2 scale) of DEGs in the Ras pathway at month 0, after 12 months of nilotinib, and in CTRL subjects. Hierarchical clustering using average linkage was applied to the genes to enhance visualization
