# Supplementary material for: Bone Marrow CD34+/lin− Cells of Patients with Chronic-Phase Chronic Myeloid Leukemia (CP-CML) After 12 Months of Nilotinib Treatment Exhibit a Different Gene Expression Signature Compared to the Diagnosis and the Corresponding Cells from Healthy Subjects
Source: Cancers (Basel). 2025 Mar 18;17(6):1022. doi: 10.3390/cancers17061022 (PMC11940473; doi:10.3390/cancers17061022)

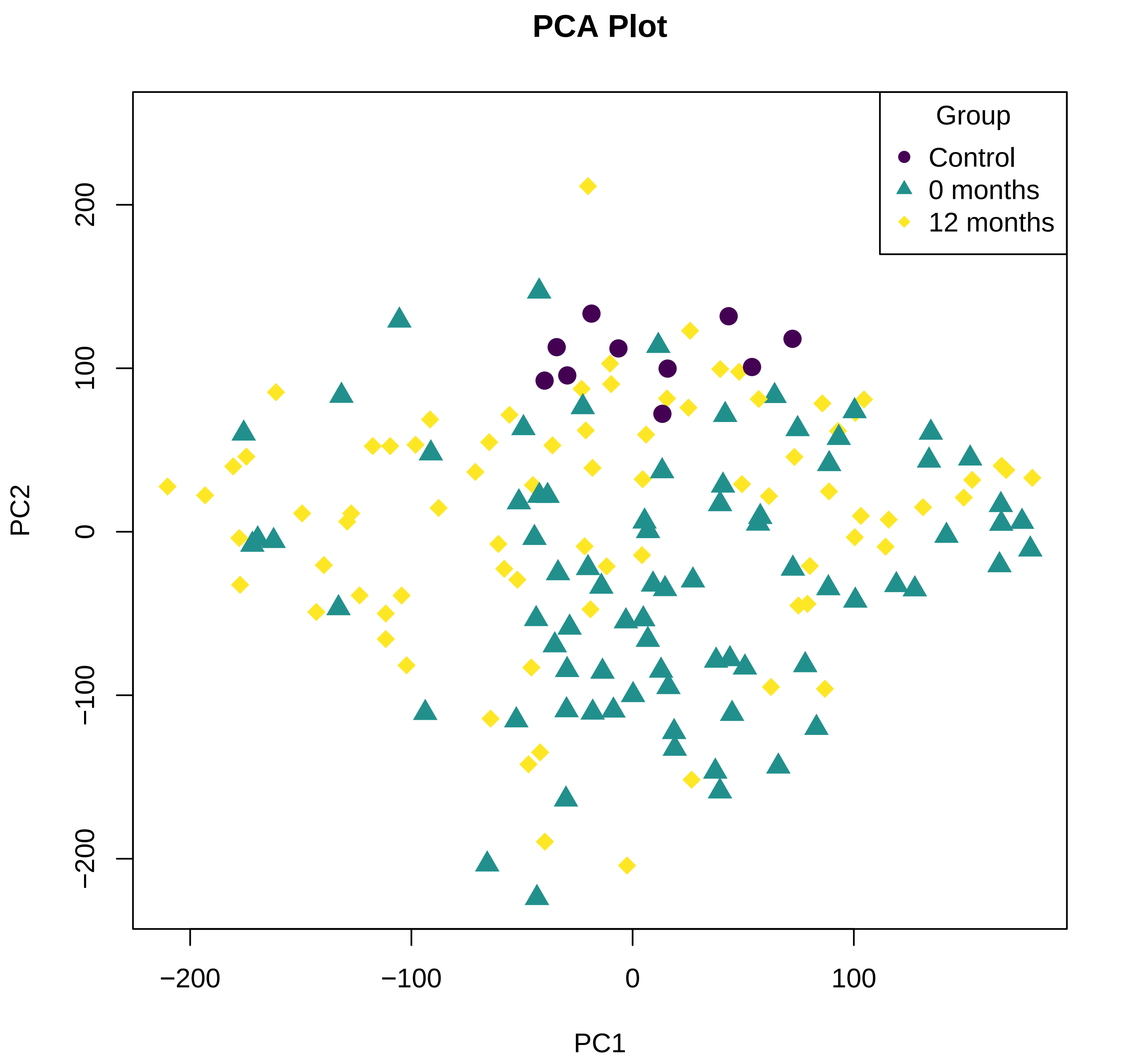
PCA Plot
Group
Control
0 months
12 months
PC1
PC2
−200
−100
0
100
200
−200
−100
0
100

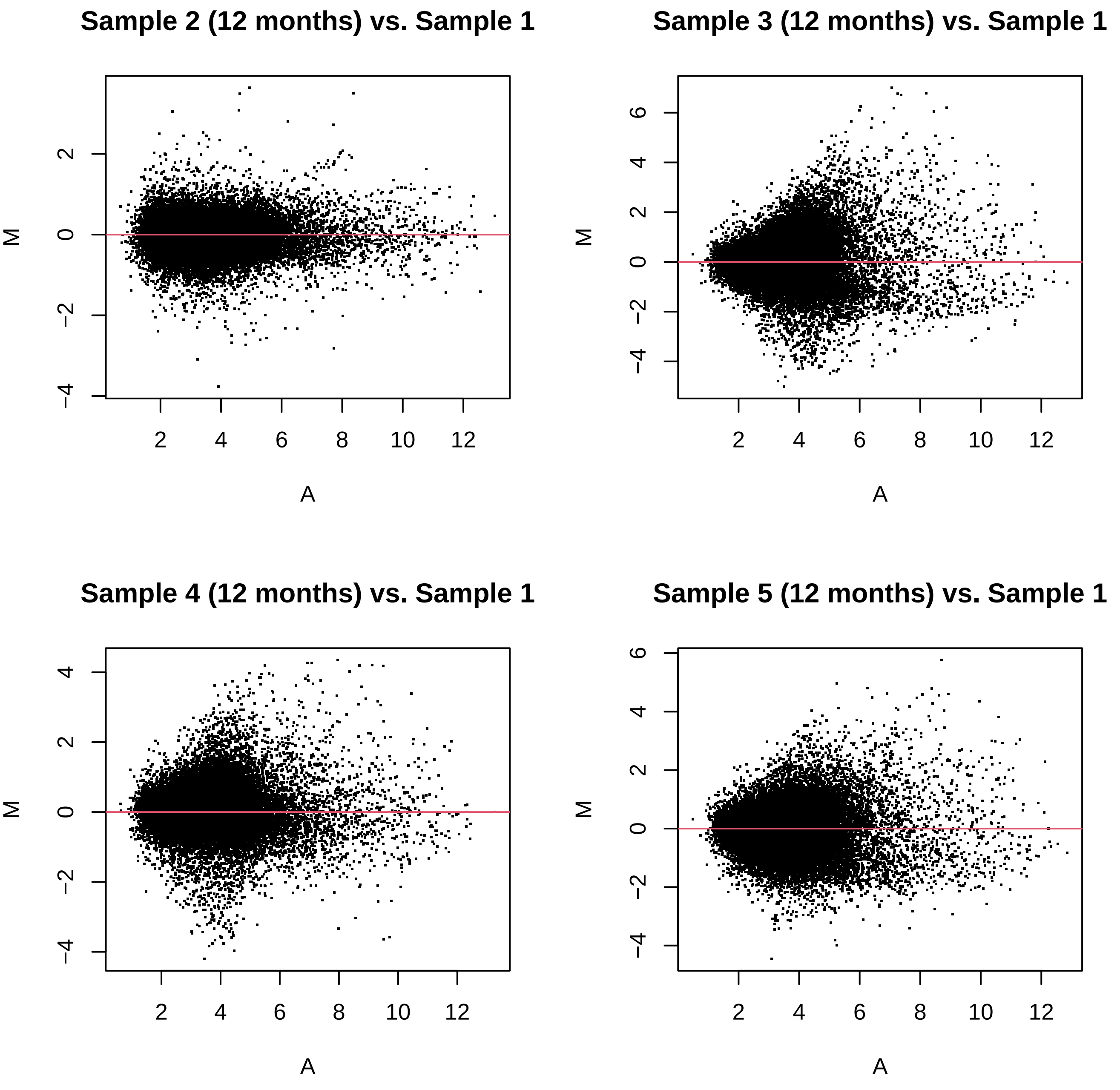
Sample 2 (12 months) vs. Sample 1
Sample 3 (12 months) vs. Sample 1
Sample 4 (12 months) vs. Sample 1
Sample 5 (12 months) vs. Sample 1
M
A
2
4
6
8
10
12
−4
−2
0
2
4
6

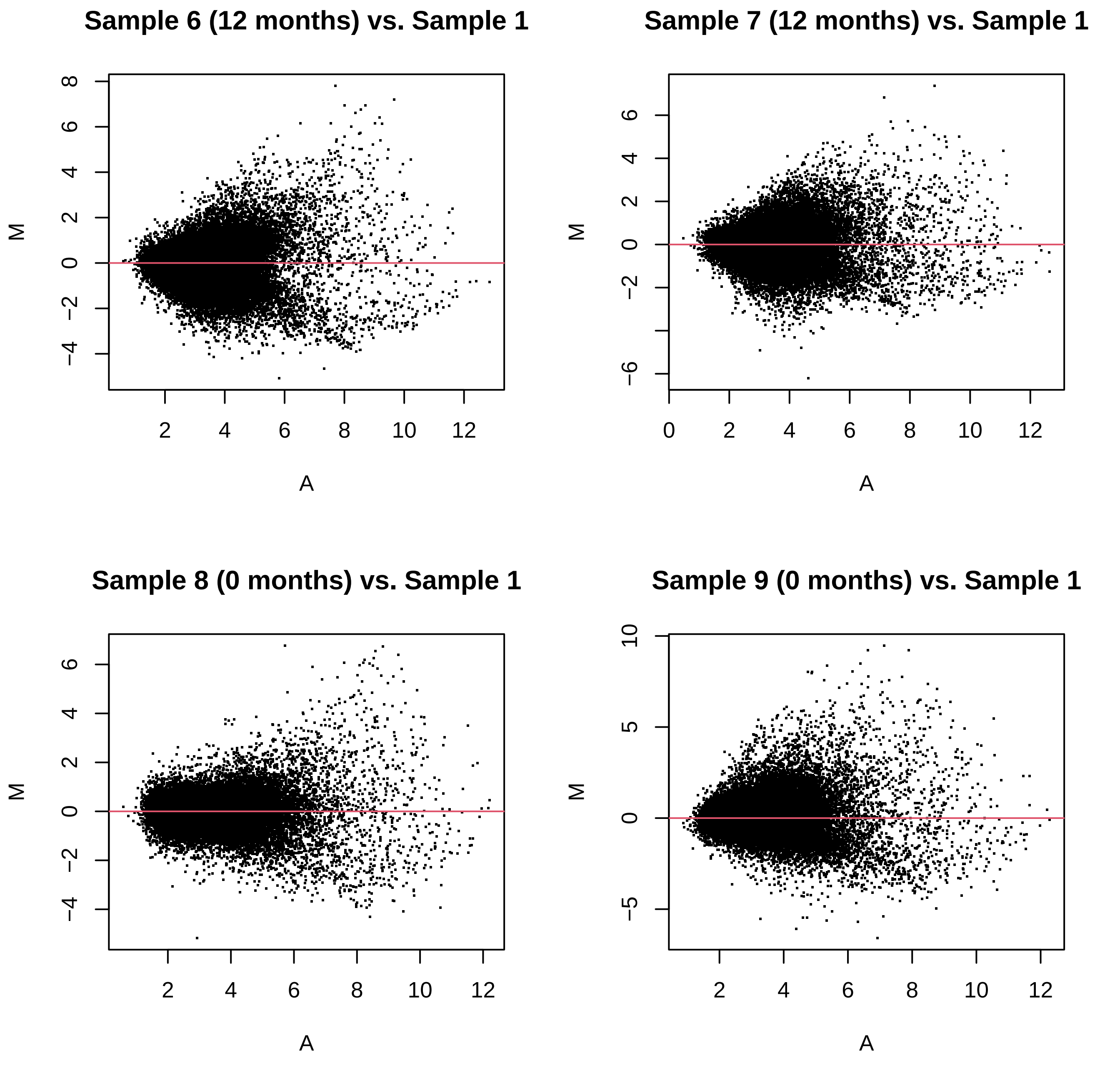

Sample 6 (12 months) vs. Sample 1
Sample 7 (12 months) vs. Sample 1
Sample 8 (0 months) vs. Sample 1
Sample 9 (0 months) vs. Sample 1
M
A

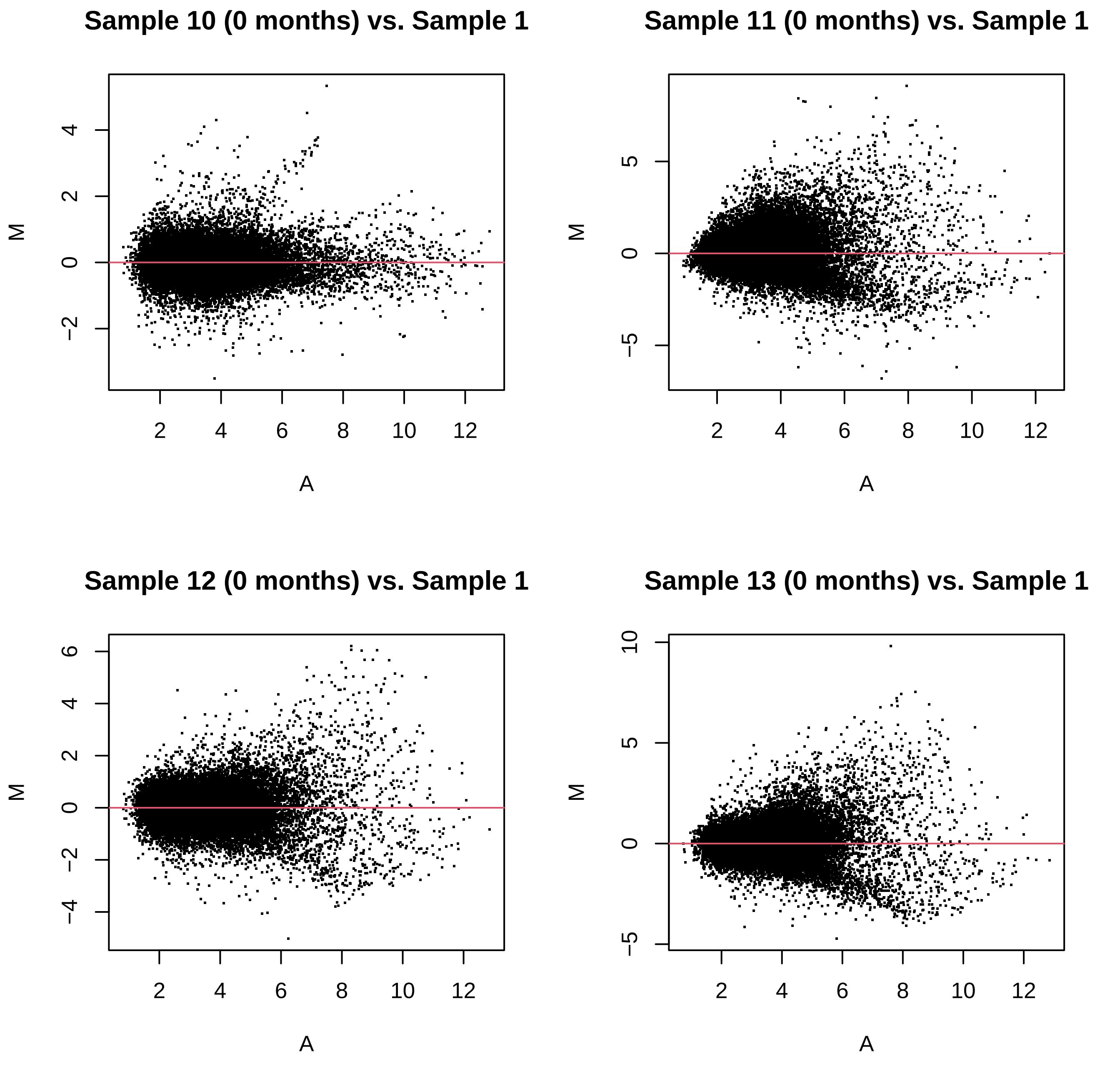
Sample 10 (0 months) vs. Sample 1
M
A
Sample 11 (0 months) vs. Sample 1
M
A
Sample 12 (0 months) vs. Sample 1
M
A
Sample 13 (0 months) vs. Sample 1
M
A

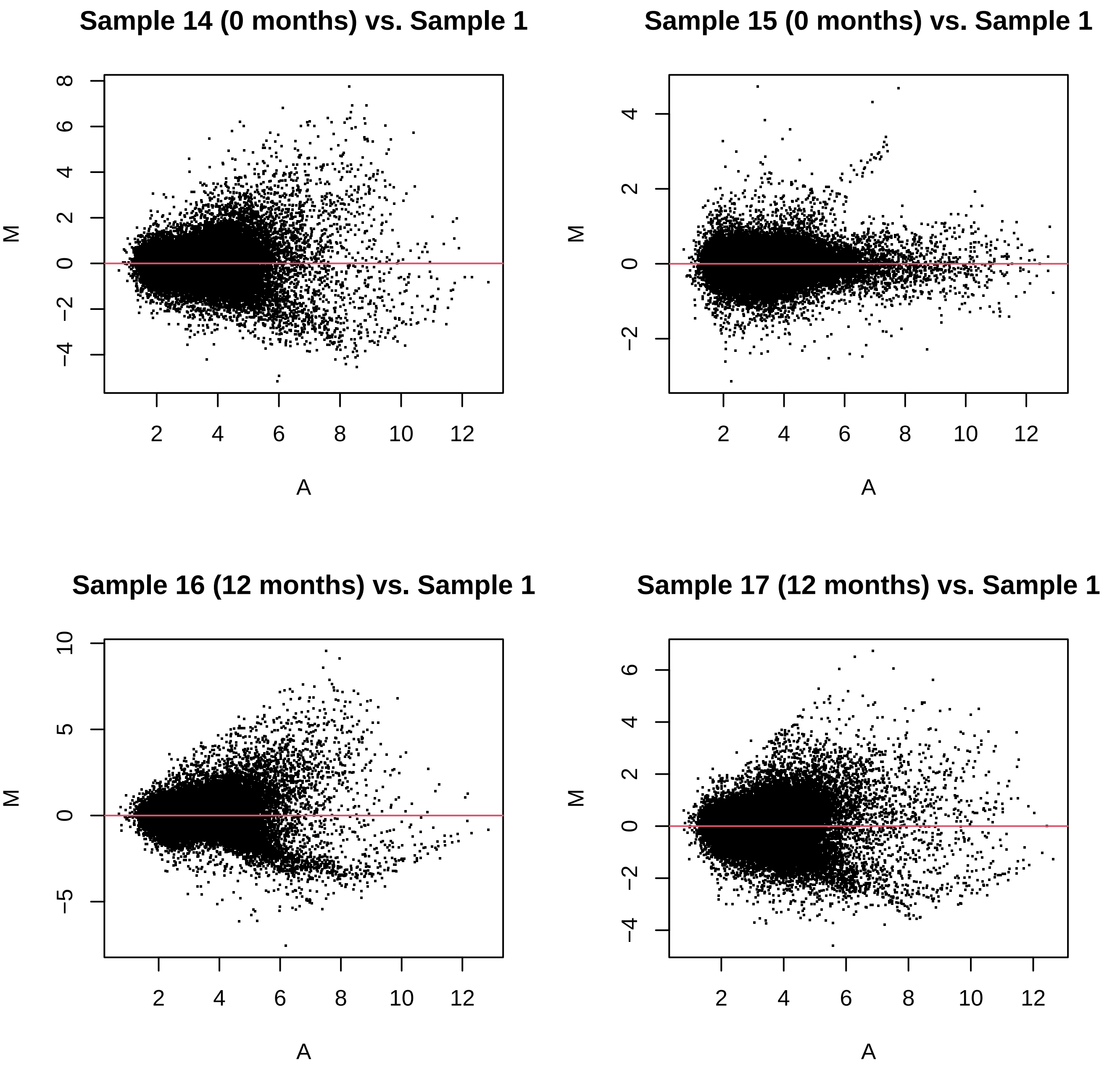
Sample 14 (0 months) vs. Sample 1
Sample 15 (0 months) vs. Sample 1
Sample 16 (12 months) vs. Sample 1
Sample 17 (12 months) vs. Sample 1
M
A

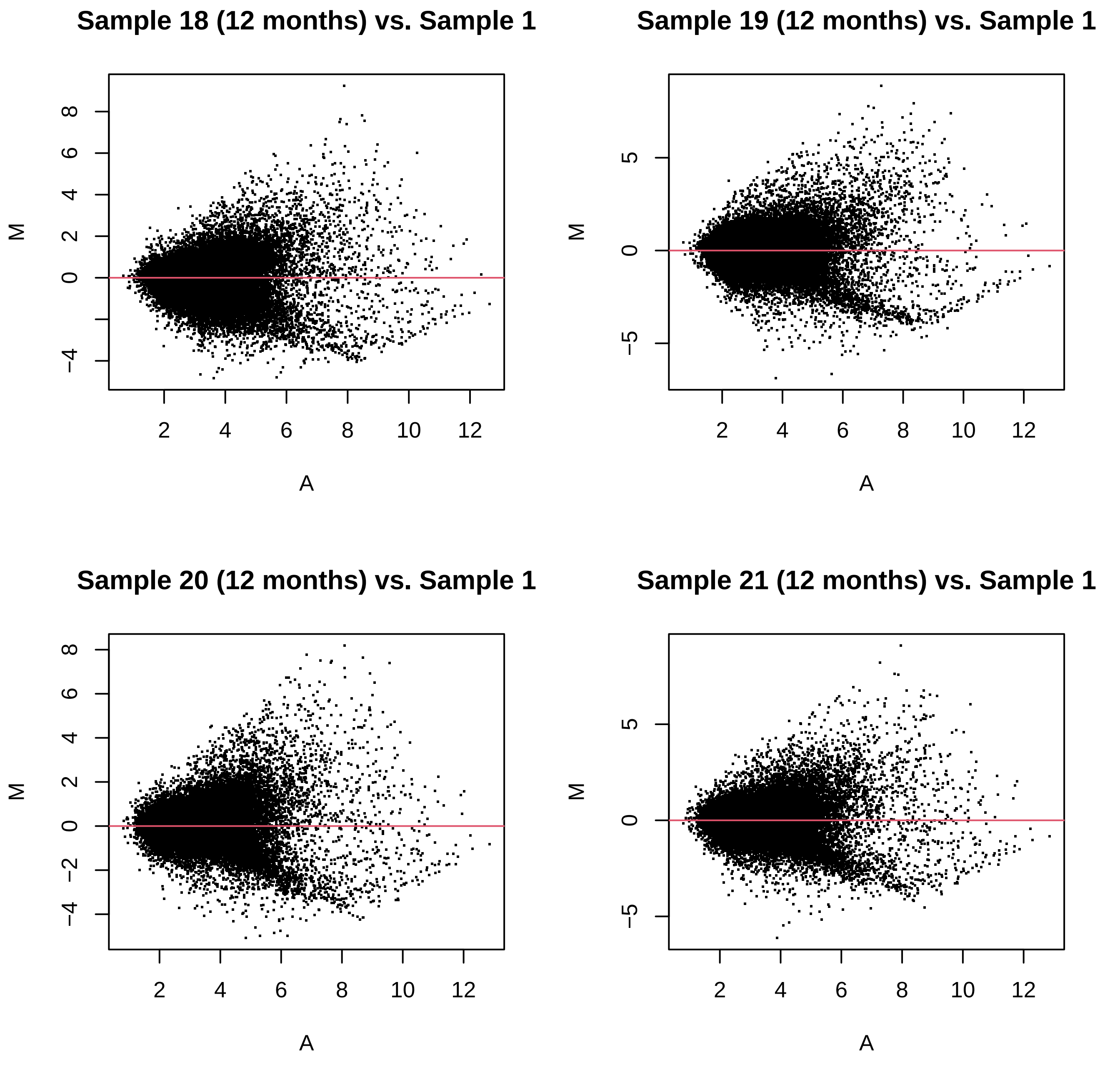
Sample 18 (12 months) vs. Sample 1
M
A
Sample 19 (12 months) vs. Sample 1
M
A
Sample 20 (12 months) vs. Sample 1
M
A
Sample 21 (12 months) vs. Sample 1
M
A

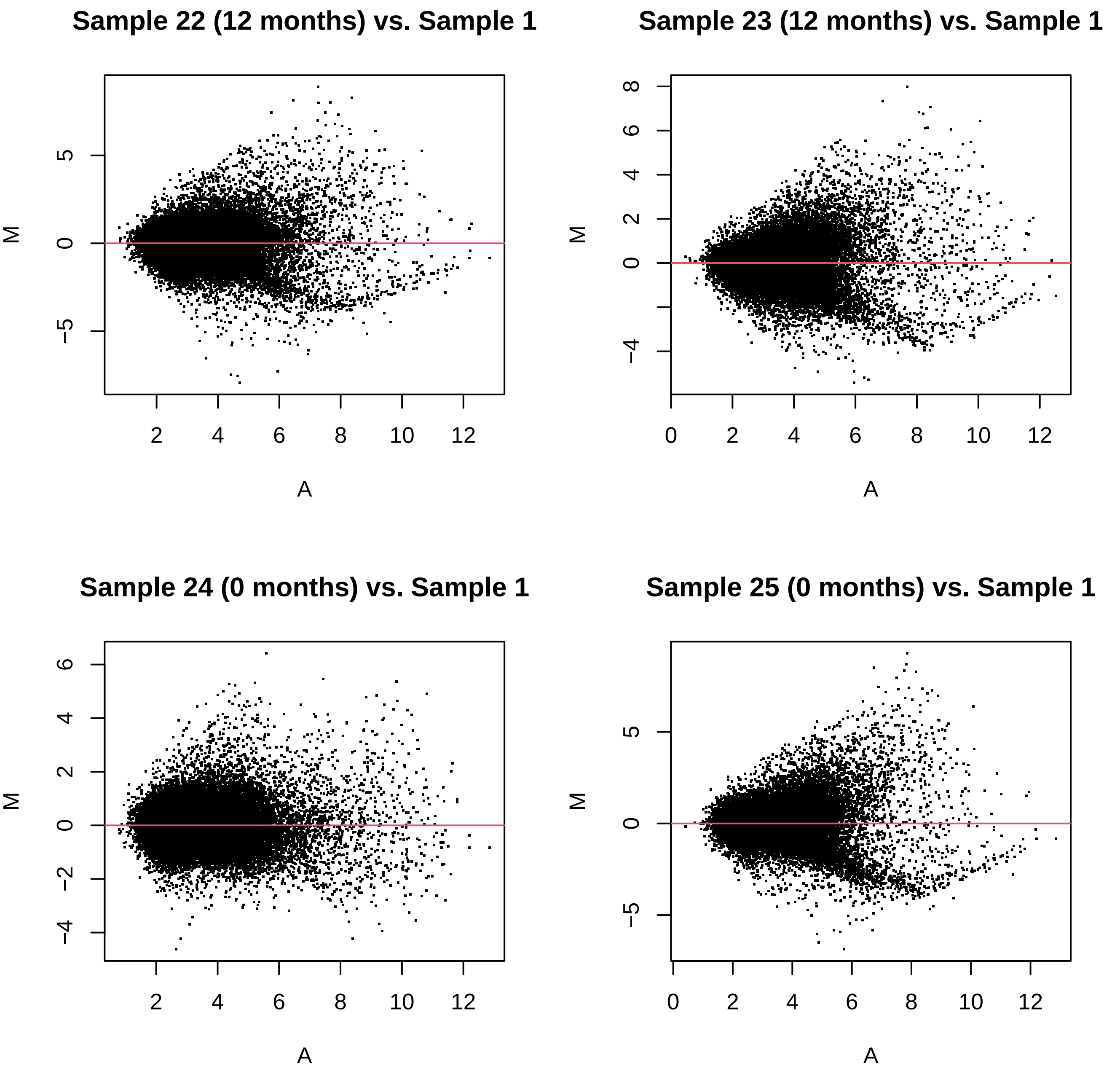

Sample 22 (12 months) vs. Sample 1
Sample 23 (12 months) vs. Sample 1
Sample 24 (0 months) vs. Sample 1
Sample 25 (0 months) vs. Sample 1
M
A

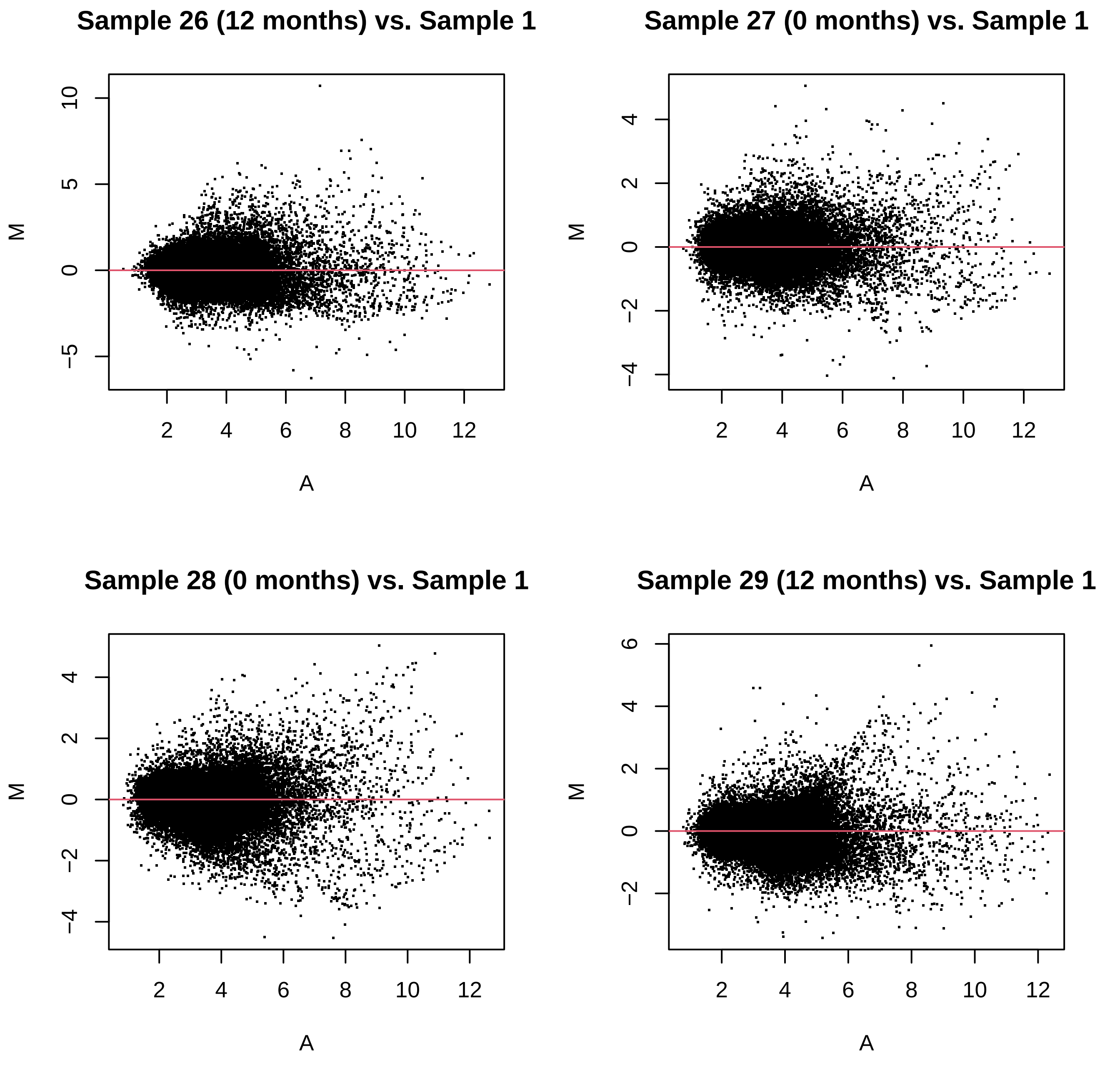
Sample 26 (12 months) vs. Sample 1
Sample 27 (0 months) vs. Sample 1
Sample 28 (0 months) vs. Sample 1
Sample 29 (12 months) vs. Sample 1
M
A

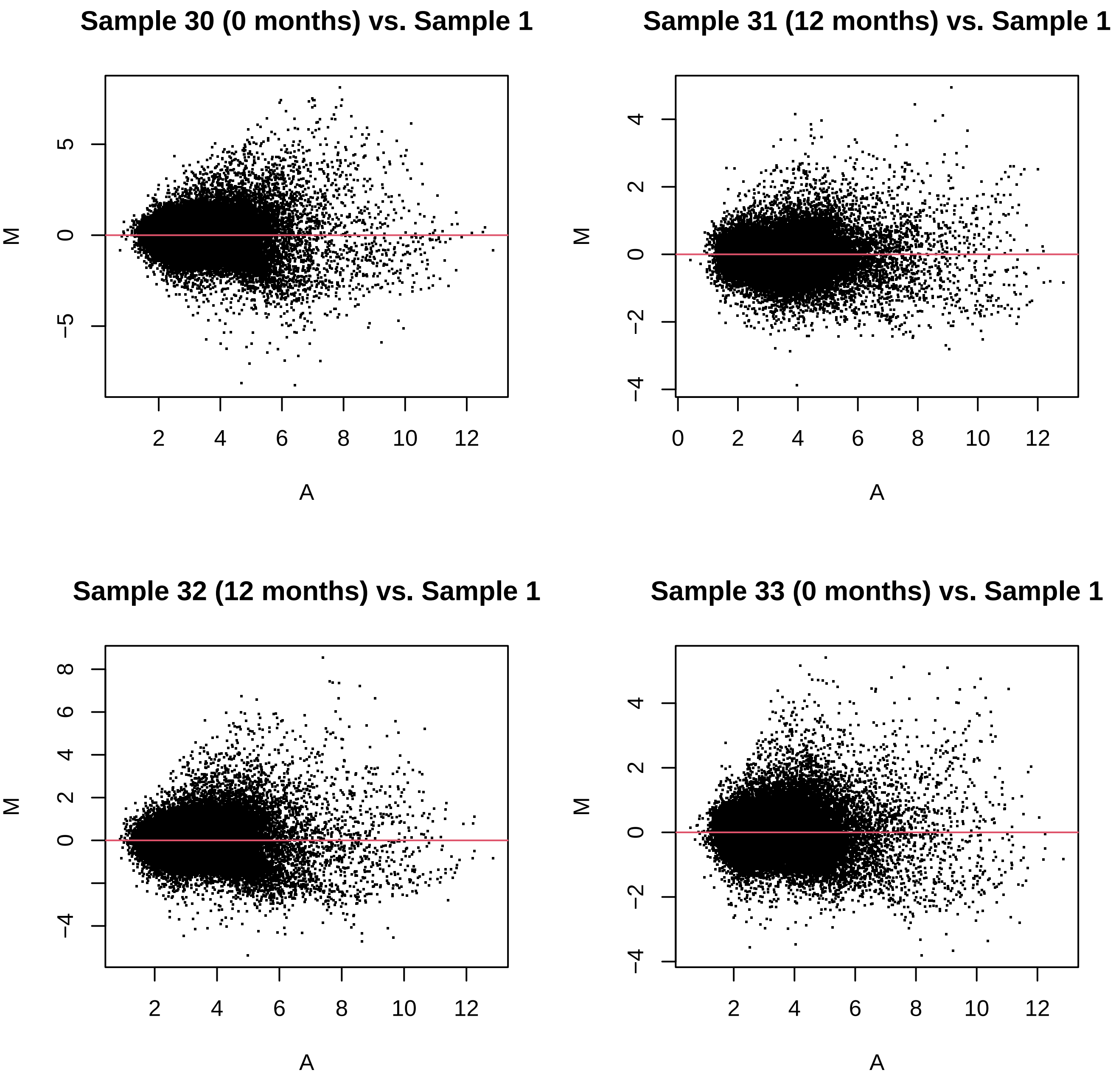
Sample 30 (0 months) vs. Sample 1
M
A
Sample 31 (12 months) vs. Sample 1
M
A
Sample 32 (12 months) vs. Sample 1
M
A
Sample 33 (0 months) vs. Sample 1
M
A

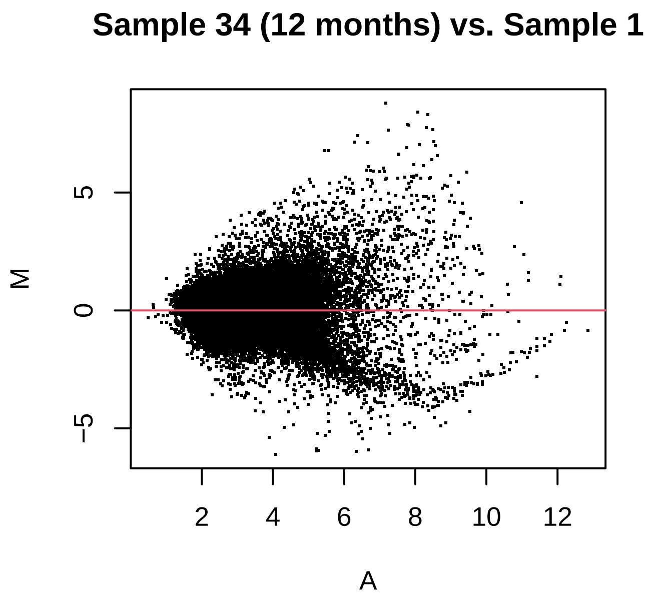
Sample 34 (12 months) vs. Sample 1
M
A
5
0
−5
2
4
6
8
10
12

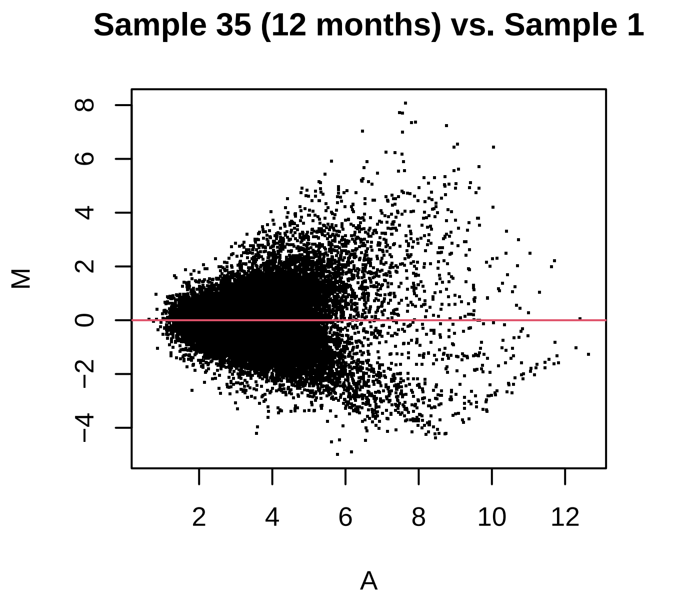
Sample 35 (12 months) vs. Sample 1
M
A
8
6
4
2
0
−2
−4
2
4
6
8
10
12

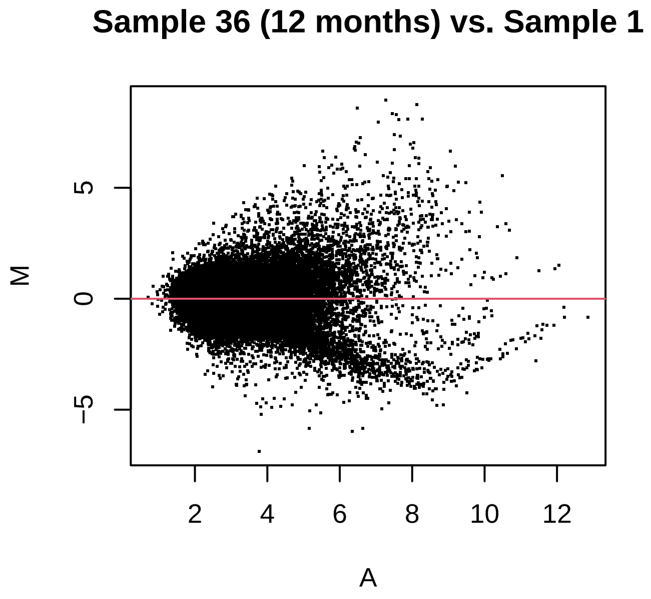
Sample 36 (12 months) vs. Sample 1
M
A
5
0
−5
2
4
6
8
10
12

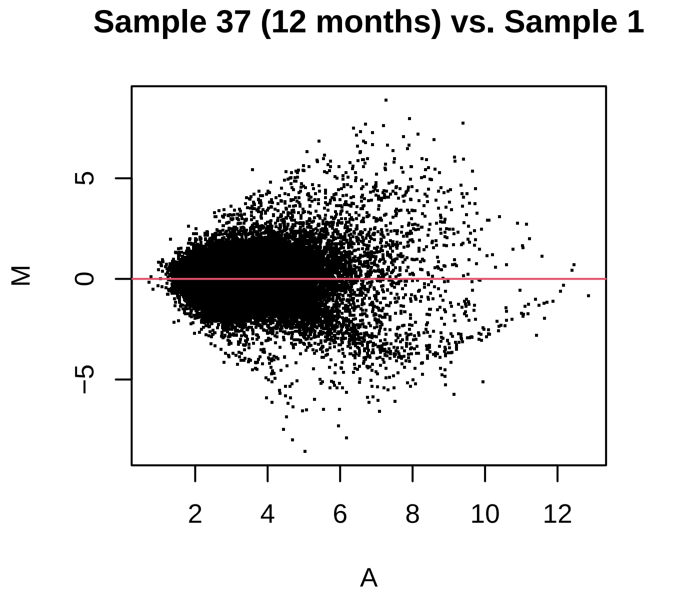
Sample 37 (12 months) vs. Sample 1
M
A
5
0
−5
2
4
6
8
10
12

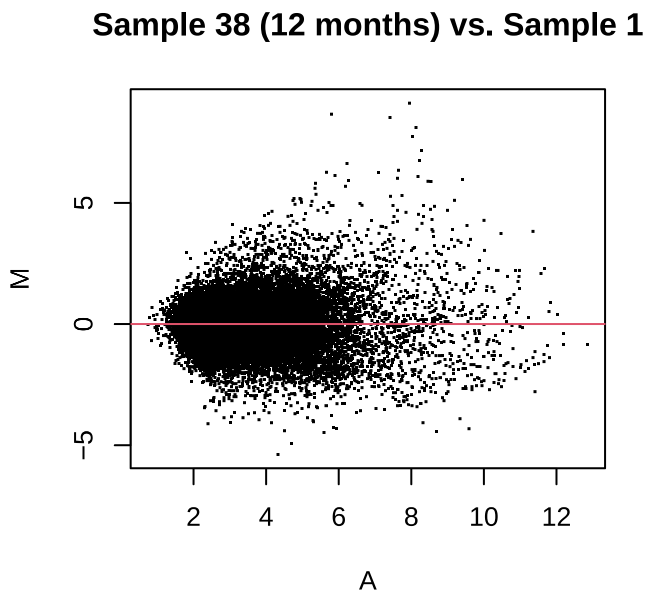
Sample 38 (12 months) vs. Sample 1
M
A
−5
0
5
2
4
6
8
10
12

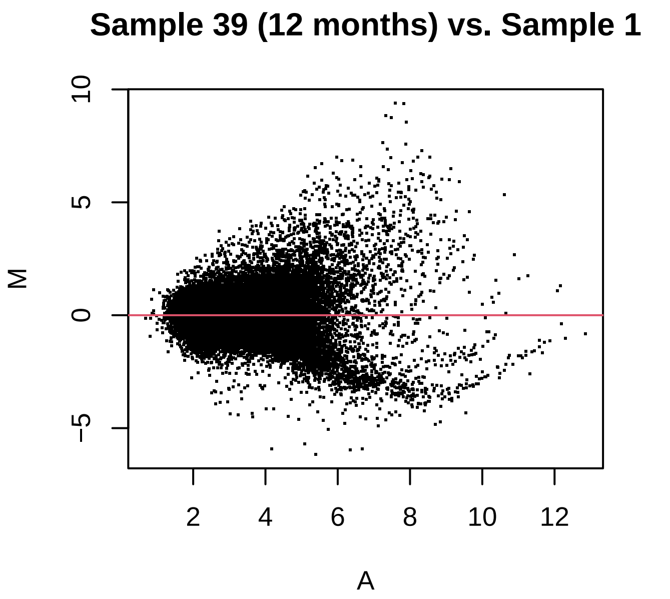
Sample 39 (12 months) vs. Sample 1
M
A
−5
0
5
10
2
4
6
8
10
12

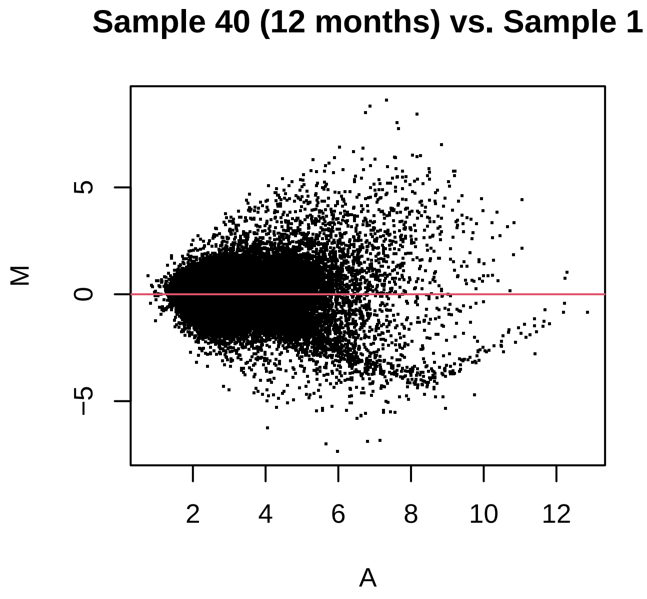
Sample 40 (12 months) vs. Sample 1
M
A
−5
0
5
2
4
6
8
10
12

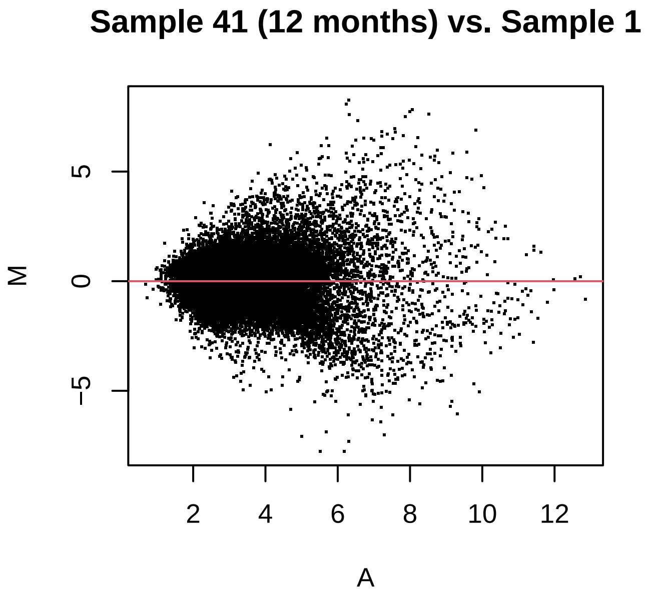
Sample 41 (12 months) vs. Sample 1
M
A
−5
0
5
2
4
6
8
10
12

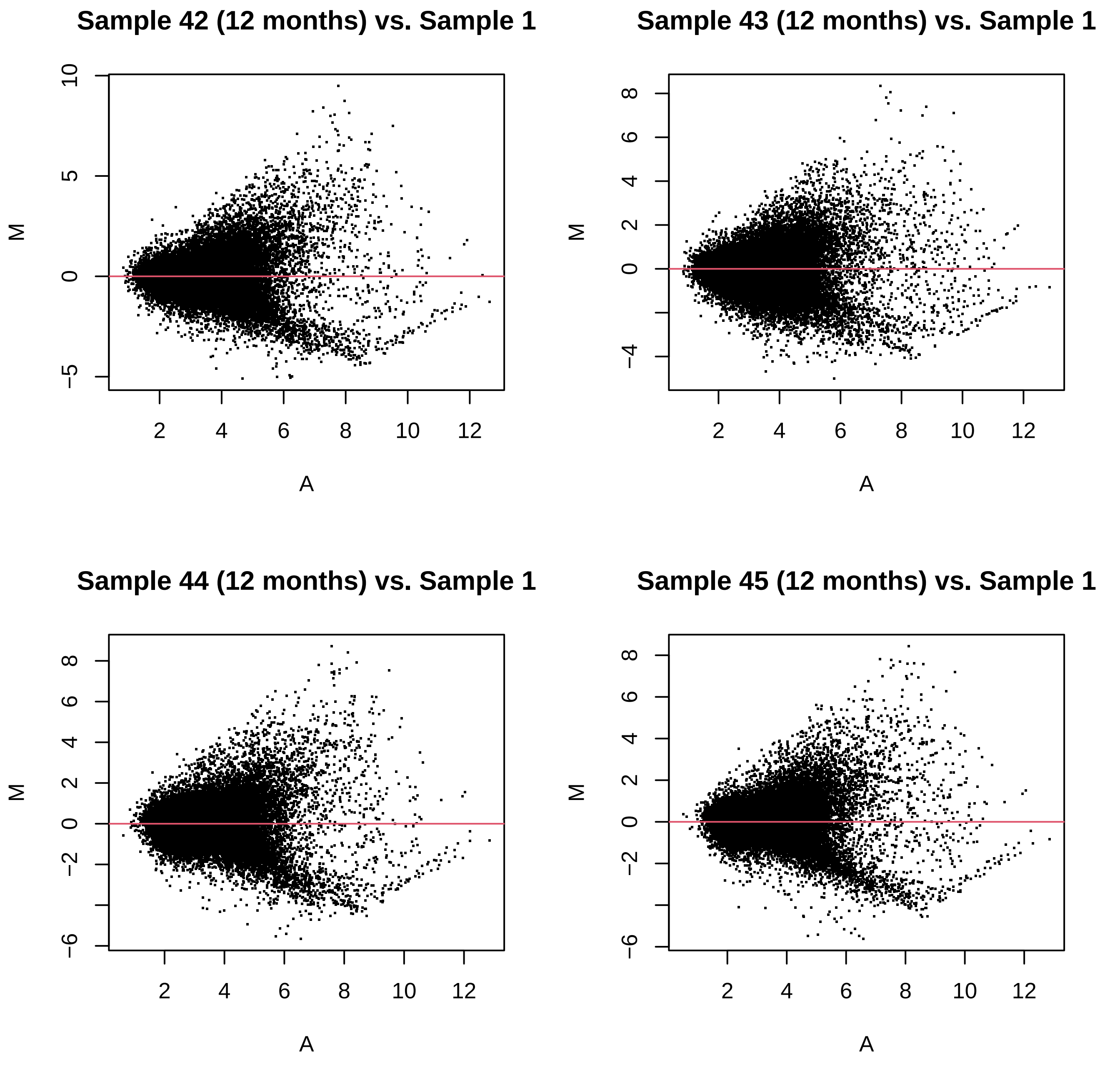

Sample 42 (12 months) vs. Sample 1
Sample 43 (12 months) vs. Sample 1
Sample 44 (12 months) vs. Sample 1
Sample 45 (12 months) vs. Sample 1
M
A

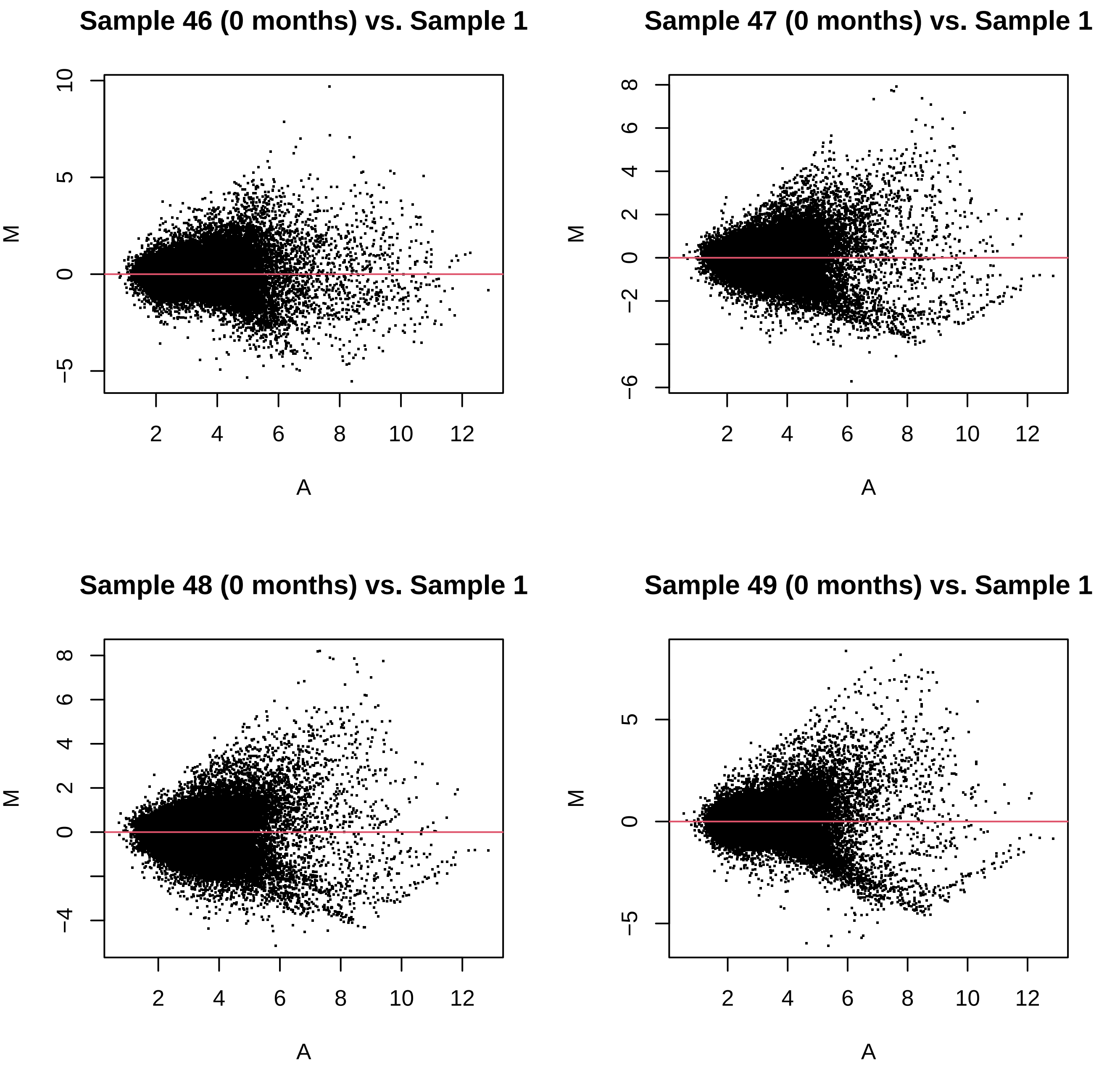
Sample 46 (0 months) vs. Sample 1
Sample 47 (0 months) vs. Sample 1
Sample 48 (0 months) vs. Sample 1
Sample 49 (0 months) vs. Sample 1
M
A

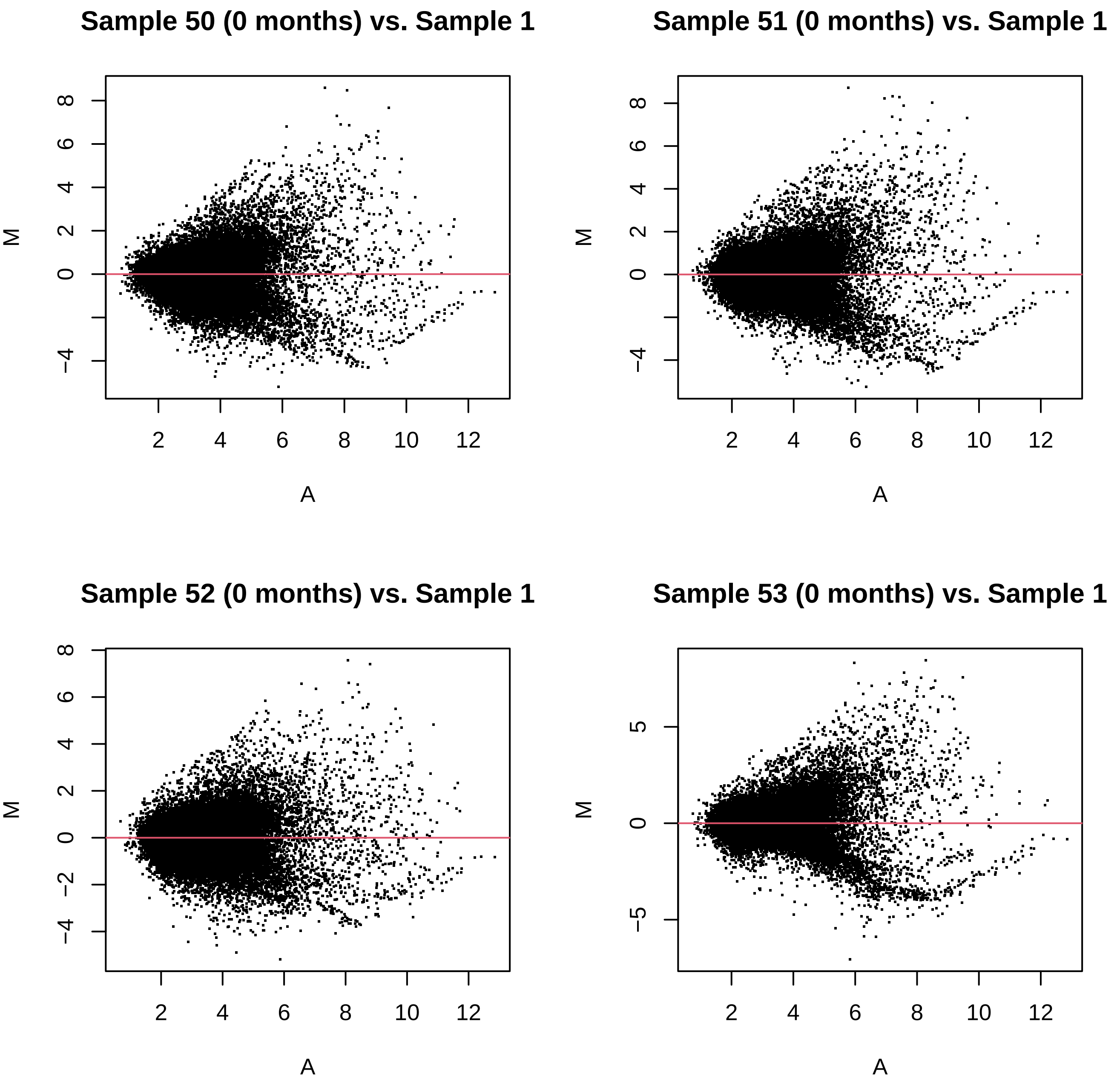
Sample 50 (0 months) vs. Sample 1
M
A
Sample 51 (0 months) vs. Sample 1
M
A
Sample 52 (0 months) vs. Sample 1
M
A
Sample 53 (0 months) vs. Sample 1
M
A

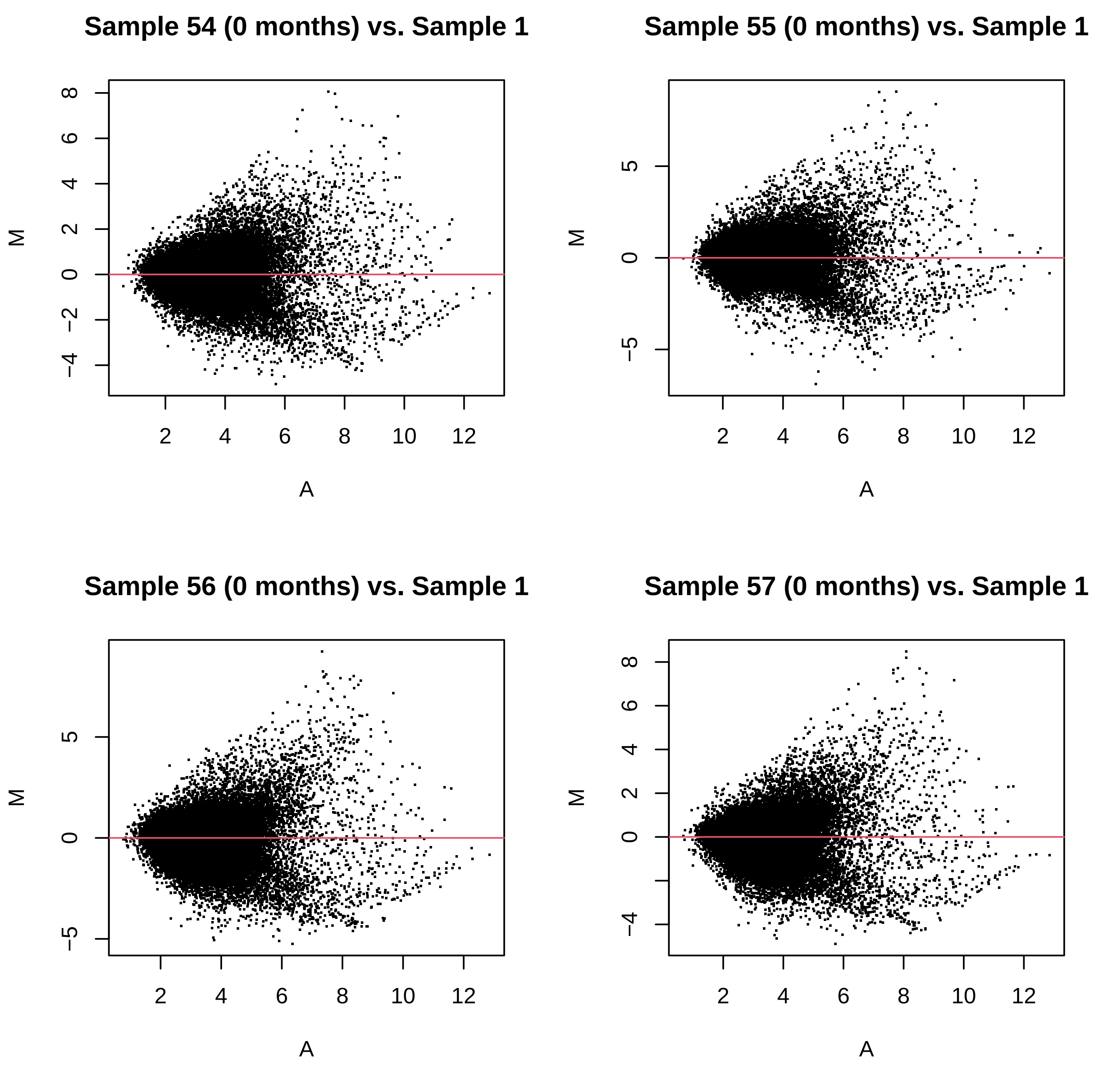
Sample 54 (0 months) vs. Sample 1
M
A
8
6
4
2
0
−2
−4
2
4
6
8
10
12
Sample 55 (0 months) vs. Sample 1
M
A
5
0
−5
2
4
6
8
10
12
Sample 56 (0 months) vs. Sample 1
M
A
5
0
−5
2
4
6
8
10
12
Sample 57 (0 months) vs. Sample 1
M
A
8
6
4
2
0
−4
2
4
6
8
10
12

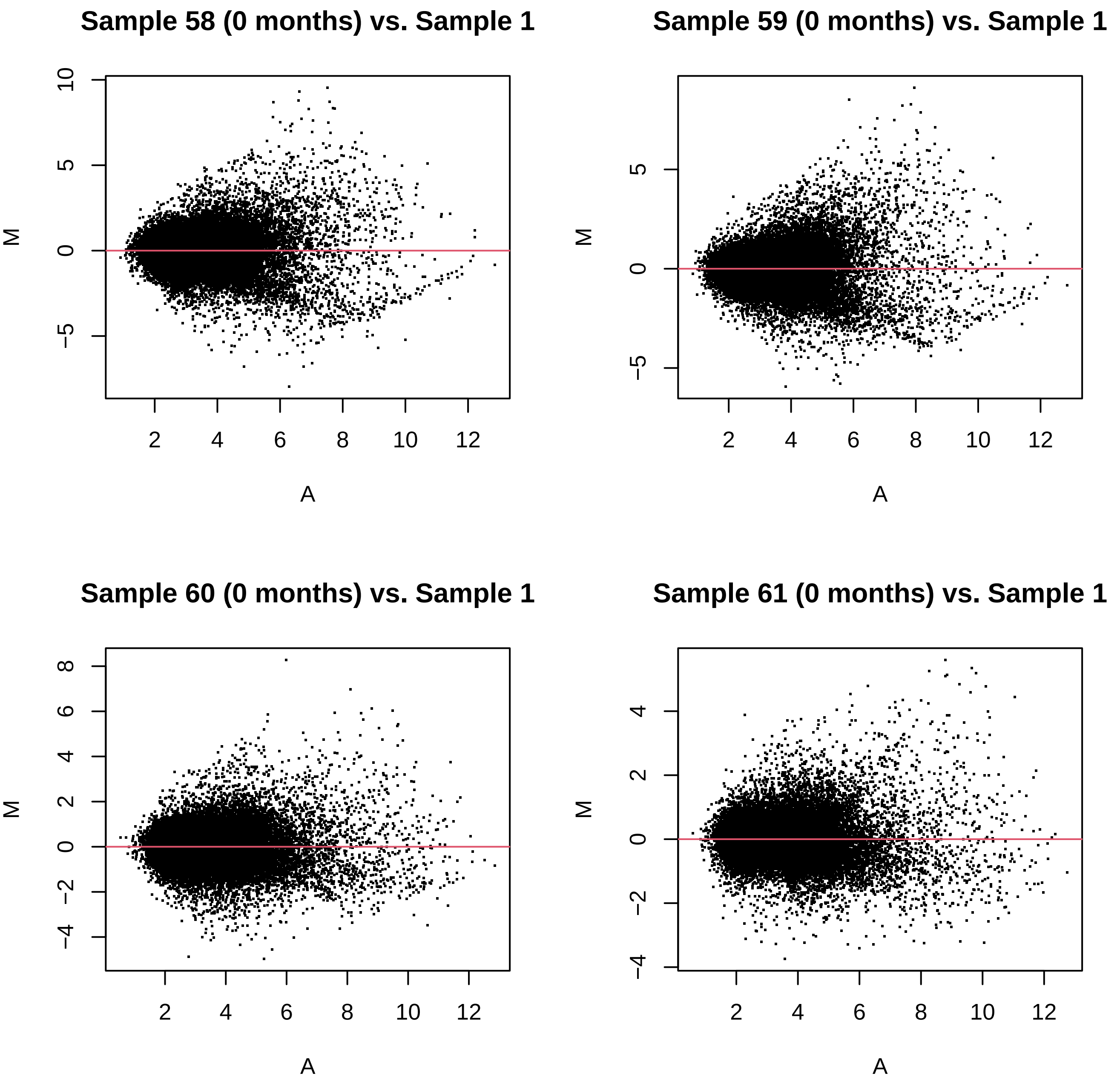
Sample 58 (0 months) vs. Sample 1
Sample 59 (0 months) vs. Sample 1
Sample 60 (0 months) vs. Sample 1
Sample 61 (0 months) vs. Sample 1
M
A

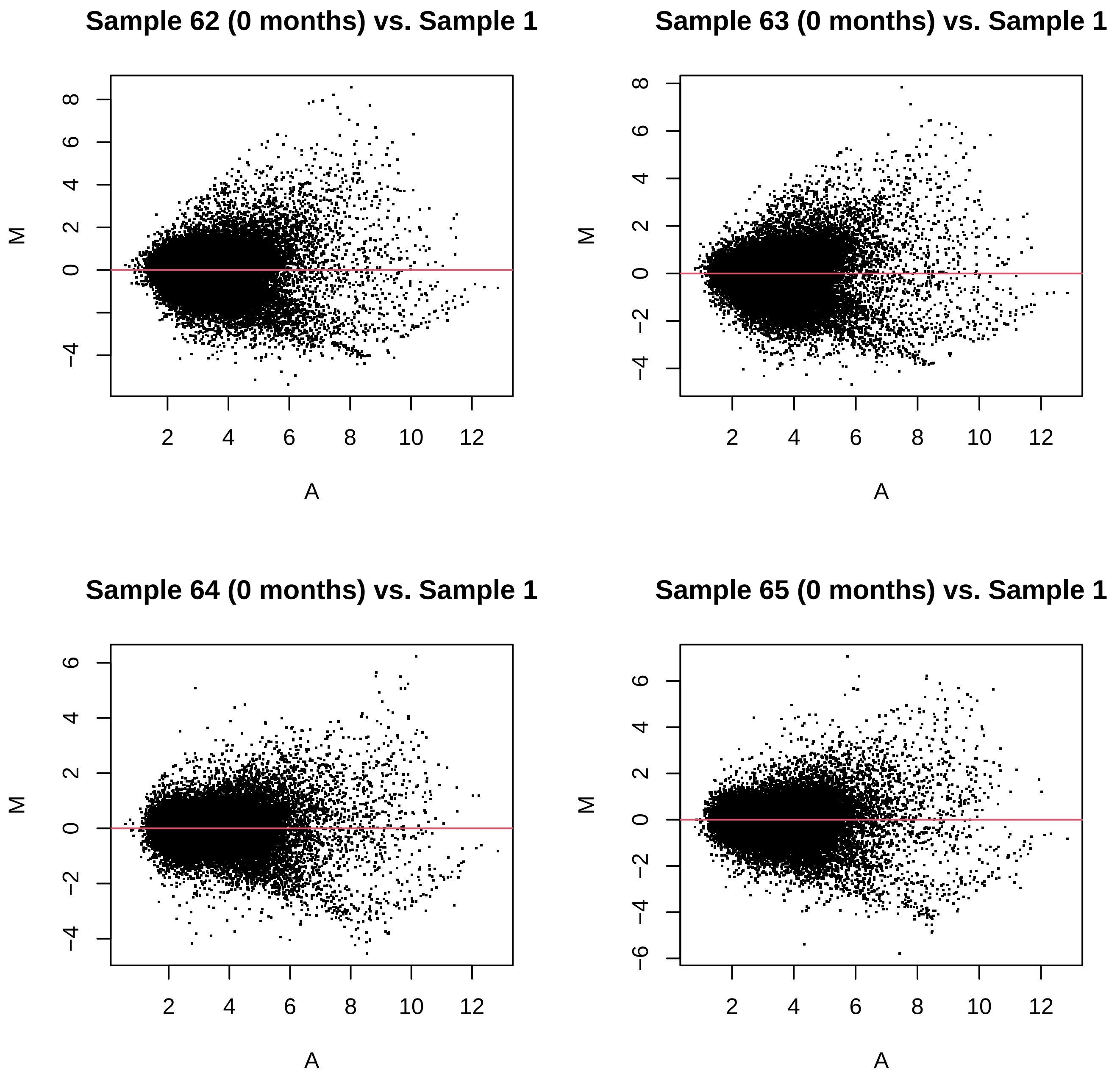
Sample 62 (0 months) vs. Sample 1
M
A
−4
0
2
4
6
8
2
4
6
8
10
12
Sample 63 (0 months) vs. Sample 1
M
A
−4
−2
0
2
4
6
8
2
4
6
8
10
12
Sample 64 (0 months) vs. Sample 1
M
A
−4
−2
0
2
4
6
2
4
6
8
10
12
Sample 65 (0 months) vs. Sample 1
M
A
−6
−4
−2
0
2
4
6
2
4
6
8
10
12

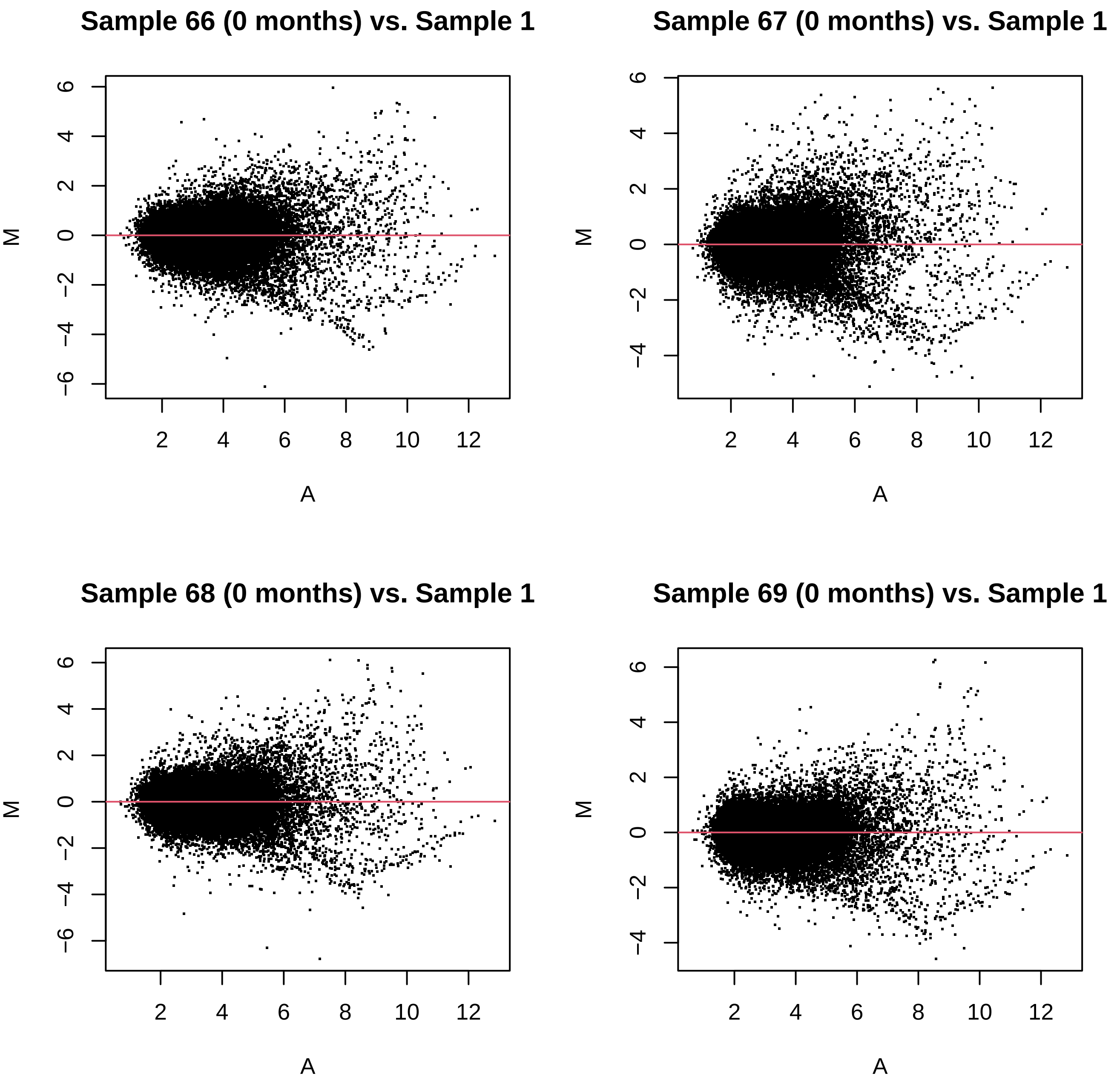
Sample 66 (0 months) vs. Sample 1
Sample 67 (0 months) vs. Sample 1
Sample 68 (0 months) vs. Sample 1
Sample 69 (0 months) vs. Sample 1
M
A

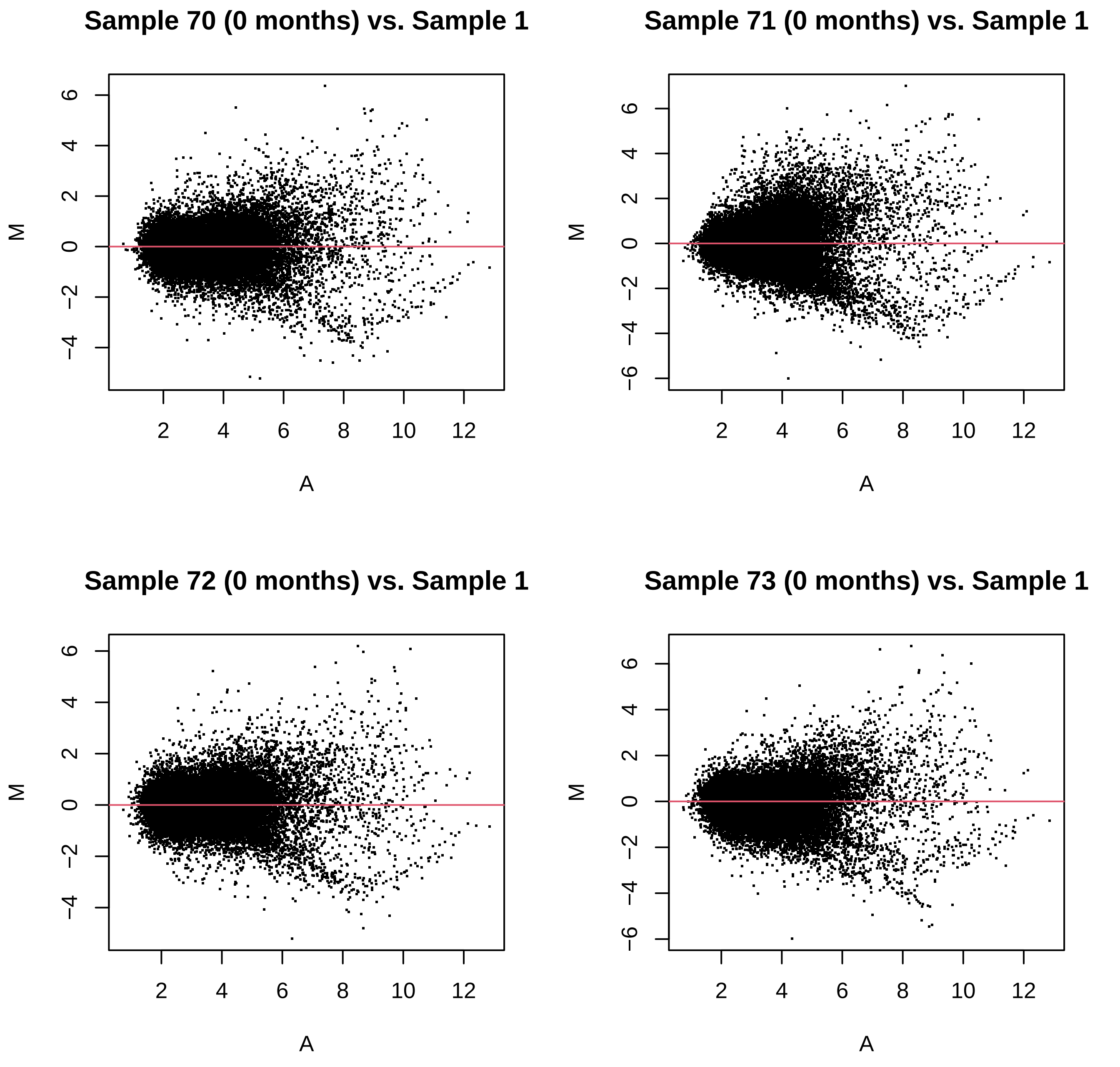
Sample 70 (0 months) vs. Sample 1
Sample 71 (0 months) vs. Sample 1
Sample 72 (0 months) vs. Sample 1
Sample 73 (0 months) vs. Sample 1
M
A
−6
−4
−2
0
2
4
6
8
10
12

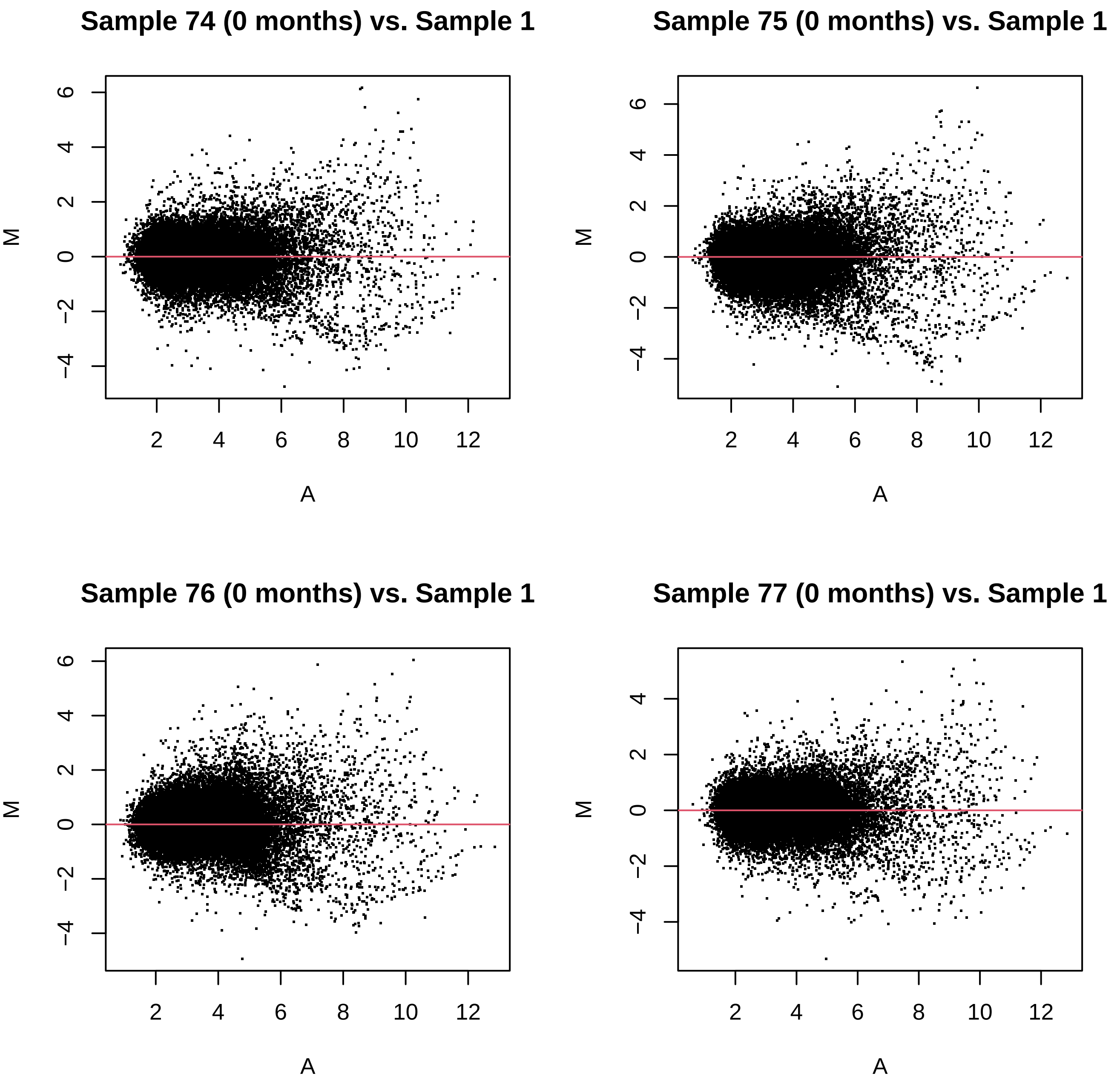
Sample 74 (0 months) vs. Sample 1
Sample 75 (0 months) vs. Sample 1
Sample 76 (0 months) vs. Sample 1
Sample 77 (0 months) vs. Sample 1
M
A

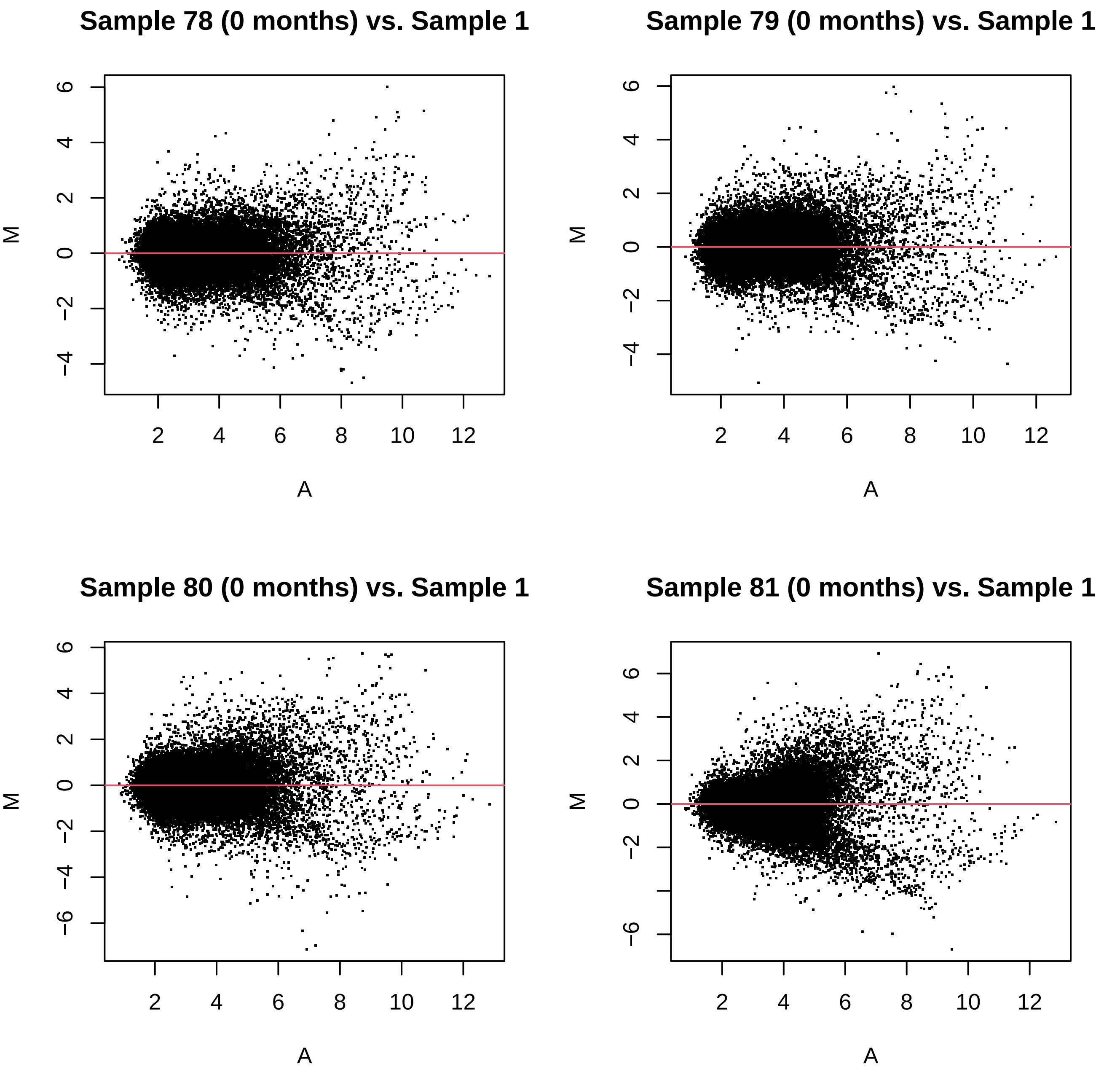

Sample 78 (0 months) vs. Sample 1
Sample 79 (0 months) vs. Sample 1
Sample 80 (0 months) vs. Sample 1
Sample 81 (0 months) vs. Sample 1
M
A

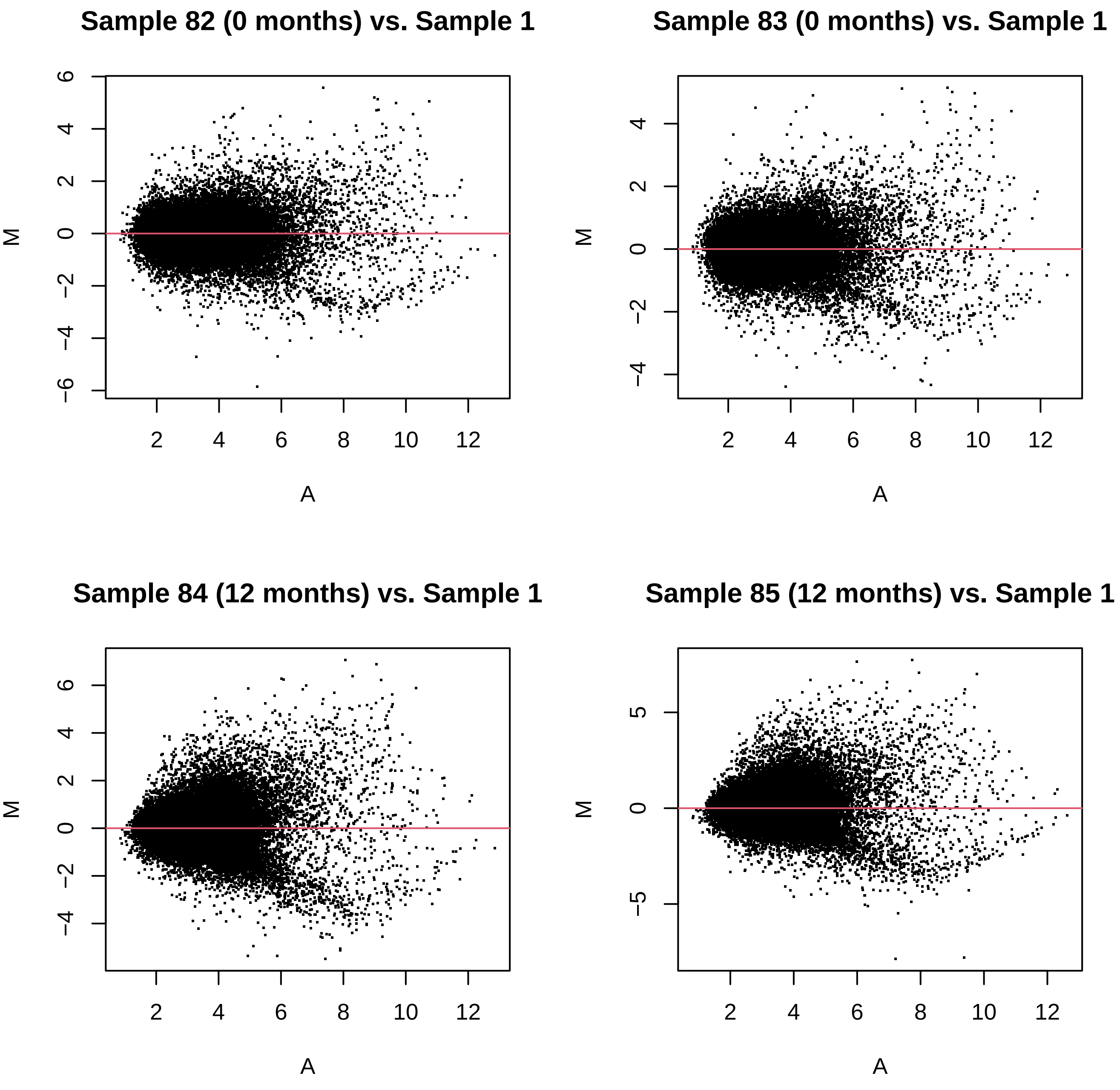
Sample 82 (0 months) vs. Sample 1
Sample 83 (0 months) vs. Sample 1
Sample 84 (12 months) vs. Sample 1
Sample 85 (12 months) vs. Sample 1
M
A
2
4
6
8
10
12
−6
−4
−2
0
5
−5

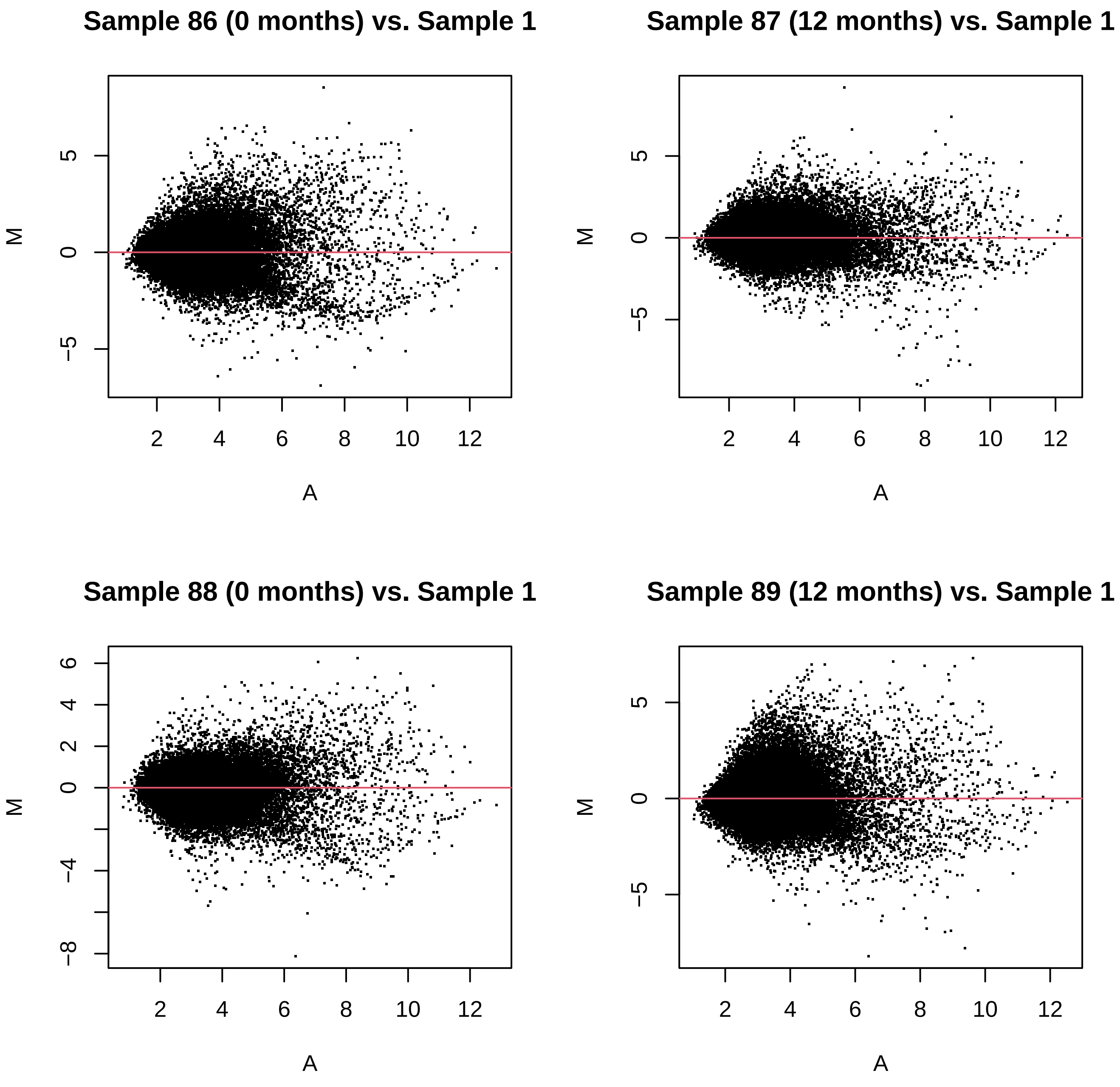
Sample 86 (0 months) vs. Sample 1
Sample 87 (12 months) vs. Sample 1
Sample 88 (0 months) vs. Sample 1
Sample 89 (12 months) vs. Sample 1
M
A
−8
−5
−4
0
2
4
5
6
8
10
12

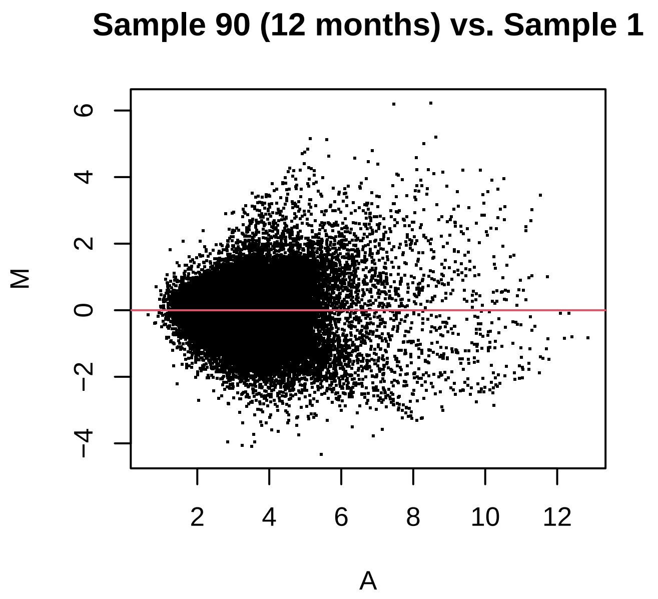
Sample 90 (12 months) vs. Sample 1
M
A
6
4
2
0
−2
−4
2
4
6
8
10
12

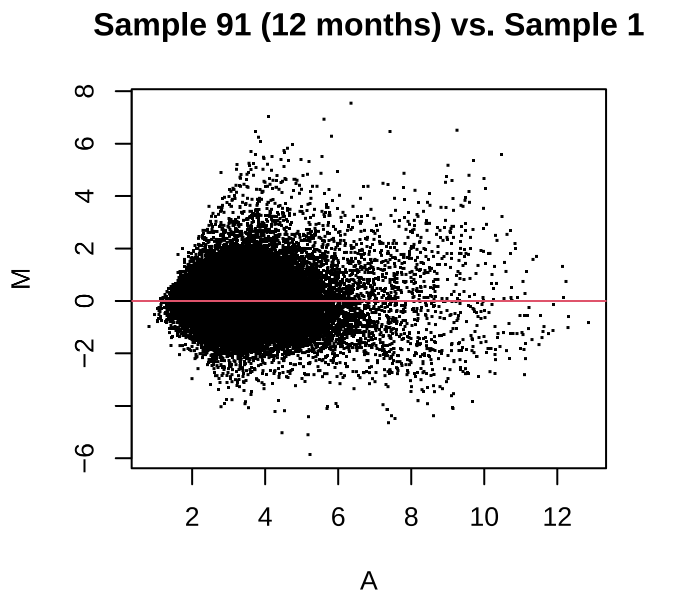
Sample 91 (12 months) vs. Sample 1
M
A
8
6
4
2
0
−2
−6
2
4
6
8
10
12

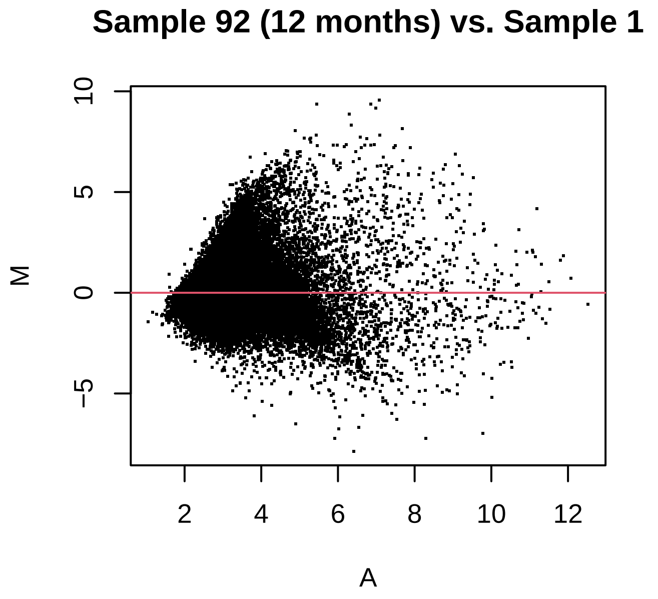
Sample 92 (12 months) vs. Sample 1
M
A
10
5
0
−5
2
4
6
8
10
12

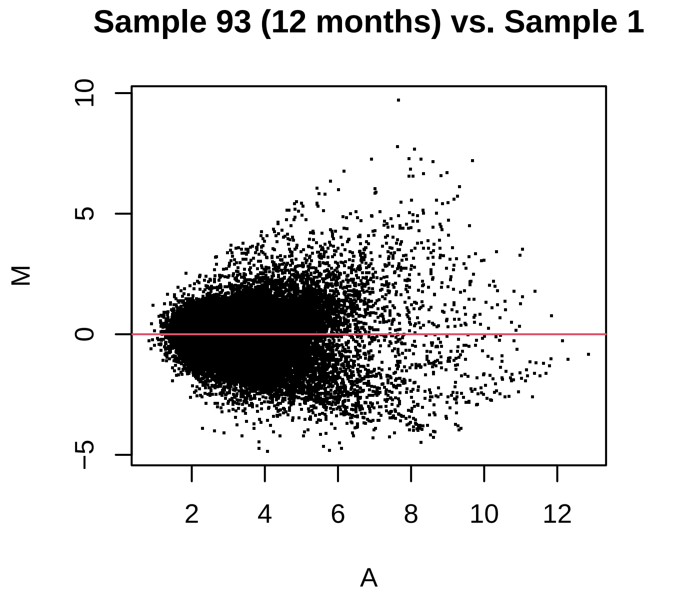
Sample 93 (12 months) vs. Sample 1
M
A
10
5
0
−5
2
4
6
8
10
12

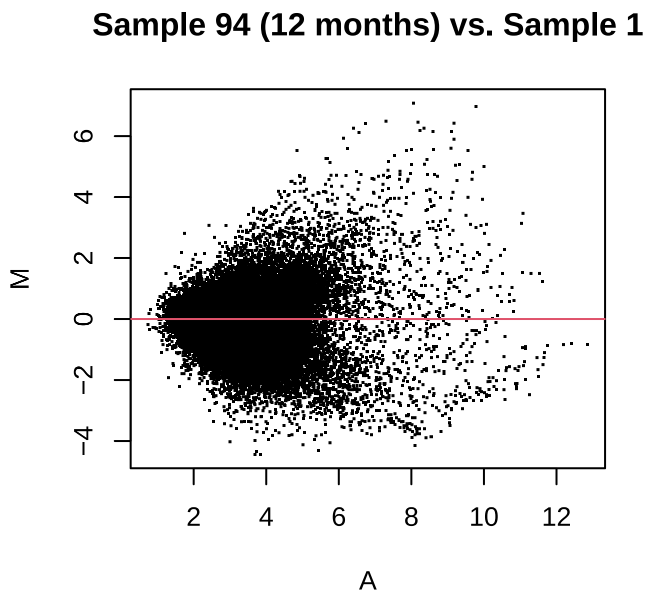
Sample 94 (12 months) vs. Sample 1
M
A
−4
−2
0
2
4
6
2
4
6
8
10
12

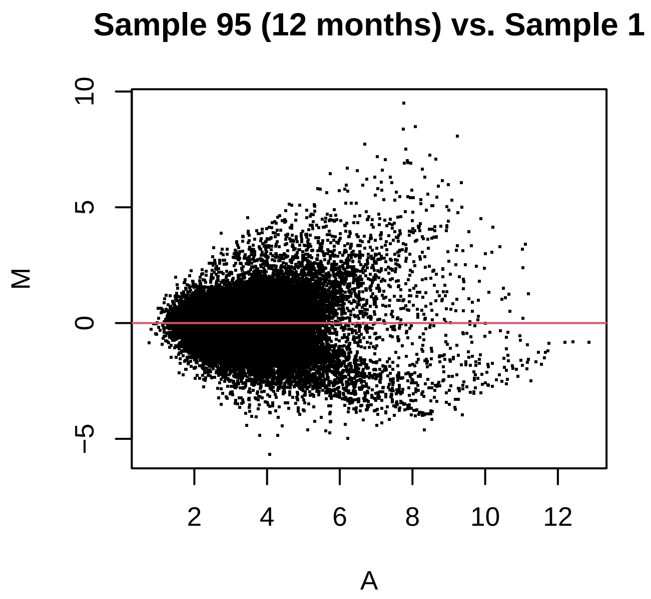
Sample 95 (12 months) vs. Sample 1
M
A
−5
0
5
10
2
4
6
8
10
12

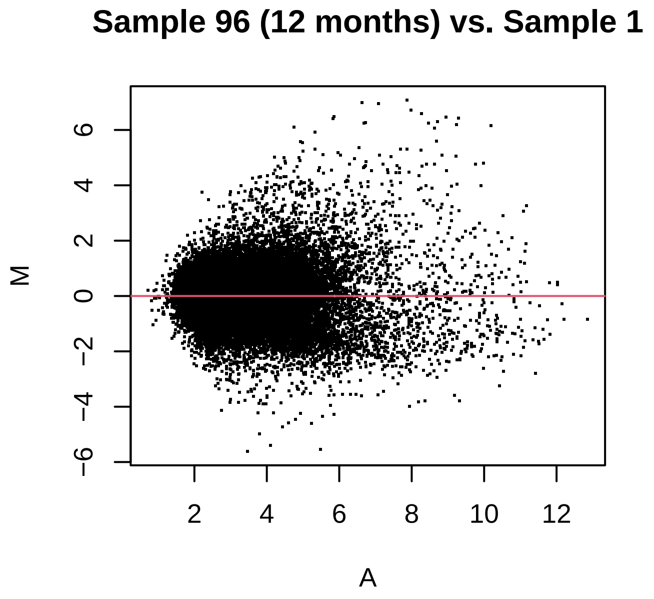
Sample 96 (12 months) vs. Sample 1
M
A
−6
−4
−2
0
2
4
6
2
4
6
8
10
12

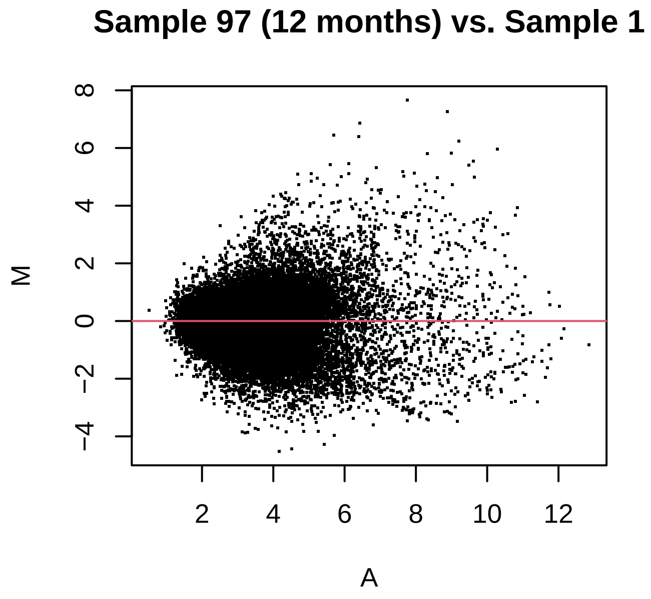
Sample 97 (12 months) vs. Sample 1
M
A
−4
−2
0
2
4
6
8
2
4
6
8
10
12

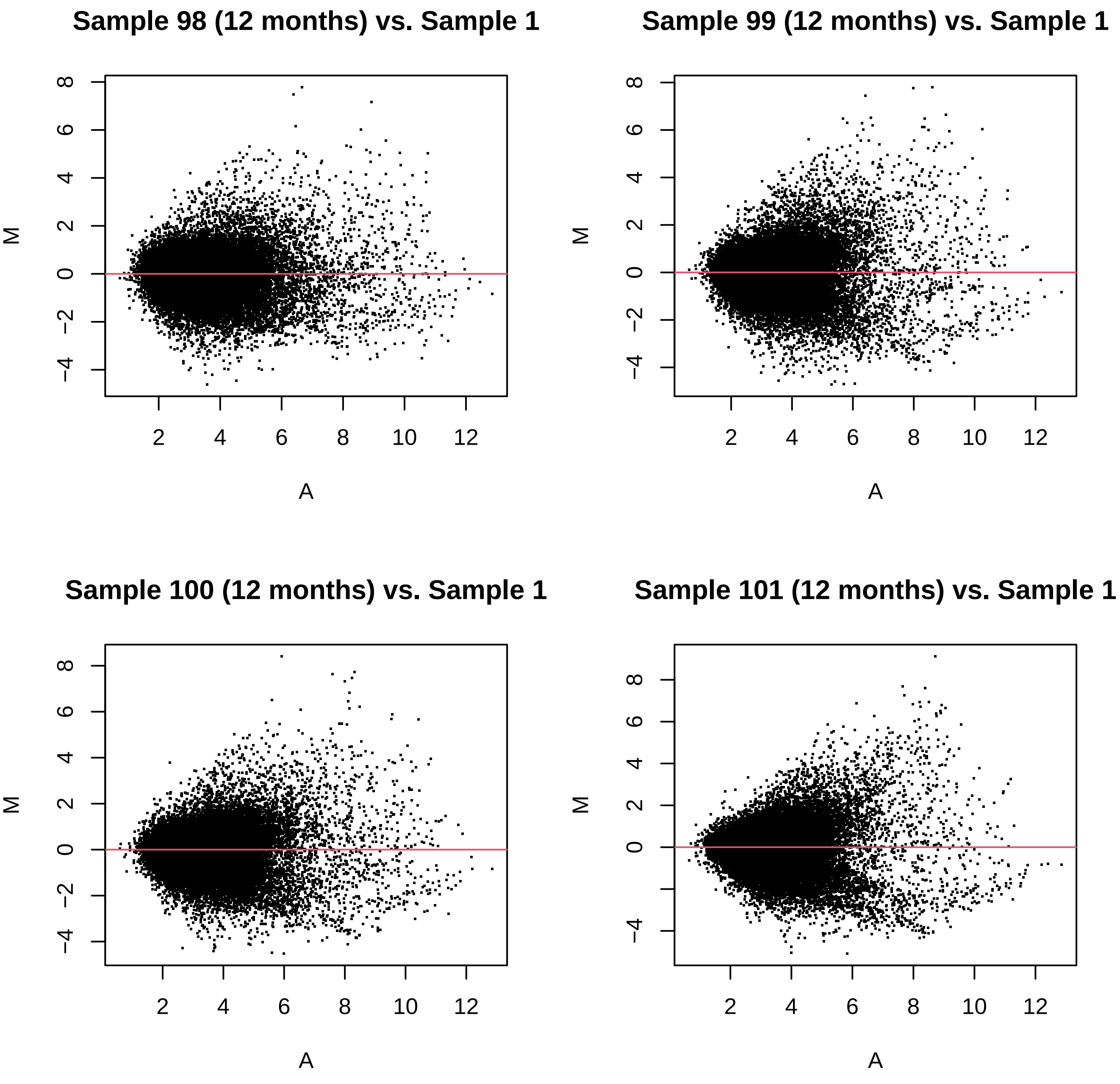
Sample 98 (12 months) vs. Sample 1
Sample 99 (12 months) vs. Sample 1
Sample 100 (12 months) vs. Sample 1
Sample 101 (12 months) vs. Sample 1
M
A
−4
−2
0
2
4
6
8
2
4
6
8
10
12

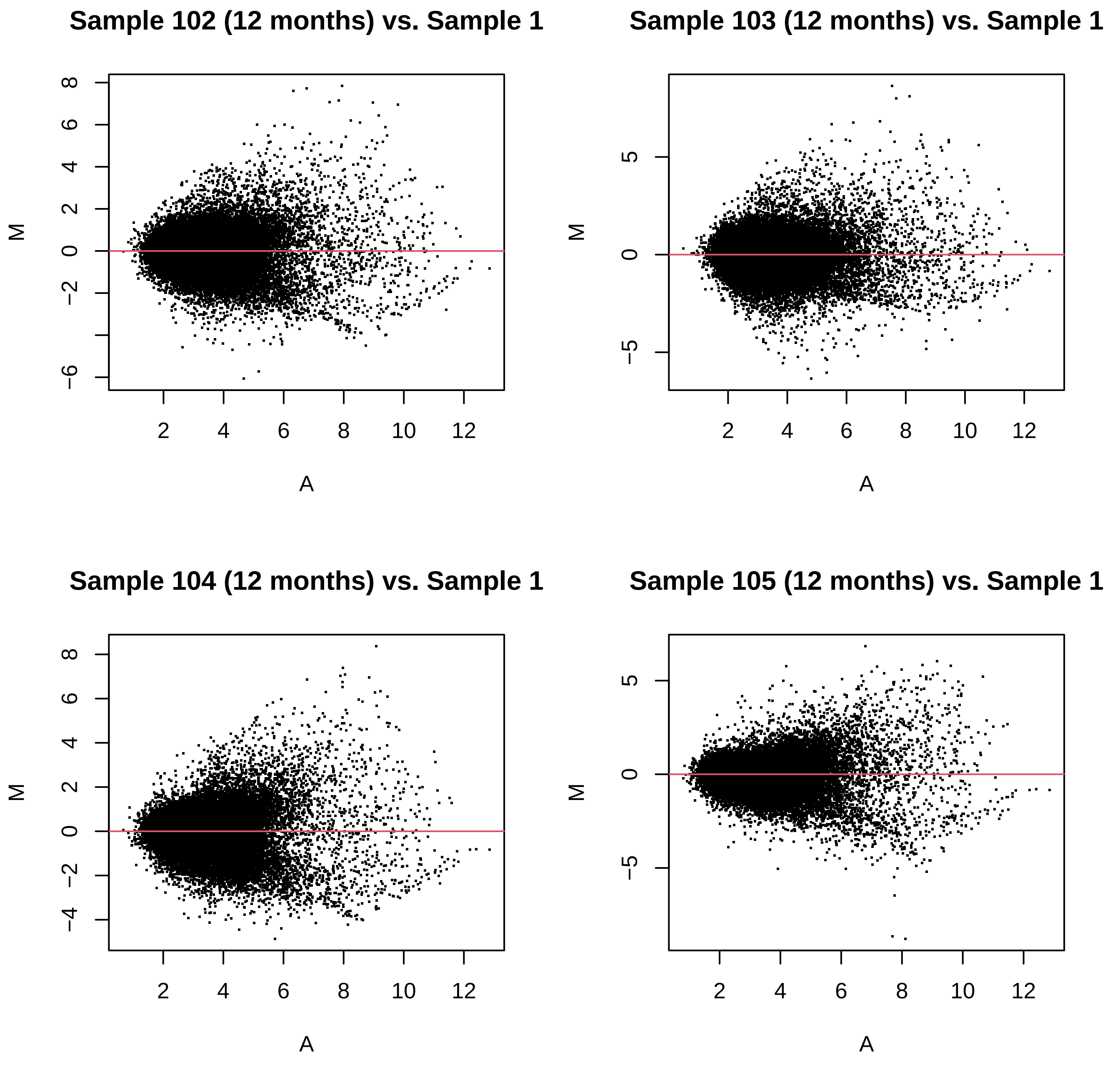
Sample 102 (12 months) vs. Sample 1
Sample 103 (12 months) vs. Sample 1
Sample 104 (12 months) vs. Sample 1
Sample 105 (12 months) vs. Sample 1
M
A
2
4
6
8
10
12

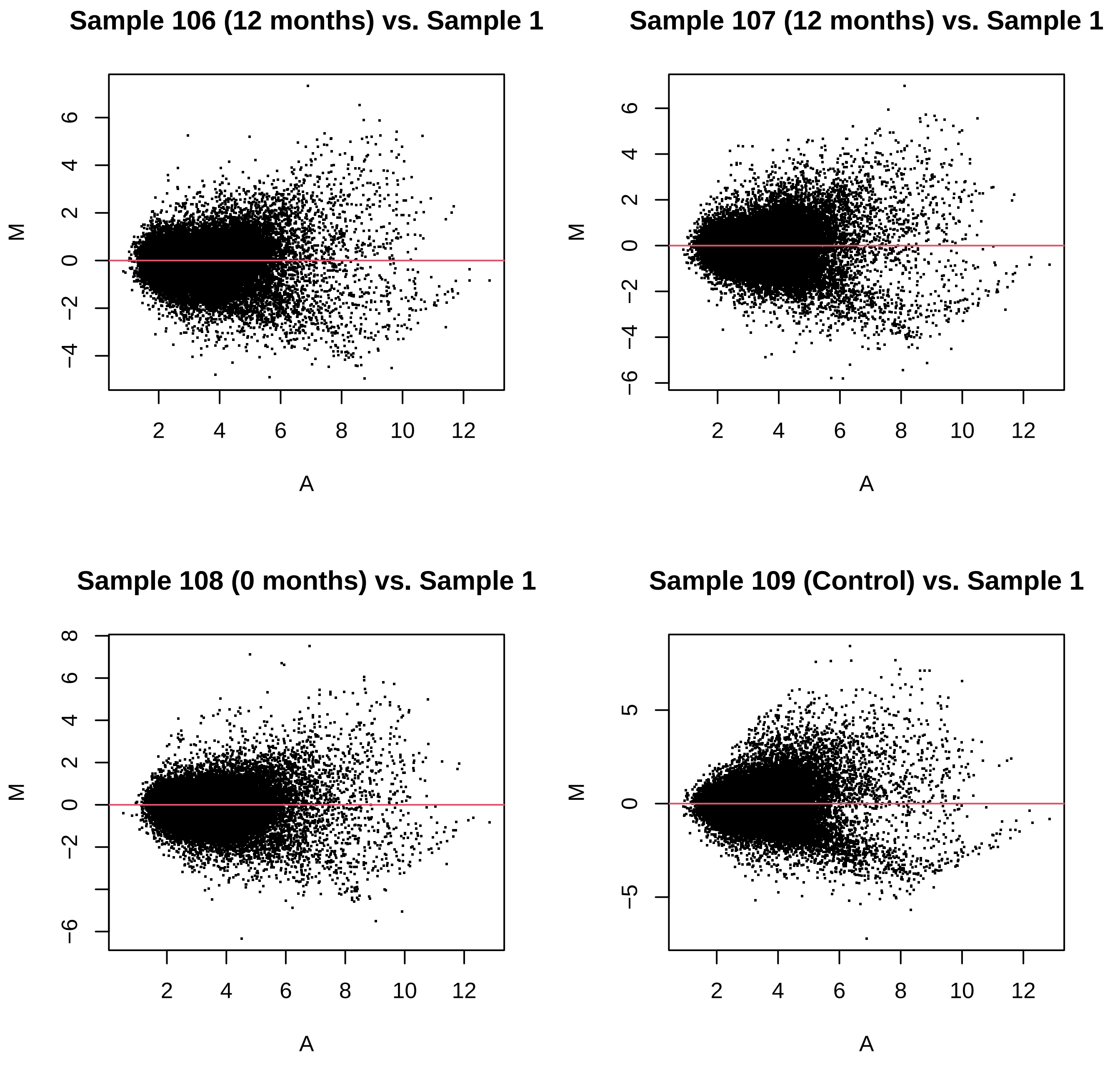
Sample 106 (12 months) vs. Sample 1
Sample 107 (12 months) vs. Sample 1
Sample 108 (0 months) vs. Sample 1
Sample 109 (Control) vs. Sample 1
M
A

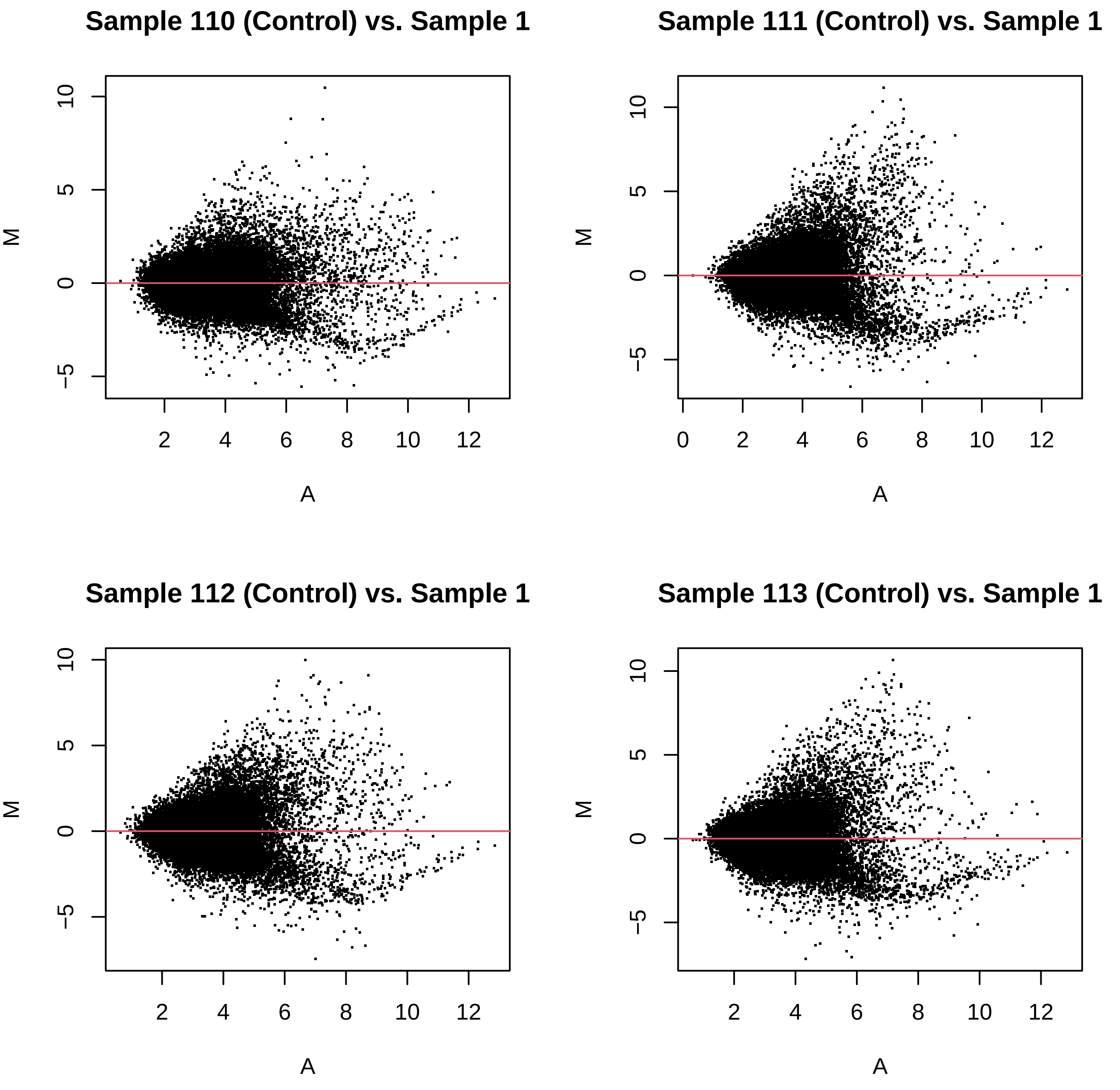
Sample 110 (Control) vs. Sample 1
Sample 111 (Control) vs. Sample 1
Sample 112 (Control) vs. Sample 1
Sample 113 (Control) vs. Sample 1
M
A
−5
0
5
10
0
2
4
6
8
10
12

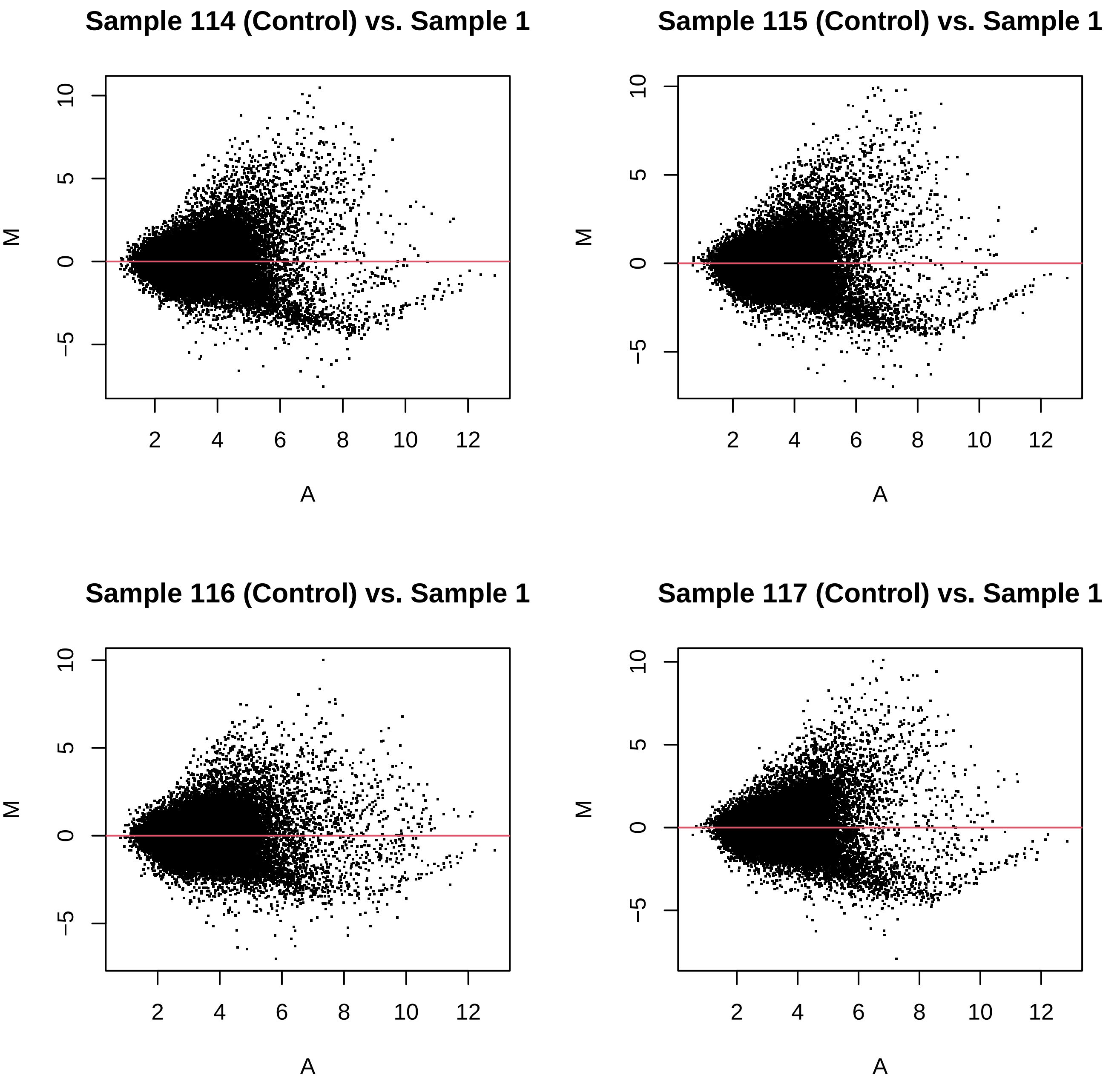
Sample 114 (Control) vs. Sample 1
Sample 115 (Control) vs. Sample 1
Sample 116 (Control) vs. Sample 1
Sample 117 (Control) vs. Sample 1
M
A
10
5
0
−5
2
4
6
8
10
12

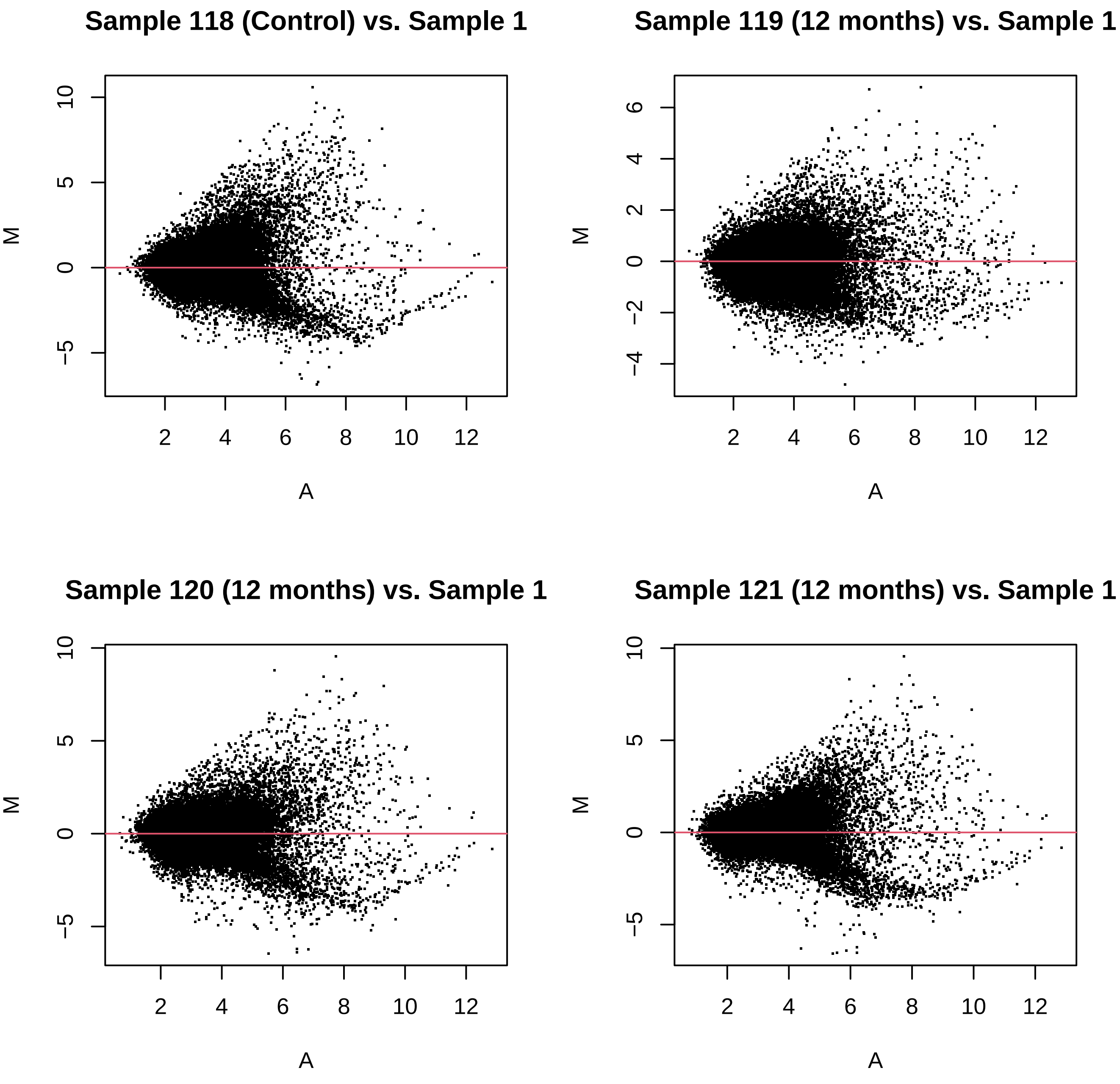
Sample 118 (Control) vs. Sample 1
Sample 119 (12 months) vs. Sample 1
Sample 120 (12 months) vs. Sample 1
Sample 121 (12 months) vs. Sample 1
M
A

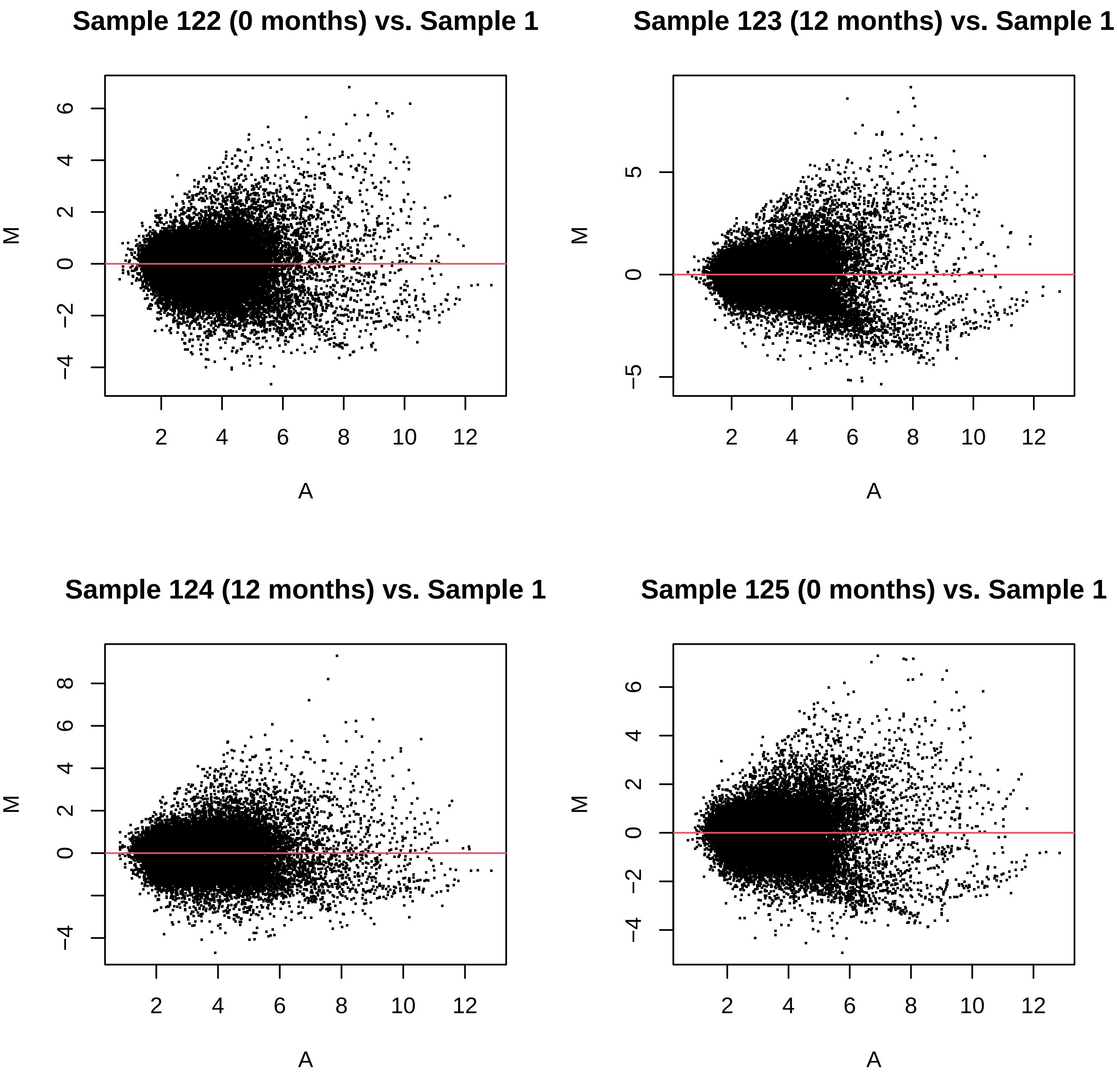
Sample 122 (0 months) vs. Sample 1
Sample 123 (12 months) vs. Sample 1
Sample 124 (12 months) vs. Sample 1
Sample 125 (0 months) vs. Sample 1
M
A

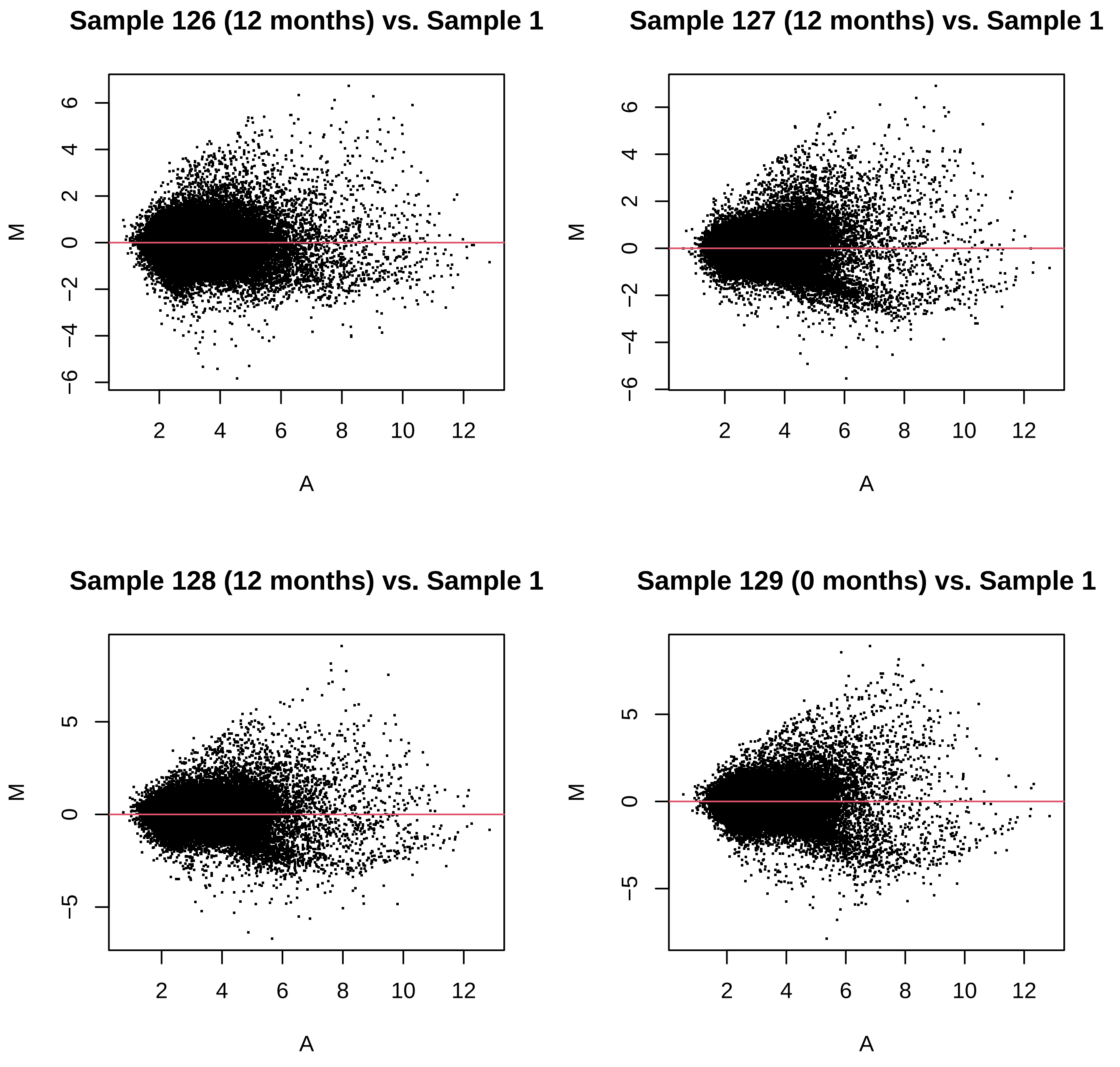
Sample 126 (12 months) vs. Sample 1
Sample 127 (12 months) vs. Sample 1
Sample 128 (12 months) vs. Sample 1
Sample 129 (0 months) vs. Sample 1
M
A
−6
−4
−2
0
2
4
6
−5
5
8
10
12

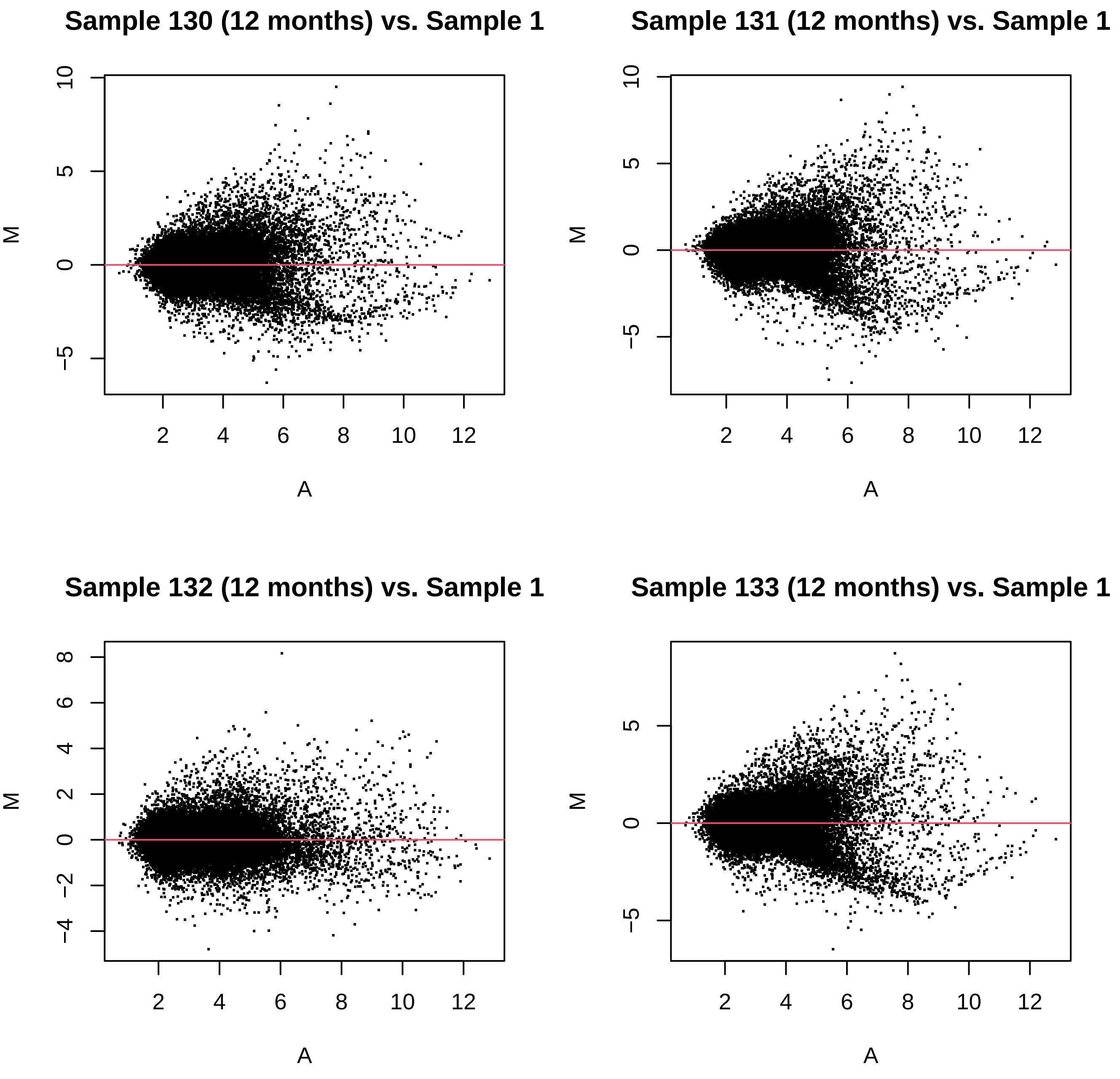
Sample 130 (12 months) vs. Sample 1
Sample 131 (12 months) vs. Sample 1
Sample 132 (12 months) vs. Sample 1
Sample 133 (12 months) vs. Sample 1
M
A
−5
0
5
10
−4
−2
2
4
6
8
12

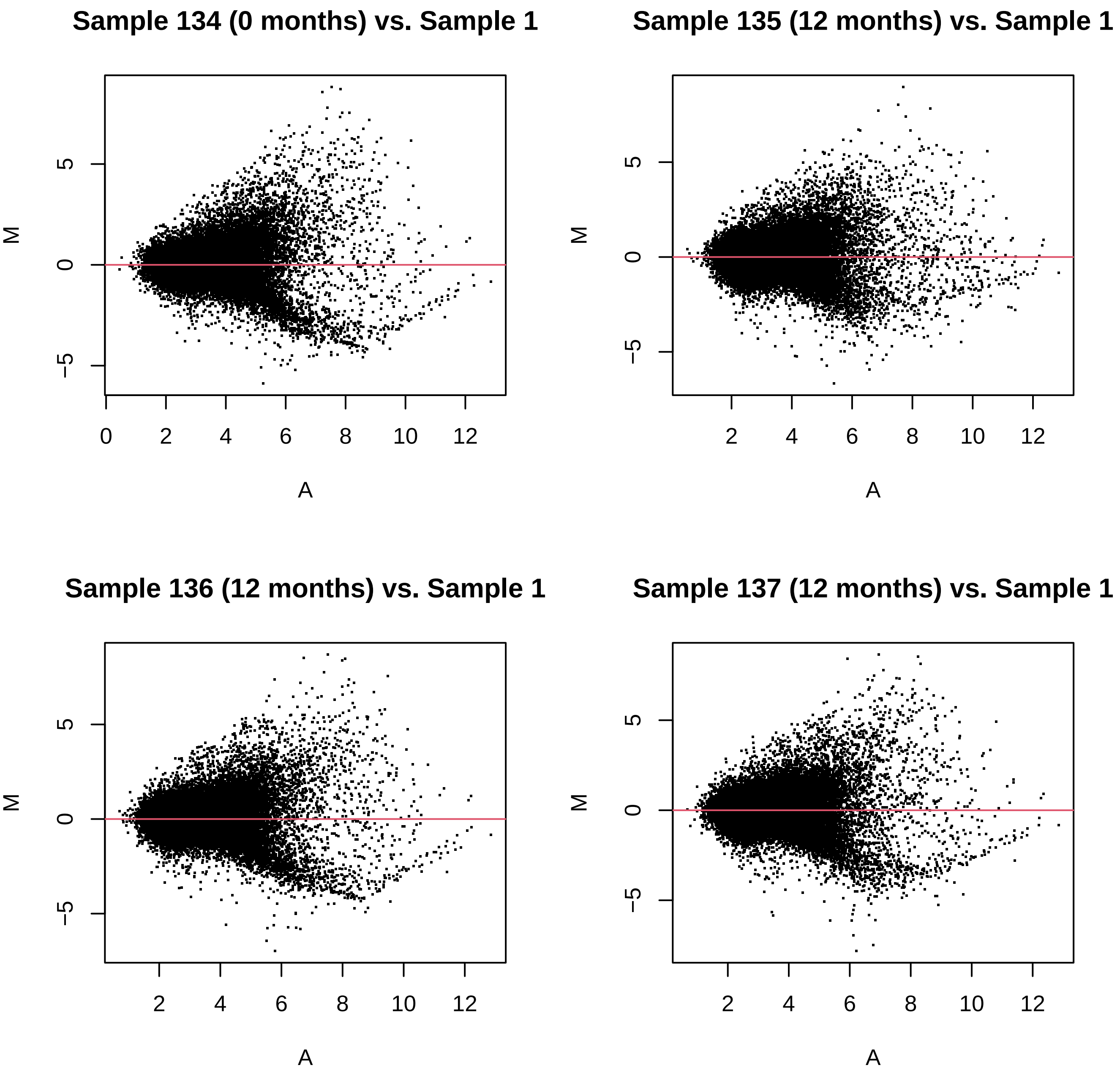
Sample 134 (0 months) vs. Sample 1
Sample 135 (12 months) vs. Sample 1
Sample 136 (12 months) vs. Sample 1
Sample 137 (12 months) vs. Sample 1
M
A
−5
0
5
0
2
4
6
8
10
12

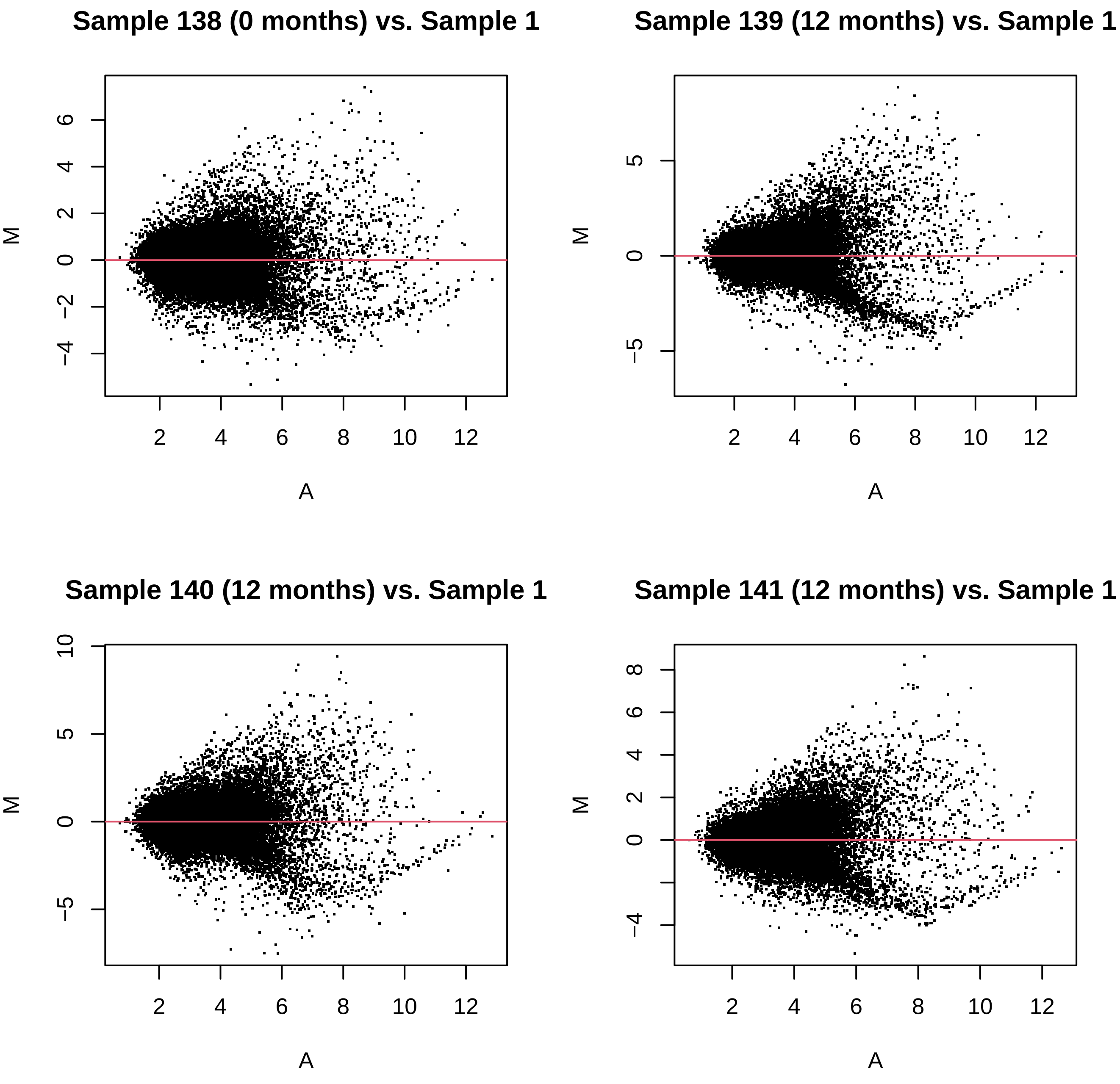
Sample 138 (0 months) vs. Sample 1
Sample 139 (12 months) vs. Sample 1
Sample 140 (12 months) vs. Sample 1
Sample 141 (12 months) vs. Sample 1
M
A

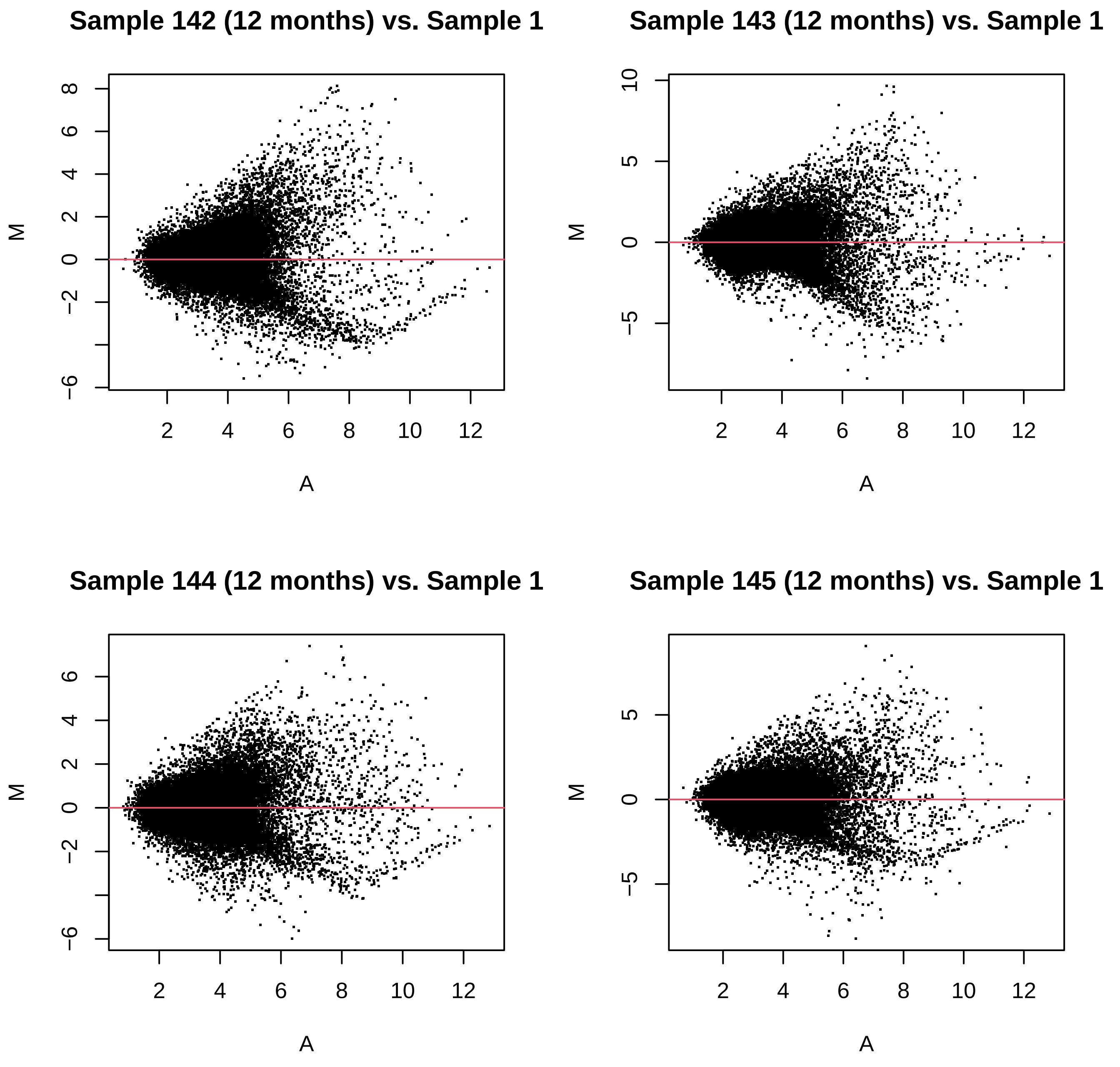
Sample 142 (12 months) vs. Sample 1
Sample 143 (12 months) vs. Sample 1
Sample 144 (12 months) vs. Sample 1
Sample 145 (12 months) vs. Sample 1
M
A

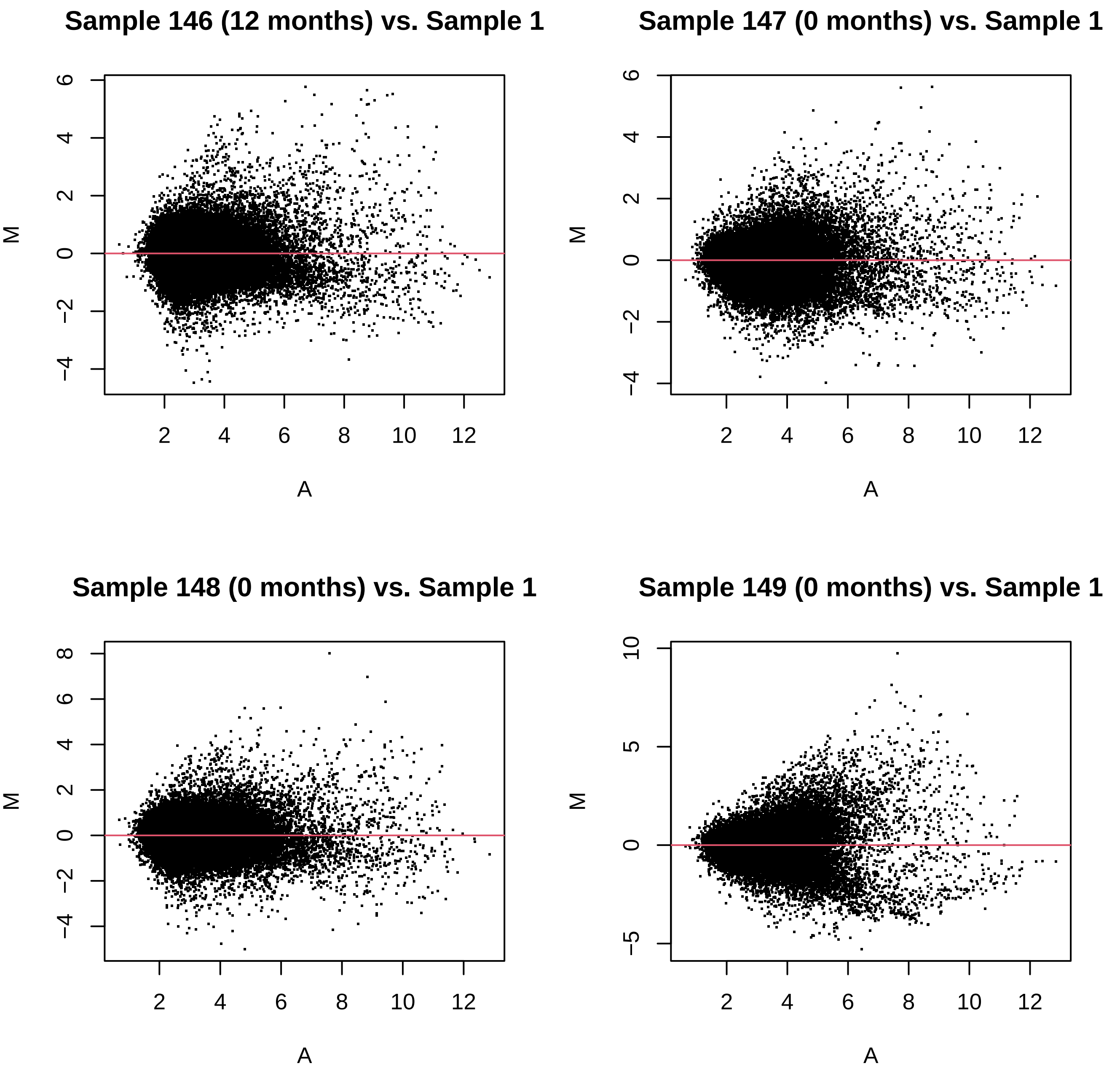
Sample 146 (12 months) vs. Sample 1
Sample 147 (0 months) vs. Sample 1
Sample 148 (0 months) vs. Sample 1
Sample 149 (0 months) vs. Sample 1
M
A

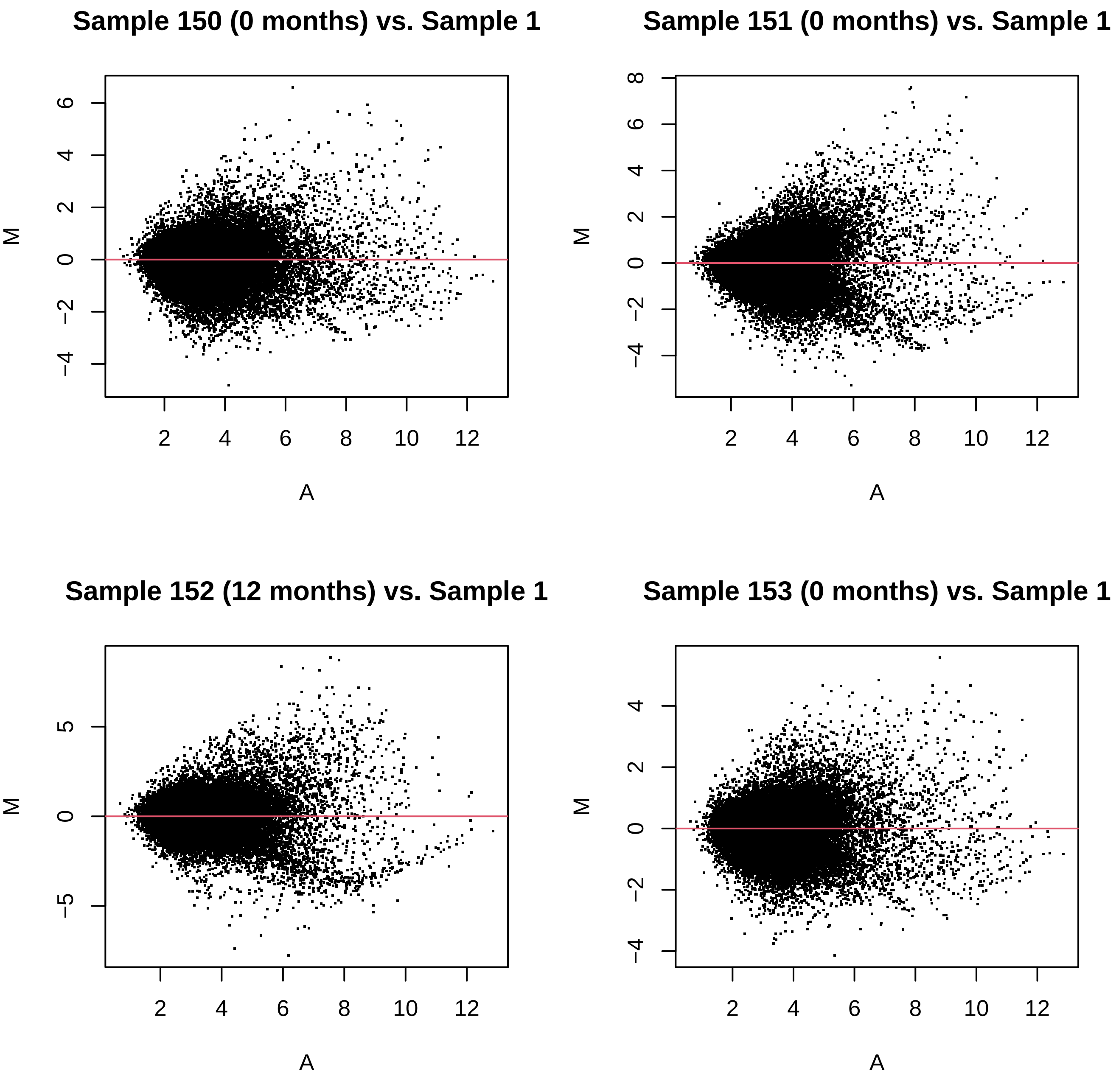

Sample 150 (0 months) vs. Sample 1
Sample 151 (0 months) vs. Sample 1
Sample 152 (12 months) vs. Sample 1
Sample 153 (0 months) vs. Sample 1
M
A

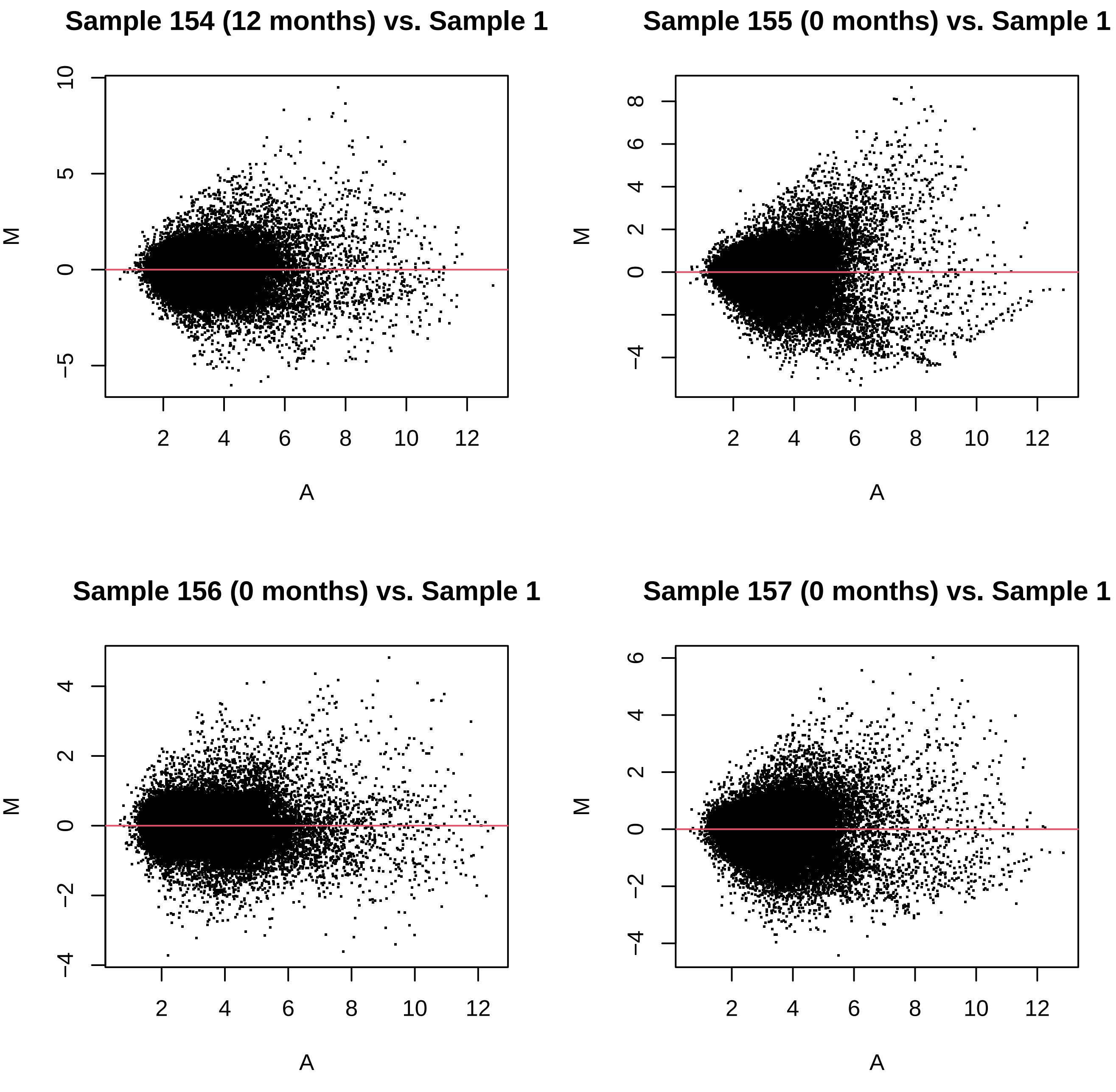
Sample 154 (12 months) vs. Sample 1
Sample 155 (0 months) vs. Sample 1
Sample 156 (0 months) vs. Sample 1
Sample 157 (0 months) vs. Sample 1
M
A
10
5
0
−5
8
6
4
2
−4
−2
12

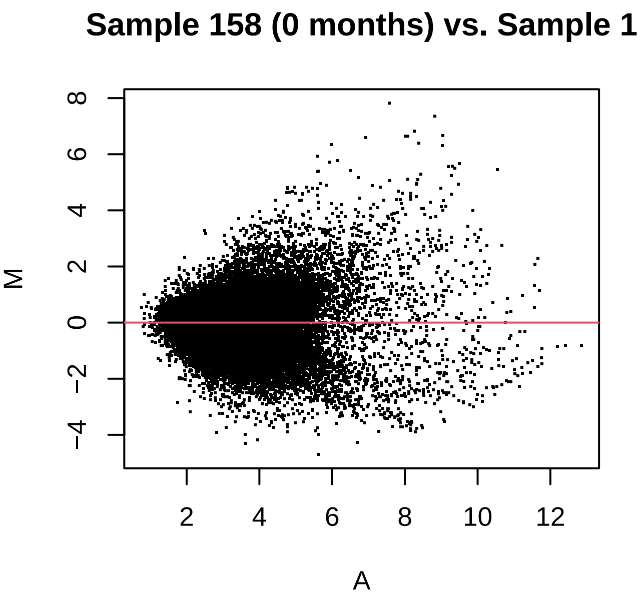
Sample 158 (0 months) vs. Sample 1
M
A
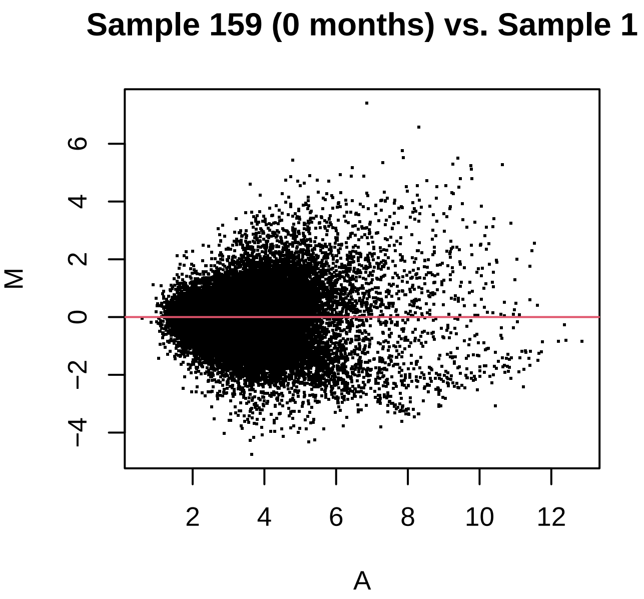
Sample 159 (0 months) vs. Sample 1
M
A
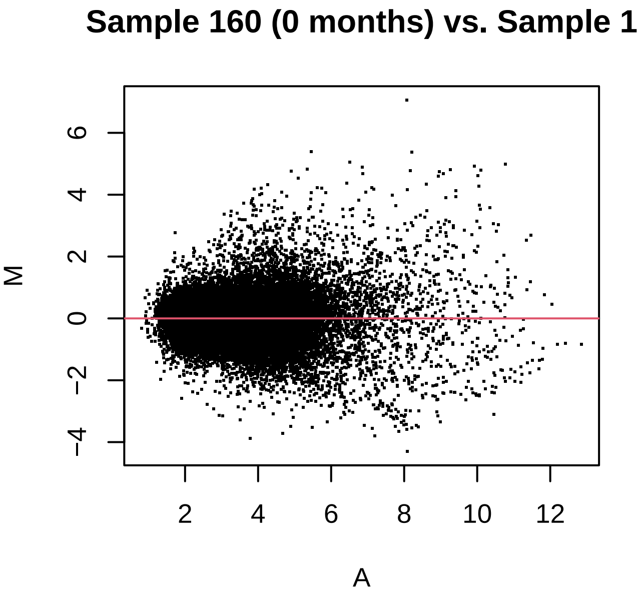
Sample 160 (0 months) vs. Sample 1
M
A
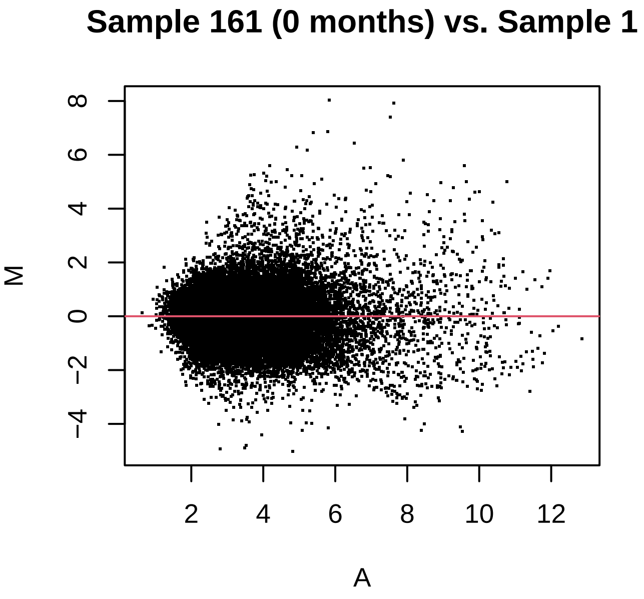
Sample 161 (0 months) vs. Sample 1
M
A

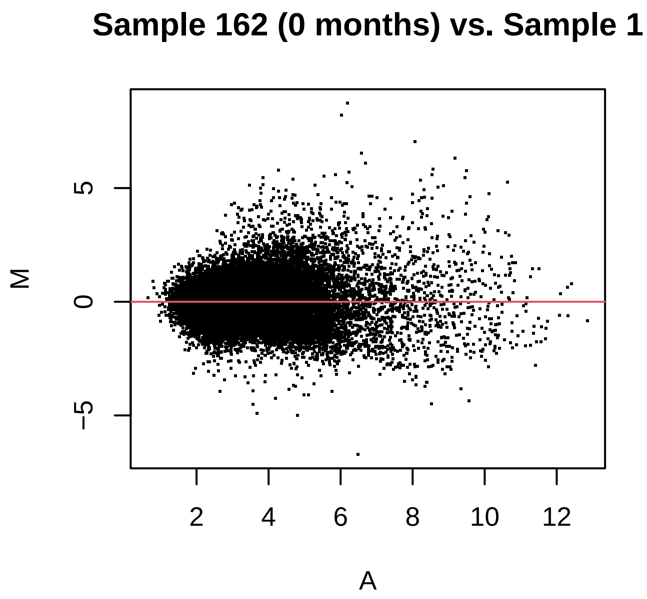
Sample 162 (0 months) vs. Sample 1
M
−5
0
5
2
4
6
8
10
12
A

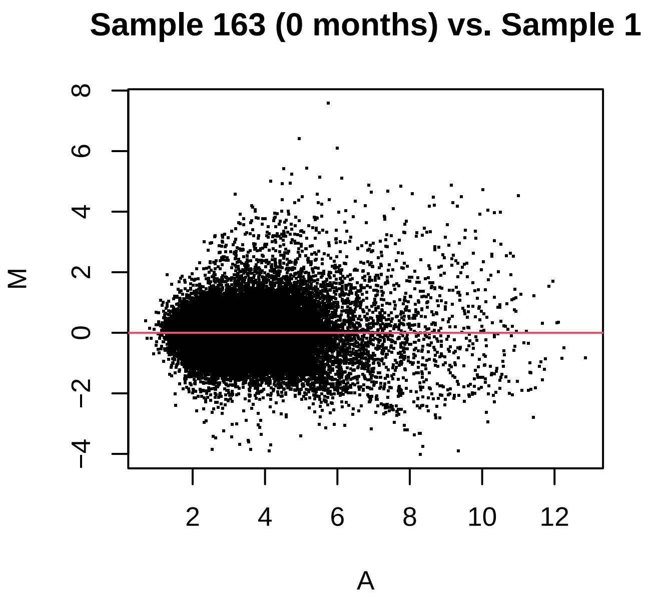
Sample 163 (0 months) vs. Sample 1
M
−4
−2
0
2
4
6
8
2
4
6
8
10
12
A

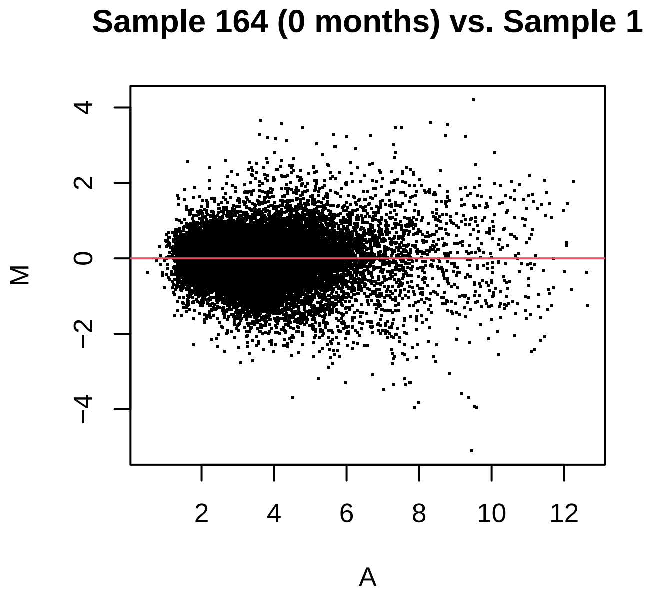
Sample 164 (0 months) vs. Sample 1
M
−4
−2
0
2
4
2
4
6
8
10
12
A

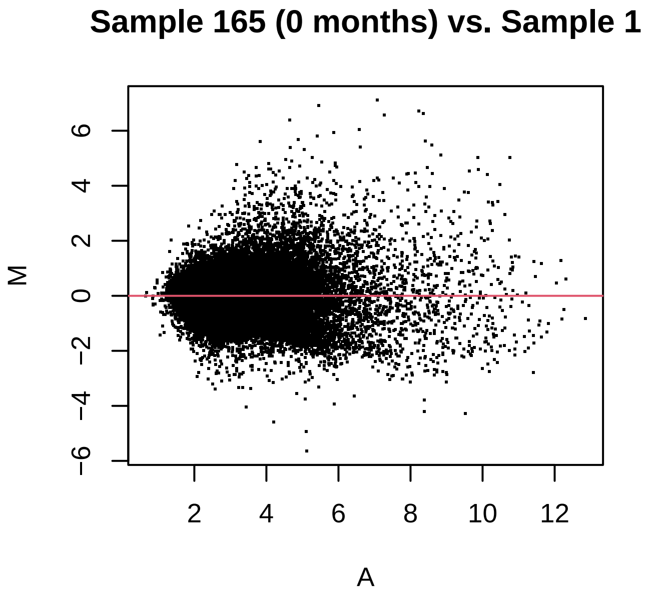
Sample 165 (0 months) vs. Sample 1
M
−6
−4
−2
0
2
4
6
2
4
6
8
10
12
A

Sample 166 (0 months) vs. Sample 1

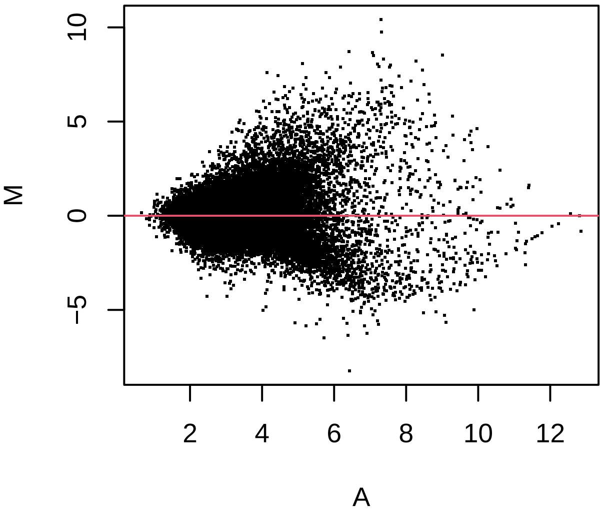

Sample 167 (0 months) vs. Sample 1

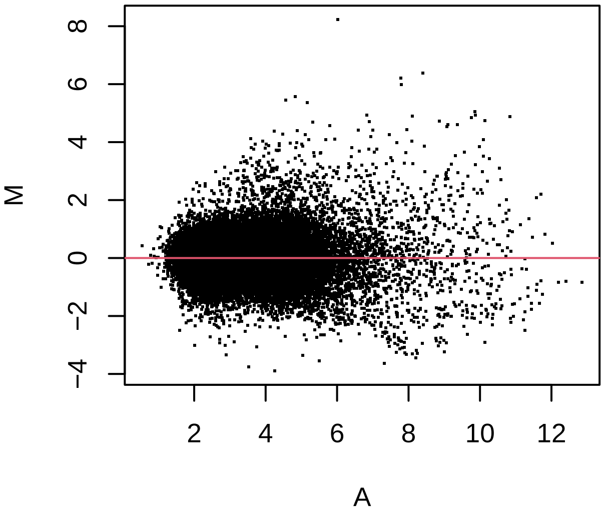

Sample 168 (12 months) vs. Sample 1

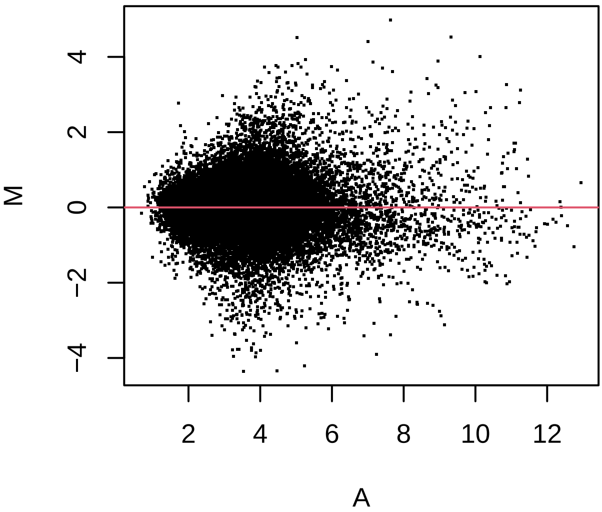

Supplement: Supplementary file 1 [file cancers-17-01022-s001.zip › Graphical plots S1 PCA and MvAplot.pdf]
